# Impact of Canterbury earthquakes on well‐being in New Zealand

**DOI:** 10.1111/disa.12692

**Published:** 2025-06-26

**Authors:** Thi Mui Nguyen, Ilan Noy, Yigit Saglam

**Affiliations:** ^1^ Victoria University of Wellington New Zealand

**Keywords:** Canterbury earthquakes, difference‐in‐difference‐in‐difference, health, life satisfaction, seismic risk, well‐being

## Abstract

This study examines the impacts of earthquakes on individual subjective well‐being, using data from New Zealand's General Social Survey and a difference‐in‐difference‐in‐difference approach. By matching this with Earthquake Commission insurance claims, we could analyse the micro‐level effects of the 2010–11 Canterbury (New Zealand) earthquakes. Our findings reveal that life satisfaction consistently declined in affected areas, emphasising its sensitivity to catastrophic shocks. Narrowly‐defined areas exhibited negative impacts across all well‐being indicators. Vulnerable groups, including Māori, faced significant declines in life satisfaction, while younger people, migrants, and employed individuals demonstrated resilience. Economic well‐being decreased notably for females and younger persons in Christchurch, whereas Māori and employed individuals showed increases. Perceptions of safety weakened, particularly among females and the employed, but strengthened among sole individuals with dependent children and migrants. These results highlight the hidden adverse impacts of earthquakes and underscore the necessity for targeted interventions and support mechanisms tailored to vulnerable populations to mitigate disasters' effects on well‐being effectively.

## 1 INTRODUCTION

The Canterbury earthquake sequence (CES), a series of seismic events that struck the Canterbury Region of New Zealand in 2010–11, left an indelible mark on the affected population. The two most significant tremors were the Darfield earthquake on 4 September 2010, with a magnitude of 7.1, and the Christchurch earthquake on 22 February 2011, with a magnitude of 6.3. The CES resulted in widespread destruction and losses and had profound socioeconomic repercussions. It caused 185 fatalities and more than 6,500 injuries (King et al., 2014; Potter et al., 2015), led to the destruction of countless homes, and damaged roughly 2,000 downtown commercial, residential, and office buildings, including numerous structures of historical significance (Chang et al., 2014). The CES also significantly damaged the road network and the underground horizonal infrastructure, affecting the provision of water, power, and wastewater services (Rogers et al., 2014; van Ballegooy et al., 2014).

Here, we focus not on the material damage associated with the CES but on its implications for well‐being. We use six waves of New Zealand's General Social Survey: two from before the CES (2008 and 2010) and four from afterwards (2012, 2014, 2016, and 2018). We utilise residential insurance data from the public insurer to identify the areas most affected by the CES. In addition to the standard difference‐in‐differences (DiD) regression, we use the difference‐in‐difference‐in‐difference (DDD) approach, which provides us with more nuance about the effects of the earthquakes, on different groups, than the standard DiD (Williams, 2006; Olden and Møen, 2022).

The two main objectives of our study are: (i) to investigate how the CES has influenced the well‐being of individuals in New Zealand in the long term; and (ii) to determine whether the earthquakes' impact was different across different sociodemographic groups. Our paper contributes to the literature in three significant ways. First, it evaluates the long‐term effects of a disaster on well‐being using a large and representative dataset that includes well‐being data from more than 50,000 households across New Zealand—a much longer time frame and a larger sample than in previous studies. Second, we analyse the impact of the earthquakes on a wide range of well‐being indicators, including subjective well‐being, health, economic security, and safety, whereas previous research has focused on mostly emotional (mental health) well‐being. Third, by employing a DDD approach with a three‐way interaction term and a large sample, we can distinguish differences in the impact of earthquakes across various demographic groups.

The remainder of the paper is structured as follows: section 2 contains a review of the literature on the impact of disasters on well‐being; section 3 describes the data and the sample; sections 4 and 7 sets out the methodology; section 5 presents the empirical results; section 6 provides a robustness check; and section 7 sums up the study in a conclusion.

## 2 LITERATURE REVIEW

**FIGURE 1:**
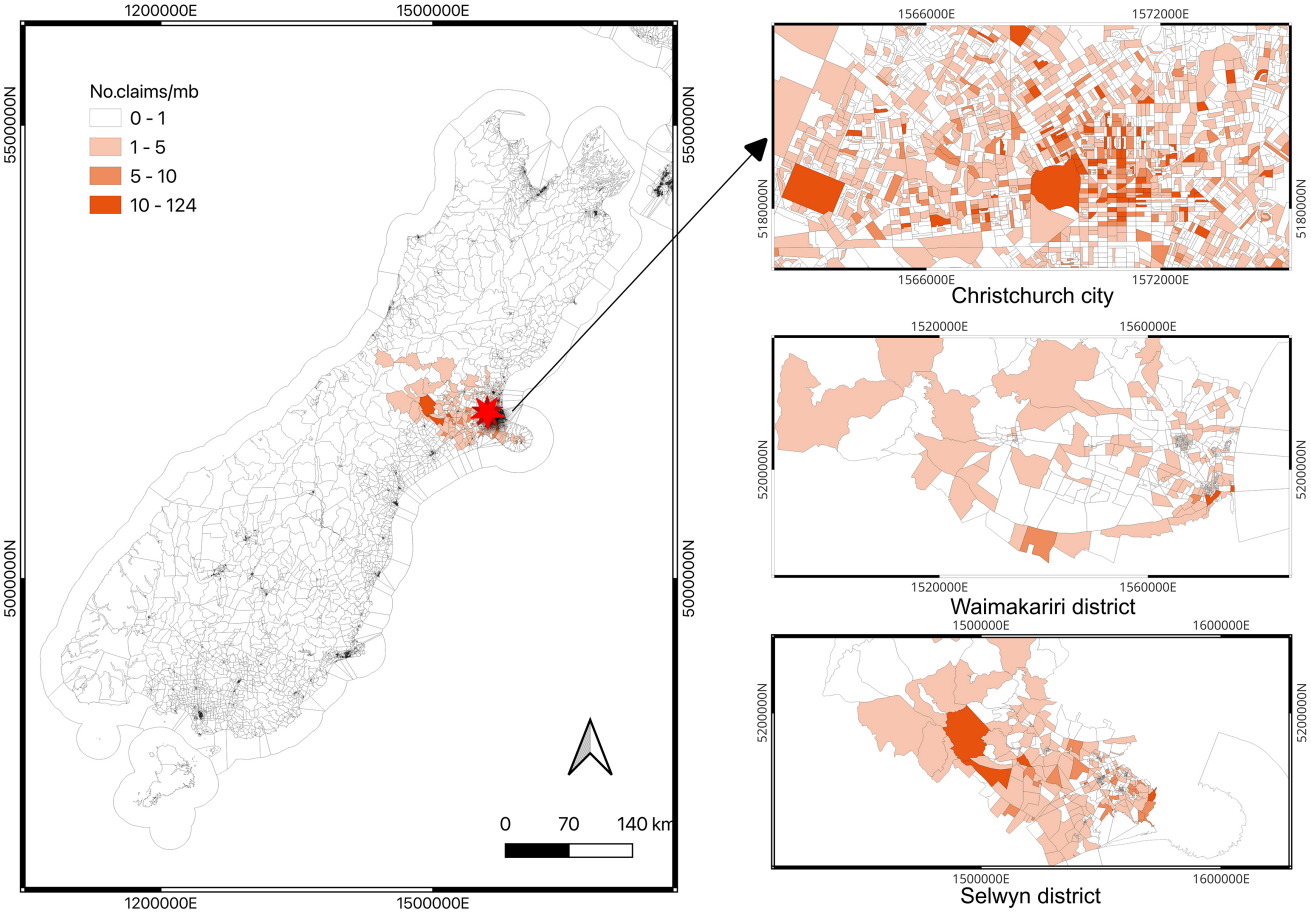
Identification of meshblocks affected by the CES in 2010–11. **Note:** No.claims/mb = Number of claims per meshblock. **Source:** authors.

Establishing a clear definition of well‐being poses a considerable challenge, as it is a concept that varies across cultures, disciplines, and individual perspectives (Dodge et al., 2012). According to Ruggeri et al. (2020), well‐being is not only happiness or life satisfaction but also includes aspects of physical health, psychological state, social connections, and economic stability. Here, we utilise well‐being indicators as defined by the Living Standards Framework (LSF)^1^ of The Treasury of New Zealand. Inspired by the OECD (Organisation for Economic Co‐operation and Development) Better Life Index (OECD, 2011), the LSF supports a well‐being‐focused policy strategy (The Treasury, 2018). It features 12 domains that capture the present state of well‐being (for more details see Figure 1 in the Appendix in the supplementary materials).^2^ We focus on four specific well‐being indicators: (i) life satisfaction; (ii) general health; (iii) income adequacy; and (iv) safety. By concentrating on these, we aim to provide a comprehensive assessment of well‐being that aligns with the LSF and uses whatever data are available. These indicators collectively address key aspects of well‐being. Further explanation of the selection of these specific indicators of well‐being is provided in subsection 3.2.

Some studies have focused on the well‐being impact of disasters triggered by natural hazards, such as droughts (Carroll, Frijters, and Shields, 2009; Rigby et al., 2011; Bryan et al., 2020; Berlemann and Eurich, 2022), floods (Luechinger and Raschky, 2009; Walker‐Springett, Butler, and Adger, 2017; Hudson et al., 2019; Twiddy, Trump, and Ramsden, 2022; Murata et al., 2023), and hurricanes (Kimball et al., 2006; LaJoie, Sprang, and McKinney, 2010; Berlemann, 2016). Unlike these phenomena, which can frequently be forecasted (at least some days in advance), earthquakes remain largely unpredictable, even when certain regions are considered to be more seismically active.^3^

The 2011 Great East Japan Earthquake, which also spawned a tsunami and a nuclear accident, had diverse impacts on well‐being. Sugano (2016) demonstrated that elderly survivors experienced significant psychological distress and changes in subjective well‐being following the disaster. That paper utilised data from the Japanese Study of Aging and Retirement, collected in two waves before and after the event. The study employed a DiD approach to estimate the earthquake's causal impact, with respondents from the city of Sendai serving as the treatment group and those from other Japanese cities as the control group. Similarly, Uchida, Takahashi, and Kawahara (2014) used two large surveys conducted before and after the earthquake to investigate changes in well‐being among the 10,744 participants. Using logit regressions, they found that the earthquake affected overall mental health and induced short‐term negative feelings, even among individuals in regions that were not directly impacted.

In China, Wang and Wang (2023) found that the 2008 Wenchuan earthquake significantly reduced victims' subjective well‐being for nearly a decade. This study utilised six waves of a nationally‐representative dataset from 2005–17 and a DiD approach to pinpoint the short‐ and long‐term causal well‐being effects of the disaster. Liao et al. (2019) measured residents' life satisfaction after the Wenchuan earthquake by collecting data through field surveys performed in Wenchuan and a reference area, Zitong, from 2015–16. A total of 129 residents from the earthquake‐affected area and 131 residents from the non‐affected area completed questionnaires. Interestingly, the authors found that life satisfaction in the earthquake‐hit area was higher, potentially owing to effective post‐disaster restoration and reconstruction efforts.

In Indonesia, De and Thamarapani (2022) examined the long‐term causal effects of the 2006 Yogyakarta earthquake on subjective well‐being. Their data included three survey waves from before the earthquake (1993, 1997, and 2000) and two from after the earthquake (2007 and 2014), utilising the Indonesian Family Life Survey matched with municipality‐level earthquake data from the United States Geological Survey. They employed a DiD methodology, revealing that individuals exposed to the earthquake experienced significant reductions in subjective well‐being even eight years after the disaster. The population in municipalities struck by the earthquake was the treated group, whereas the population in other municipalities served as the control group.

In New Zealand, most studies on the impact of disasters on well‐being focus on mental health as documented by health service providers. For instance, Beaglehole et al. (2019, 2020) used longitudinal aggregated data available for residents under the Canterbury District Health Board and the rest of New Zealand from 2008–18. Both studies compared the elderly and children in Canterbury with national figures (excluding Canterbury) to assess the impact of the CES on mental health. Using a different data source, Begg et al. (2021) tracked the mental well‐being of the population affected by the CES, utilising the Canterbury Wellbeing Survey. This is a cross‐sectional survey of randomly‐selected adults aged 18 and older residing in Christchurch, conducted in 2013, 2015, 2017, 2018 and 2019, and focuses exclusively on the earthquake‐affected population of the region.

Our paper is the first to identify the impact of the CES on different well‐being indicators using nationally‐representative data from 2008–18 and a DDD approach. The latter helps to isolate the impact of the earthquakes from other factors affecting well‐being both locally and nationally.

The data and methodology in our study are closest to those used by Wang and Wang (2023). As mentioned above, Sugano (2016) and De and Thamarapani (2022) also employed a DiD approach; however, the data of Sugano (2016) included only two waves and were sourced from a specialised survey, and while De and Thamarapani (2022) used data from a longitudinal social survey, it was composed of only two post‐earthquake waves: one year and eight years after the disaster (2007 and 2014, respectively). Additionally, the pre‐earthquake data were collected much earlier (1993, 1997, and 2000). Wang and Wang (2023) employed a DiD approach in their study of the 2008 Wenchuan earthquake to determine its causal effects on subjective well‐being. Their study consisted of six waves—two pre‐earthquake waves (2005 and 2006) and four post‐earthquake waves (2010, 2012, 2015, and 2017)—from the Chinese General Social Survey. They matched individuals from Sichuan (the affected area) with similar individuals from non‐affected areas before implementing the DiD regressions.

## 3 DATA

### 3.1 Earthquake Commission (EQC)

The EQC is a government‐owned provider of natural hazard insurance for residential properties.^4^ It provides coverage exclusively for land damage caused by floods or storms, and more comprehensive coverage (including dwellings) for landslips and non‐weather hazards like earthquakes, tsunamis, and volcanic eruptions. The insurance claims dataset we use utilised each claim's location based on geocoordinates. We consider claims that were made three days before and after the date of the CES to detect the earthquake‐affected meshblocks.^5^ We then identify those meshblocks where at least one insurance claim was registered, in the three regions impacted most by the CES: Christchurch city (CHC); Waimakariri District; and Selwyn District. This resulted in 1,544 earthquake‐affected meshblocks in CHC, 128 in Waimakariri District, and 125 in Selwyn District (see Figure 1).

### 3.2 New Zealand's General Social Survey

Data for this study come from Stats NZ's Integrated Data Infrastructure (IDI), a linked administrative and survey dataset. Data on well‐being and other sociodemographic information, such as gender, age, ethnicity, migration history, number of dependent children, and family type, come from New Zealand's General Social Survey. Data on individuals' employment come from The Longitudinal Employment Outcomes dataset. Details can be found in Table 1 in the Appendix.

Data from the General Social Survey cover the period from April of the current year through to March of the subsequent year. The General Social Survey began in 2008 and data are collected every two years.^6^ The sample is representative of people in New Zealand aged 15 years and older. Approximately 8,000 individuals are surveyed in each wave.

The well‐being indicators in this study were selected using the following criteria: (i) consistent measurement across time to make temporal comparisons possible; and (ii) the responses needed to be sensitive to change across time in a way that suggests they could be responsive to shocks (for example, having enough money to meet needs), rather than indicators that may represent broader longer‐term determinants (such as general health). In conclusion, we chose four well‐being indicators which satisfied the above conditions:

- To measure **life satisfaction**, respondents were asked: ‘How do you feel about your life as a whole?’. A ‘1’ to ‘5’ scale was employed for measuring, with ‘1’ being very/completely dissatisfied and ‘5’ being very/completely satisfied.^7^
- To measure **general health**, respondents were asked: ‘In general, would you say your health is excellent, very good, good, fair, or poor?’. Response options ranged from ‘1’ to ‘5’, with ‘1’ being poor and ‘5’ being excellent.^8^
- To measure **income adequacy**, respondents were asked: ‘How well does [your household] income meet your everyday needs, for such things as accommodation, food, clothing and other necessities?’. Response options ranged from ‘0’ to ‘3’, with ‘0’ being not enough money, ‘1’ being only just enough money, ‘2’ being enough money, and ‘3’ being more than enough money.
- To measure **safety**, respondents were asked: ‘How safe or unsafe do you feel walking alone in your neighbourhood after dark?’. A ‘1’ to ‘5’ scale was employed for measuring, with ‘1’ being very unsafe and ‘5’ being very safe. This measure was consistent across years.

Table 1 presents an overview of well‐being indicators across six waves of New Zealand's General Social Survey, spanning 2008–18. For a summary of other independent variables, see Table 2 in the Appendix.

**TABLE 1:** Descriptions of well‐being indicators.

Variables	Level	*Affected meshblocks in CHC only (Model 1)*		*Affected meshblocks in affected city/districts (Model 2)*		*Affected city/districts (Model 3)*	
		Observations	%	Observations	%	Observations	%
Life satisfaction	Very dissatisfied (1)	45,348	2.1	45,600	2.1	49,608	2.1
	Dissatisfied (2)	–	4.1	–	4.1	–	4.1
	No feeling either way (3)	–	7.9	–	7.8	–	7.9
	Satisfied (4)	–	53.9	–	54.0	–	53.9
	Very satisfied (5)	–	32.0	–	32.0	–	32.0
General health	Poor (1)	45,384	3.8	45,636	3.8	49,647	3.8
	Fair (2)	–	12.0	–	12.0	–	12.0
	Good (3)	–	28.5	–	28.5	–	28.6
	Very good (4)	–	36.4	–	36.4	–	36.3
	Excellent (5)	–	19.3	–	19.3	–	19.3
Feeling safe	Very unsafe (1)	42,054	8.6	42,294	8.6	46,083	8.5
	Moderate unsafe (2)	–	16.1	–	16.1	–	16.1
	Safe (3)	–	19.8	–	19.8	–	19.9
	Moderate safe (4)	–	40.2	–	40.2	–	40.3
	Very safe (5)	–	15.3	–	15.3	–	15.2
Enough money	Not enough money (0)	44,427	14.1	44,676	14.0	48,606	13.9
	Only just enough money (1)	–	29.8	–	29.8	–	29.6
	Enough money (2)	–	40.6	–	40.7	–	40.8
	More than enough money (3)	–	15.5	–	15.5	–	15.7

**Source:** authors.

### 3.3 Treatment and control groups

In this study, we estimate three different groupings of control and treatment. *Model 1* assumed that individuals in the affected meshblocks in CHC only were exposed to the earthquake, while the control group is composed of individuals from the rest of New Zealand (excluding all of the unaffected meshblocks in CHC and other districts in Canterbury Region). The *Model 2* treatment group includes all individuals in affected meshblocks in all three affected city/districts—CHC, Waimakariri, and Selwyn—while the control group is the same as in Model 1. For *Model 3*, the treatment is defined as all individuals residing in the three affected city/districts, while the control group is the same as that for Models 1 and 2. We always exclude Canterbury Region from the control group to ensure that the effects of the earthquakes on the treatment group can be accurately assessed without interference by external factors because of spillover effects.

Table 2 details the total number of observations in the treatment and control group in the three models. The control group is the same in all three models, while the size of the treated groups differs. The total number of observations of treatment in Models 1 and 2 accounts for only 4.3 and 4.9 per cent, respectively, as compared to the control groups, while it is larger (14.1 per cent) for Model 3. To avoid issues related to migration, we define the treatment and control groups based on the location of individuals before the earthquakes. The idea is that people who moved out of Canterbury because of the CES, as captured in surveys from 2012–18, still belong to the treatment groups as they were affected by the disaster. We can identify the location of 95 per cent of individuals based on the location data in the IDI. After defining the location of individuals before the earthquakes, we find that six per cent of individuals in Canterbury moved out of Canterbury Region after the CES. Most of these people migrated to Wellington, Auckland, and Otago (12, 17, and 18 per cent, respectively)—see Table 3 in the Appendix.

**TABLE 2:** Total individuals in treatment and control group by survey years.

Year	N in treatment group			N in control group
	*Affected meshblocks in CHC only (Model 1)*	*Affected meshblocks in affected city/districts (Model 2)*	*Affected city/districts (Model 3)*	
**2008**	279	318	1,017	7,329
**2010**	246	285	924	7,254
**2012**	315	366	1,026	7,071
**2014**	345	381	1,122	7,278
**2016**	345	384	1,032	7,128
**2018**	354	405	1,026	7,467
**Total**	**1,884**	**2,139**	**6,147**	**43,527**

**Note:** N is total individuals from New Zealand's General Social Survey.

**Source:** authors.

## 4 METHODOLOGY

### 4.1 The econometric model and estimation

The regression equation to estimate the determinants of well‐being is:

$$\log\left(\frac{P\left(Y_{\mathrm{it}}=1\right)}{1-P\left(Y_{\mathrm{it}}=1\right)}\right)=\beta_{0}+\beta_{1}{\text{Treatment}}_{i}+\alpha_{1}{\text{post}}_{t}+\alpha_{2}{\text{Treatment}}_{i}*{\text{post}}_{t}+\mu_{1}X_{\mathrm{it}}+\rho_{1}{\text{Treatment}}_{i}*Z_{\mathrm{it}}+\rho_{2}{\text{post}}_{t}*Z_{\mathrm{it}}+\rho_{3}{\text{Treatment}}_{i}*{\text{post}}_{t}*Z_{\mathrm{it}}+z_{t}+\varepsilon_{\mathrm{it}}$$

$Y_{\mathrm{it}}$ is the dependent variables of well‐being relating to the life satisfaction, general health, safety, and income adequacy of individual *i* in survey year *t*. ${\text{Treatment}}_{i}$ is treatment as defined for the three different models: affected meshblocks in CHC only, affected meshblocks in the three affected city/districts, and the three affected city/districts. ${\text{post}}_{t}$ is any survey year after the earthquakes. $\alpha_{2}$ denotes the coefficients of the well‐being of individuals in terms of whether or not they were living in an earthquake‐affected area after the disaster. $X_{\mathrm{it}}$ is a vector of socioeconomic characteristics, including age during the CES, gender, ethnicity, migrant to New Zealand, dependent children, family type, and employment during the CES. $Z_{\mathrm{it}}$ is a vector of the key socioeconomic characteristics on which we focus in interaction terms with ${\text{Treatment}}_{i}$ and ${\text{post}}_{t}$, including age during the CES (25–35 years old), gender, ethnicity (Māori), migrant to New Zealand, sole individuals with dependent children, and employment during the CES. $\rho_{3}$ denotes coefficients of individuals' well‐being vis‐à‐vis whether or not they belong to one of the groups (female, Māori, sole individuals with dependent children, migrant, age 25–35 during the CES, and employed during the earthquakes) and living in an earthquake‐affected area after the disaster. $z_{t}$ is the (survey) year fixed effect. $\varepsilon_{\mathrm{it}}$ is the error term. The standard errors are clustered at the city/district level to adjust for heteroskedasticity and correlation over time.

The well‐being indicators are originally based on a Likert scale from ‘1’ to ‘5’, so the specification could be estimated with an ordered logit; however, the ordered logit regression requires the proportional odds (PO) assumption to hold. We find that the PO assumption is rejected, and a generalised ordered logit (GOL) is difficult to interpret in a DDD set up. Therefore, we simplified the dependent variables (the well‐being indicators) into binary measures for all four elements: life satisfaction (‘0’ = dissatisfied, very dissatisfied, no feeling; ‘1’ = satisfied, very satisfied); general health (‘0’ = poor, fair; ‘1’ = good, excellent, very excellent); income adequacy (‘0’ = not enough money; ‘1’ = just enough, enough, more than enough money); and safety (‘0’ = very unsafe, unsafe; ‘1’ = safe, moderate safe, very safe).

Although converting these well‐being indicators into binary measures reduces their information content, the results are not much different qualitatively to those from the ordered logit regressions. The results for the latter can be seen in Tables 16–27 in the Appendix.

To interpret the results, we calculate the odds ratio (OR) by exponentiating each side of all three models. An OR of less than one indicates that the odds of choosing zero responses will increase when the predictor rises by one unit, whereas an OR of more than one signifies the opposite. If the OR is equal to one, there is no difference between the two levels of response when the predictors change.

## 5 RESULTS

In this section, Tables 3, 4, 5, 6 focus only on the important DiD and interaction—the full results of the estimated models can be seen in Tables 4–15 in the Appendix.

**TABLE 3:** Impact of the earthquakes on life satisfaction.

Variables	Life satisfaction								
	Affected meshblocks in CHC			Affected meshblocks in affected city/districts			Affected city/districts		
	(1)	(2)	(3)	(4)	(5)	(6)	(7)	(8)	(9)
treatment#post_EQ	0.813***	0.829***	0.706***	0.789***	0.797***	0.687***	0.908*	0.897**	0.847*
	(0.0312)	(0.0293)	(0.0563)	(0.0467)	(0.0487)	(0.0743)	(0.0481)	(0.0416)	(0.0736)
treatment#post_EQ#									
Female			1.07			0.997			0.94
			(0.0574)			(0.0912)			(0.0971)
Māori			0.487***			0.525***			0.812**
			(0.0441)			(0.0689)			(0.0813)
Sole individuals with dependent children			0.455***			0.611			1.117
			(0.0516)			(0.196)			(0.2)
Age (25–35)			1.209**			1.235**			1.077
			(0.106)			(0.116)			(0.109)
Migrant			1.176***			1.031			1.166*
			(0.0711)			(0.189)			(0.0985)
Employed during the earthquakes			1.228***			1.275***			1.064
			(0.0781)			(0.116)			(0.0945)
Constant	5.375***	3.744***	3.432***	5.382***	3.774***	3.441***	5.341***	3.898***	3.581***
	(0.271)	(0.376)	(0.373)	(0.271)	(0.377)	(0.373)	(0.253)	(0.388)	(0.404)
Control: socio‐characteristics	No	Yes	Yes	No	Yes	Yes	No	Yes	Yes
**Observations**	**45,345**	**45,312**	**45,312**	**45,600**	**45,564**	**45,564**	**49,608**	**49,572**	**49,572**

**Note:** all models are with survey year fixed effects. Control variables include age during the CES, gender, ethnicity, migrant to New Zealand, dependent children, family type, and employed during the earthquakes. Cluster standard errors (at the territorial authority level) are in parentheses. * *p* < 0.1, ** *p* < 0.05, *** *p* < 0.01.

**Source:** authors.

**TABLE 4:** Impact of the earthquakes on general health.

Variables	General health								
	Affected meshblocks in CHC			Affected meshblocks in affected city/districts			Affected city/districts		
	(1)	(2)	(3)	(4)	(5)	(6)	(7)	(8)	(9)
treatment#post_EQ	0.797***	0.813***	0.872	0.871	0.882	0.902	1.01	0.988	0.962
	(0.0437)	(0.0347)	(0.0817)	(0.112)	(0.106)	(0.11)	(0.061)	(0.0506)	(0.0981)
treatment#post_EQ#									
Female			0.888**			0.979			0.937
			(0.0438)			(0.105)			(0.0817)
Māori			0.146***			0.478			1.191
			(0.0993)			(0.385)			(0.225)
Sole individuals with dependent children			2.171***			1.671*			0.897
			(0.231)			(0.506)			(0.198)
Age (25–35)			0.936			0.93			0.98
			(0.0872)			(0.137)			(0.156)
Migrant			1.007			0.813			1.180*
			(0.0749)			(0.25)			(0.1)
Employed during the earthquakes			1.074			1.071			1.024
			(0.0783)			(0.0794)			(0.094)
Constant	5.437***	9.510***	8.555***	5.420***	9.477***	8.489***	5.299***	9.102***	8.159***
	(0.387)	(1.456)	(1.369)	(0.384)	(1.444)	(1.356)	(0.363)	(1.36)	(1.312)
Control: socio‐characteristics	No	Yes	Yes	No	Yes	Yes	No	Yes	Yes
**Observations**	**45,384**	**45,348**	**45,399**	**45,636**	**45,600**	**45,600**	**49,647**	**49,611**	**49,611**

**Notes:** all models are with survey year fixed effects. Control variables include age during the CES, gender, ethnicity, migrant to New Zealand, dependent children, family type, and employed during the earthquakes. Cluster standard errors (at the territorial authority level) are in parentheses. * *p* < 0.1, ** *p* < 0.05, *** *p* < 0.01.

**Source:** authors.

**TABLE 5:** Impact of the earthquakes on income adequacy.

Variables	Income adequacy								
	Affected meshblocks in CHC			Affected meshblocks in affected city/districts			Affected city/districts		
	(1)	(2)	(3)	(4)	(5)	(6)	(7)	(8)	(9)
treatment#post_EQ	0.829**	0.832***	0.985	0.938	0.931	0.863	1.098	1.079	1.097
	(0.0651)	(0.0401)	(0.0841)	(0.145)	(0.122)	(0.143)	(0.106)	(0.0828)	(0.122)
treatment#post_EQ#									
Female			0.728***			0.895			0.917
			(0.039)			(0.198)			(0.0845)
Māori			1.353***			1.774**			1.293
			(0.112)			(0.482)			(0.233)
Sole individuals with dependent children			0.851			0.728*			1.591***
			(0.0927)			(0.132)			(0.178)
Age (25–35)			0.633***			0.680***			0.377***
			(0.0479)			(0.0847)			(0.0663)
Migrant			0.574***			0.551***			1.084
			(0.0496)			(0.0593)			(0.118)
Employed during the earthquakes			1.254***			1.409**			1.097
			(0.0945)			(0.199)			(0.119)
Constant	4.982***	4.644***	4.357***	5.018***	4.701***	4.395***	4.970***	4.592***	4.260***
	(0.35)	(0.443)	(0.445)	(0.356)	(0.452)	(0.451)	(0.339)	(0.396)	(0.411)
Control: socio‐characteristics	No	Yes	Yes	No	Yes	Yes	No	Yes	Yes
**Observations**	**44,427**	**44,403**	**44,451**	**44,676**	**44,649**	**44,649**	**48,606**	**48,579**	**48,579**

**Notes:** all models are with survey year fixed effects. Control variables include age during the CES, gender, ethnicity, migrant to New Zealand, dependent children, family type, and employed during the earthquakes. Cluster standard errors (at the territorial authority level) are in parentheses. * *p* < 0.1, ** *p* < 0.05, *** *p* < 0.01.

**Source:** authors.

**TABLE 6:** Impact of the earthquakes on safety.

Variables	Safety								
	Affected meshblocks in CHC			Affected meshblocks in affected city/districts			Affected city/districts		
	(1)	(2)	(3)	(4)	(5)	(6)	(7)	(8)	(9)
treatment#post_EQ	0.880***	0.911*	1.075	0.872**	0.888*	0.969	0.993	1.035	0.781
	(0.0434)	(0.0473)	(0.0975)	(0.0476)	(0.0553)	(0.166)	(0.0629)	(0.066)	(0.128)
treatment#post_EQ#									
Female			0.859*			1.514***			1.186
			(0.0669)			(0.132)			(0.14)
Māori			1.222*			1.141			0.693***
			(0.126)			(0.174)			(0.0813)
Sole individuals with dependent children			1.469***			1.418**			2.212***
			(0.141)			(0.198)			(0.255)
Age (25–35)			0.91			1.102			1.131
			(0.0629)			(0.314)			(0.157)
Migrant			1.452***			1.187			1.011
			(0.0761)			(0.285)			(0.201)
Employed during the earthquakes			0.489***			0.553***			1.175
			(0.0323)			(0.0982)			(0.144)
Constant	3.912***	11.90***	10.73***	3.918***	11.85***	10.68***	3.922***	11.85***	10.58***
	(0.281)	(1.499)	(1.431)	(0.282)	(1.486)	(1.42)	(0.274)	(1.425)	(1.366)
Control: socio‐characteristics	No	Yes	Yes	No	Yes	Yes	No	Yes	Yes
**Observations**	**42,054**	**42,036**	**42,081**	**42,294**	**42,276**	**42,276**	**46,083**	**46,065**	**46,065**

**Notes:** all models are with survey year fixed effect. Control variables include age during the CES, gender, ethnicity, migrant to New Zealand, dependent children, family type, and employed during the earthquakes. Cluster standard errors (at the territorial authority level) are in parentheses. * *p* < 0.1, ** *p* < 0.05, *** *p* < 0.01.

**Source:** authors.

Each table has nine columns considering the different treatment groups as described in the three models above: columns (1)–(3) for Model 1, columns (4)–(6) for Model 2, and columns (7)–(9) for Model 3. Furthermore, the columns vary by the inclusion of sociodemographic control variables. The differences among these models lie in the control variables and the three‐way interaction terms. Columns (1), (4), and (7) represent the models without socio‐characteristic control variables; columns (2), (5), and (8) include these control variables; columns (3), (6), and (9) add the three‐way interaction terms (the DDD model). Note that we only show these coefficients in these tables; the full models in Tables 4–15 in the Appendix provide additional details. In the tables in the Appendix, we include models with each three‐way interaction term for each of the six subgroups (gender, ethnicity, sole individuals with dependent children, age 25–35 during the CES, migrant, and employed during the earthquakes) as well as models that include all six subgroups. As can be seen, however, the results when adding all subgroups in one model and all single subgroups in three‐way interaction terms are not very different. Therefore, we describe only the model with three‐way interaction terms for all subgroups here.

For the three‐way interaction terms, if the OR is more than one, it can be interpreted that individuals belonging to a subgroup in the affected area after the earthquakes are more likely to increase their well‐being. In contrast, if this OR is less than one, individuals in the subgroup in the affected area after the earthquakes are less likely to increase their well‐being.

### 5.1 Life satisfaction

Table 3 presents the OR results from DDD models examining the impact of the CES on life satisfaction. Across all models, the interaction term consistently shows a significant reduction in life satisfaction post earthquakes for individuals residing in the affected areas at the time of the disaster. For individuals in affected meshblocks in CHC (columns (1)–(3)), ORs range significantly from 0.706 to 0.829, indicating a substantial decrease in life satisfaction. For individuals in affected meshblocks in the three most affected city/districts (columns (4)–(6)), ORs range significantly from 0.687 to 0.797, indicating a negative impact of the earthquakes on life satisfaction (and largely overlapping with the range of impacts estimated for Model 1). When considering treatment for all individuals in the three affected city/districts (columns (7)–(9)), ORs range from 0.847 to 0.908, indicating a weaker effect, probably owing to the inclusion of less severely impacted regions in the treatment group.

In terms of the three‐way interaction term, we find that Māori individuals in affected areas experience a significantly greater reduction in life satisfaction post earthquakes, with ORs of 0.487, 0.525, and 0.812 across the models. Sole individuals with dependent children in affected meshblocks in CHC also show more significant reductions in life satisfaction post earthquakes (OR = 0.455). The younger age group (25–35) in affected meshblocks in the affected city/districts during the time of the two earthquakes shows increased life satisfaction post earthquakes (ORs = 1.209 and 1.235). The same is true for migrants in affected meshblocks in CHC (OR = 1.176), and those employed during the earthquakes (ORs = 1.228 and 1.275).

The consistent negative impact for the primary interaction DiD term indicates a substantial decline in life satisfaction for those in affected areas. Māori and sole individuals with dependent children also show a larger decrease in life satisfaction in the wake of the CES. Conversely, age, migrants, and employed individuals showed higher life satisfaction.

### 5.2 General health

Table 4 shows the OR results from DDD models examining the impact of the CES on subjective general health. As can be seen from the DiD interaction term, for individuals in affected meshblocks in CHC (columns (1)–(3)), the interaction term is significant and less than one (0.797 to 0.872), indicating a decrease in general health post earthquakes. For individuals in affected meshblocks in the three most affected city/districts (columns (4)–(6)), the interaction term is also less than one but statistically insignificant. For all individuals in the three affected city/districts (columns (7)–(9)), the interaction term is closer to one and again not significant, indicating little to no change in general health post earthquakes.

Regarding the three‐way DDD interaction term in columns (3), (6), and (9), the results are varied. For gender (female), the ORs are significantly less than one in column (3) for treatment‐affected meshblocks in CHC (OR = 0.888), suggesting that females in these areas post earthquakes experienced a relatively lower likelihood of reporting good general health as compared to males or females there before the earthquakes or females in New Zealand afterwards. For Māori, the ORs are significantly less than one in column (3) (OR = 0.146), indicating a substantial reduction in general health for Māori individuals post earthquakes. Sole individuals with dependent children in affected meshblocks in CHC and the three affected city/districts show a significant increase in general health post earthquakes (ORs = 2.171 and 1.671, respectively), as compared to sole individuals with dependent children in the same affected area before the earthquakes or sole individuals with dependent children in all of New Zealand post earthquakes. This is surprising, since this population is typically found to be more vulnerable to various shocks. The young (aged 25–35) and individuals employed during the CES in the affected area show no significant change in general health post earthquakes (unlike the results for life satisfaction). Migrants in affected city/districts show a significant increase in general health post earthquakes in column (9) (OR = 1.180).

In conclusion, the CES had varied impacts on general health across different subgroups and geographic definitions of affected areas. While certain subgroups, such as females and Māori individuals, experienced declines in general health, others, such as sole individuals with dependent children and migrants, exhibited resilience and improvements in health outcomes post earthquakes.

### 5.3 Income adequacy

Table 5 shows the OR results assessing the impact of the CES on income adequacy, specifically whether individuals perceive that they have enough money to meet everyday needs. The DiD interaction term reflects changes in economic well‐being for individuals in the affected areas post earthquakes. For individuals in affected meshblocks in CHC (columns (1)–(3)), the interaction term is significant and less than one in columns (1) and (2) (OR = 0.829 and 0.832, respectively), indicating a decline in subjective income adequacy after the earthquakes. For individuals in affected meshblocks across the three most affected city/districts (columns (4)–(6)) and all individuals in the affected city/districts (columns (7)–(9)), the interaction term is not significant, suggesting no change in subjective income adequacy post earthquakes when the treatment group is more broadly defined.

The three‐way DDD interaction term presents more varied results. For females, the OR in column (3) is significantly less than one (0.728), highlighting a notable decrease in income adequacy for females in affected meshblocks in CHC post earthquakes. For Māori, the ORs are significantly greater than one in columns (3) and (6) (1.353 and 1.774, respectively), indicating an increase in perceived income adequacy for Māori individuals post earthquakes. Sole individuals with dependent children experienced a significant decrease in this measure post earthquakes in column (6) (OR = 0.728) but a significant increase in column (9) (OR = 1.591), showing mixed impacts. The young age group (25–35 years) shows significant reductions in income adequacy post earthquakes across all models (ORs range from 0.377 to 0.680). Migrants show significant reductions in income adequacy post earthquakes in columns (3) and (6) (ORs = 0.574 and 0.551, respectively). Employed individuals during the earthquakes show significant increases in economic well‐being afterwards in columns (3) and (6) (ORs = 1.254 and 1.409, respectively), consistent with our previous results.

In summary, we find a decline in perceived income adequacy for individuals in affected meshblocks in CHC post earthquakes. Females, the young, and migrants generally experienced reductions in this measure, while Māori and employed individuals showed resilience (an increase). The mixed results for sole individuals with dependent children suggest that this group had both positive and negative experiences post earthquakes.

### 5.4 Safety

Table 6 presents the OR results from DDD models examining the impact of the CES on (subjective) safety. Focusing on the DiD interaction term, for individuals in affected meshblocks in CHC (columns (1)–(3)), the interaction term is significant and less than one in columns (1) and (2) (ORs = 0.880 and 0.911, respectively), indicating a significant decrease in perceived safety post earthquakes. For individuals in affected meshblocks in the three most affected city/districts (columns (4)–(6)), the interaction term is also less than one and significant in columns (4) and (5) (ORs = 0.872 and 0.888, respectively), again suggesting a decrease in perceived safety post earthquakes. For all individuals in the three affected city/districts (columns (7)–(9)), the interaction term is not significant, indicating little to no change in perceived safety post earthquakes when the threshold for determining the treatment group become broader.

Regarding the three‐way DDD interaction term in columns (3), (6), and (9), the results are varied. For females, the OR is significantly less than one in column (3) (OR = 0.859), suggesting that females in affected meshblocks in CHC were less likely to feel safe post earthquakes as compared to males in the same areas, or to females elsewhere in New Zealand post earthquakes, or to females in the affected area pre earthquakes. However, the OR in column (6) is significantly greater than one (OR = 1.514), suggesting that the feeling of safety post earthquakes varies considerably depending on the location (affected meshblocks in CHC versus affected city/districts). For Māori, the ORs are more than one in column (3) (OR = 1.222) and significantly less than one in column (9) (OR = 0.693), indicating again mixed impacts on perceived safety post earthquakes across the different affected areas. Sole individuals with dependent children in affected meshblocks in CHC and in the three affected city/districts show a significant increase in perceived safety post earthquakes (ORs = 1.469, 1.418, and 2.212, respectively). Migrants in affected meshblocks in CHC show a significant increase in perceived safety post earthquakes in column (3) (OR = 1.452). Individuals employed during the earthquakes show a significant decrease in perceived safety post earthquakes in affected meshblocks in CHC and in the three affected city/districts (ORs = 0.489 and 0.553, respectively).

In conclusion, the findings for perceived safety suggest that while some groups, such as sole individuals with dependent children and migrants, experienced increased safety, others, particularly females, experienced significant decreases in their (subjective) safety.

## 6 ROBUSTNESS CHECK

To probe the robustness of our results, we ran the GOL regression to acquire more information on the transition between different levels of the well‐being indicators (see Tables 28–39 in the Appendix). The GOL regression model compares all categories greater than the current category with those that are less than or equal to the current category. Therefore, positive coefficients indicate that higher values of the explanatory variable correspond to higher levels of the outcome variable category. Conversely, negative coefficients suggest that higher values of the explanatory variable increase the likelihood of being in the current or a lower category (Williams, 2006).

For the GOL models of the four well‐being indicators in meshblocks affected in CHC, the results show significant values of less than one for all of the relevant comparisons. This indicates that individuals are less likely to report increased well‐being, confirming the robustness of our results for the most affected meshblocks (those in CHC). Similarly, for the well‐being indicators in affected meshblocks across the three city/districts and the three affected city/districts, the DiD interaction term variable is significantly less than one for life satisfaction; again, this is consistent with our previous findings.

For the three‐way DDD interaction terms, although the results vary across different category comparisons, the sign of the results (whether more than or less than one) remains quite similar when compared to our baseline model estimated on the binary well‐being indicators. As previously mentioned, however, interpreting these results in the context of DDD models is more complex with the GOL model.

## 7 CONCLUSION

This study has examined the impacts of the CES on individual subjective well‐being, focusing on life satisfaction, general health, income adequacy, and safety, using data from New Zealand's General Social Survey and a DDD approach. By matching this data with weather‐related insurance claims from the Earthquake Commission to identify the affected (treated) meshblocks, we were able to investigate the impacts of the earthquakes at a micro level.

Based on a comprehensive analysis of the effects of the CES on various dimensions of well‐being, it is evident that they were nuanced and varied. Yet, the findings consistently demonstrate a significant decline in life satisfaction for individuals in the affected area post earthquakes across all models and treatment area definitions, highlighting the long‐term sensitivity of life satisfaction to catastrophic shocks. Regarding severity, more narrowly‐defined affected areas, such as severely affected meshblocks in CHC, show comprehensive negative well‐being impacts across all four well‐being indicators.

The findings illustrate that vulnerable groups, such as Māori and sole individuals with dependent children in CHC, experienced significant declines in life satisfaction post earthquakes. Conversely, certain demographic groups, including the younger age group, migrants, and employed individuals during the earthquakes, exhibited more resilience, with either stable or improved life satisfaction outcomes.

Similarly, general health outcomes showed mixed results across different areas. Vulnerable groups like Māori experienced significant declines post earthquakes, contrasting with the resilience observed among sole individuals with dependent children, who showed improved health outcomes in CHC and the most affected city/districts. Economic well‐being declined notably in CHC after the CES, with females and the young experiencing reductions, while Māori and employed individuals demonstrated increases. Meanwhile, perceptions of safety decreased significantly in CHC and the most affected city/districts, highlighting heightened vulnerabilities among females and the employed, while the opposite was true for sole individuals with dependent children and migrants.

These findings first and foremost expose a hidden adverse impact of the earthquakes, and underscore the importance of targeted interventions and support mechanisms tailored to the specific needs of vulnerable populations as part of disaster reconstruction and community rehabilitation efforts. Addressing these disparities requires a nuanced approach that considers socioeconomic factors, community resilience, and cultural contexts, in order to mitigate effectively the impacts of disasters on the affected population's well‐being.

## FUNDING

Resilience to Nature's Challenges' National Science Challenge.

## Supporting information


**Data S1** Supporting Information

## Data Availability

Research data are not shared.

## Endnotes

^1^
For more information, see https://www.treasury.govt.nz/information-and-services/nz-economy/higher-living-standards/our-living-standards-framework (last accessed on 5 June 2025).

^2^
All supplementary materials can be found online in the supporting information section at the end of the article.

^3^
We note that before the CES, the earthquake‐affected region was classified as ‘low risk’ by the New Zealand Government (a classification that determined construction standards).

^4^
In 2024, the EQC changed its name to the Natural Hazards Commission. When we completed this project, the organisation was still called the EQC, so we kept that name in our study.

^5^
A meshblock is the smallest geographic unit available for New Zealand. Each meshblock contains between 40 and 60 houses. We also removed claims whose status was reported as ‘open’, ‘reopen’, ‘declined’, ‘not accepted’, ‘withdrawn’, ‘invalid’, ‘fieldwork in progress’, ‘fieldwork complete’, and ‘accepted’.

^6^
The General Social Survey had to delay its 2020 survey owing to the COVID‐19 (coronavirus disease 2019) pandemic; it conducted it in 2021 with a smaller sample than usual. We did not use the 2021 survey, as the impact of COVID‐19 lockdowns from March 2020 most likely affected well‐being dramatically.

^7^
This measurement changed to a 0–10 scale from 2014–20. The 0–10 scale was collapsed to a 1–5 scale to harmonise across all years. The collapsing method relies on a similar distribution between the two scales, 0–10 and 1–5, as illustrated in Figure 2 in the Appendix.

^8^
We could not include a measure of mental health due to large differences in measurements across time.
