# Supplementary material for: Impact of Canterbury earthquakes on well‐being in New Zealand
Source: Disasters. 2025 Jun 26;49(4):e12692. doi: 10.1111/disa.12692 (PMC12199772; doi:10.1111/disa.12692)
Supplement: Supplementary file 1 — Data S1 Supporting Information [file DISA-49-e12692-s001.docx]

**Appendix**

## Figure 1: The Living Standards Framework Dashboard ([Treasury, 2018](#_ENREF_26))

**
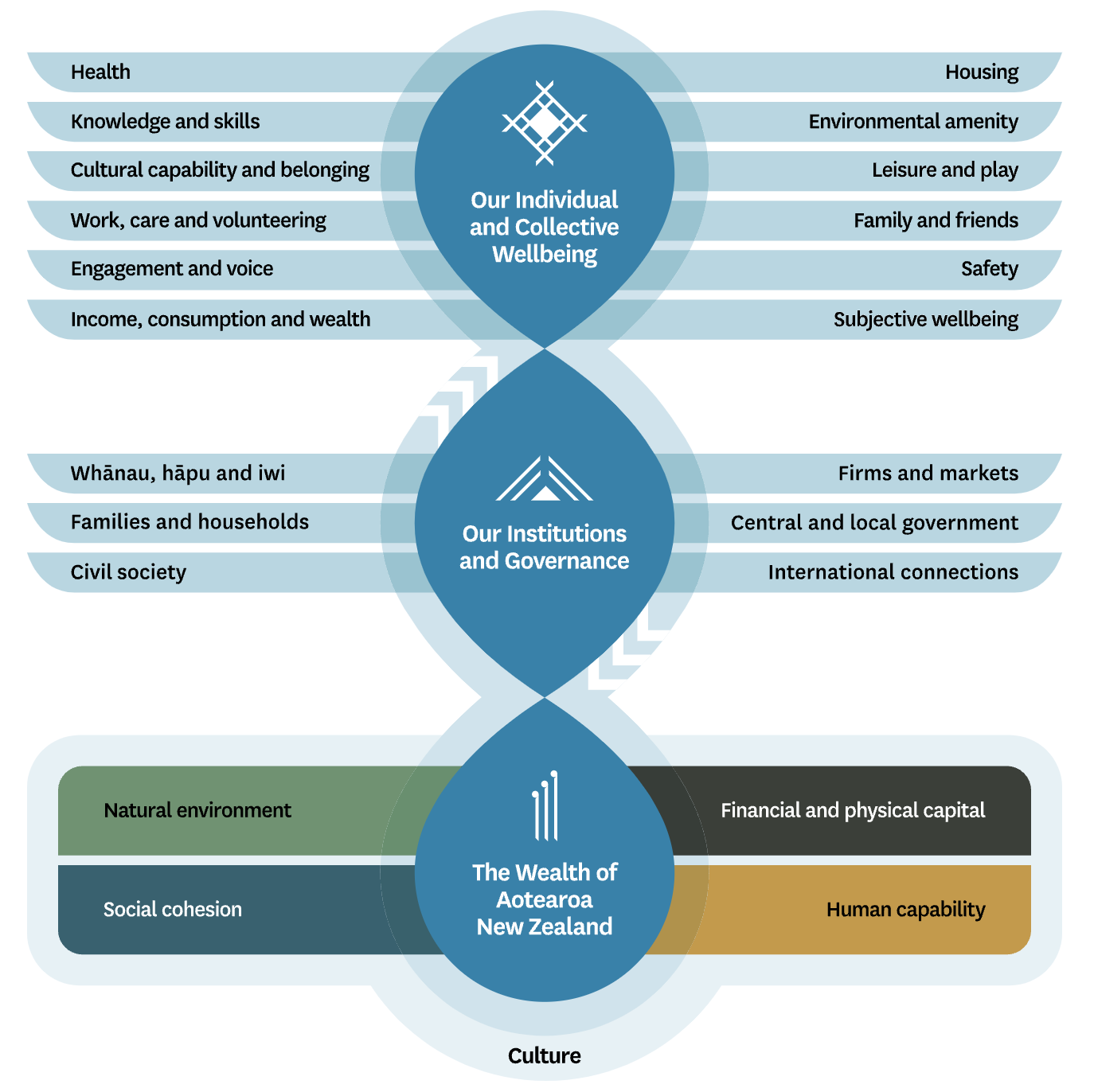
**

**Figure 2: Distribution of Life Satisfaction with 0-10 scale and 1-5 scale**

| ****** |
| --- |
| ****** |

**Table 1: Independent variables and sources**

| Independent variables | Sources |
| --- | --- |
| Age during the Earthquake | GSS |
| Gender (1=Female) | GSS |
| Migrant to NZ | GSS |
| Ethnicity | GSS |
| Number of dependent children | GSS |
| Type of family | GSS |
| Employed during the earthquake | LEED |

**Table 2: Descriptive table for control variables**

|  |  | *Affected meshblocks in CHC district only* ***(Model 1)*** | | | *Affected meshblocks in affected districts* ***(Model 2)*** | | | | *Affected districts*  ***(Model 3)*** | | | |
| --- | --- | --- | --- | --- | --- | --- | --- | --- | --- | --- | --- | --- |
| **Variables** | **Level** | **Obs** | **%** | | **Obs** | | **%** | | **Obs** | | **%** | |
| Gender | Male | 45,408 | | 44.6 | | 45,663 | | 55.4 | | 49,674 | | 44.9 |
|  | Female |  | | 55.4 | |  | | 44.6 | |  | | 55.1 |
| Age during the EQ | Under 25 years old | 45,411 | | 16.5 | | 45,663 | | 16.5 | | 49,677 | | 16.6 |
|  | 26-35 years old |  | | 15.6 | |  | | 15.5 | |  | | 15.5 |
|  | 36-55 years old |  | | 35.5 | |  | | 35.5 | |  | | 35.5 |
|  | 56-65 years old |  | | 15.5 | |  | | 15.5 | |  | | 15.5 |
|  | over 65 years old |  | | 16.9 | |  | | 17.0 | |  | | 16.9 |
| Migrant to NZ | Yes | 45,375 | | 25.6 | | 45,627 | | 25.5 | | 49,638 | | 25.3 |
|  | No |  | | 74.4 | |  | | 74.5 | |  | | 74.7 |
| European | Yes | 45,408 | | 74.1 | | 45,663 | | 74.2 | | 49,677 | | 74.9 |
|  | No |  | | 25.9 | |  | | 25.8 | |  | | 25.1 |
| Māori | Yes | 45,408 | | 13.7 | | 45,663 | | 13.7 | | 49,677 | | 13.1 |
|  | No |  | | 86.3 | |  | | 86.3 | |  | | 86.9 |
| Pacific | Yes | 45,408 | | 5.4 | | 45,663 | | 5.4 | | 49,677 | | 5.1 |
|  | No |  | | 94.6 | |  | | 94.6 | |  | | 94.9 |
| Asian | Yes | 45,408 | | 8.1 | | 45,663 | | 8.0 | | 49,677 | | 7.9 |
|  | No |  | | 91.9 | |  | | 92.0 | |  | | 92.1 |
| Melaa | Yes | 45,408 | | 0.7 | | 45,663 | | 0.7 | | 49,677 | | 0.7 |
|  | No |  | | 99.3 | |  | | 99.3 | |  | | 99.3 |
| Others | Yes | 45,408 | | 1.4 | | 45,663 | | 1.4 | | 49,677 | | 1.4 |
|  | No |  | | 98.6 | |  | | 98.6 | |  | | 98.6 |
| Dependent children | Yes | 45,408 | | 33.3 | | 45,663 | | 33.3 | | 49,677 | | 33.2 |
|  | No |  | | 66.7 | |  | | 66.7 | |  | | 66.8 |
| Employed during the EQ | Yes | 45,408 | | 74.2 | | 45,663 | | 74.2 | | 49,677 | | 74.3 |
|  | No |  | | 25.8 | |  | | 25.8 | |  | | 25.7 |
| Family type | Couples with dependent children | 45,408 | | 21.6 | | 45,663 | | 21.6 | | 49,677 | | 2.6 |
|  | Couples without dependent children |  | | 32.6 | |  | | 32.6 | |  | | 32.8 |
|  | Sole parents with dependent children |  | | 6.6 | |  | | 6.6 | |  | | 6.5 |
|  | Single people without dependent children |  | | 39.2 | |  | | 39.2 | |  | | 39.1 |

**Table 3: Total observation before and after defining location before earthquakes**

| Region | N in survey  (1) | N after defining pre-location (2) | Difference  (1)-(2) |
| --- | --- | --- | --- |
| Northland Region | 2.151 | 2.139 | 12 |
| Auckland Region | 12.516 | 12.444 | 72 |
| Waikato Region | 4.878 | 4.848 | 30 |
| Bay of Plenty Region | 4.011 | 3.984 | 27 |
| Gisborne Region | 909 | 909 | 0 |
| Hawke's Bay Region | 1.812 | 1.803 | 9 |
| Taranaki Region | 1.377 | 1.368 | 9 |
| Manawatu-Wanganui | 2.946 | 2.925 | 21 |
| Wellington Region | 6.693 | 6.645 | 48 |
| West Coast Region | 231 | 225 | 6 |
| Canterbury Region | 7.455 | 7.872 | -417 |
| Otago Region | 2.742 | 2.667 | 75 |
| Southland Region | 1.629 | 1.599 | 30 |
| Tasman Region | 945 | 918 | 27 |
| Nelson Region | 858 | 834 | 24 |
| Marlborough Region | 615 | 594 | 21 |
| Total | 51.774 | 51.774 |  |

**Table 4: Full model - Impact of Earthquake to Life Satisfaction (Model 1)**

| **Variables** | **Life Satisfaction** | | | | | | | | |
| --- | --- | --- | --- | --- | --- | --- | --- | --- | --- |
|  | (1) | (2) | (3) | (4) | (5) | (6) | (7) | (8) | (9) |
| treatment | 0,959 | 0.914*** | 0.877*** | 0.897*** | 0.892*** | 0,952 | 0.929** | 0,924 | 0,922 |
|  | (0.0328) | (0.0277) | (0.0283) | (0.0285) | (0.0298) | (0.032) | (0.0295) | (0.0564) | (0.0664) |
| treatment * post EQ | 0.813*** | 0.829*** | 0.837*** | 0.872*** | 0.878*** | 0.806*** | 0.791*** | 0.732*** | 0.706*** |
|  | (0.0312) | (0.0293) | (0.032) | (0.0359) | (0.0308) | (0.031) | (0.0333) | (0.0475) | (0.0563) |
| Age during the EQ (Ref: <25 y.o) |  |  |  |  |  |  |  |  |  |
| 25-35 years old |  | 0.745*** | 0.746*** | 0.744*** | 0.746*** | 0.730*** | 0.745*** | 0.749*** | 0.741*** |
|  |  | (0.0376) | (0.0376) | (0.0378) | (0.0374) | (0.0444) | (0.0488) | (0.0383) | (0.0436) |
| 36-55 years old |  | 0.593*** | 0.593*** | 0.592*** | 0.595*** | 0.592*** | 0.593*** | 0.597*** | 0.599*** |
|  |  | (0.0242) | (0.0243) | (0.0243) | (0.0242) | (0.0239) | (0.0242) | (0.0248) | (0.0243) |
| 56-65 years old |  | 0.727*** | 0.727*** | 0.726*** | 0.728*** | 0.726*** | 0.727*** | 0.732*** | 0.733*** |
|  |  | (0.0456) | (0.0457) | (0.0457) | (0.0453) | (0.0452) | (0.0455) | (0.0461) | (0.0455) |
| >65 years old |  | 1.230*** | 1.230*** | 1.229*** | 1.231*** | 1.228*** | 1.229*** | 1.237*** | 1.237*** |
|  |  | (0.0787) | (0.0788) | (0.0787) | (0.079) | (0.0778) | (0.0787) | (0.0801) | (0.0795) |
| Household size |  | 1,01 | 1,01 | 1,01 | 1,01 | 1,01 | 1,01 | 1,009 | 1,009 |
|  |  | (0.0162) | (0.0162) | (0.0163) | (0.0162) | (0.0162) | (0.0162) | (0.0162) | (0.0163) |
| Dependent children (1/0) |  | 1.252*** | 1.252*** | 1.251*** | 1.254*** | 1.251*** | 1.252*** | 1.244*** | 1.243*** |
|  |  | (0.089) | (0.089) | (0.0891) | (0.0891) | (0.0887) | (0.0887) | (0.0883) | (0.0881) |
| Gender (1=Female) |  | 1.167*** | 1.123** | 1.167*** | 1.166*** | 1.167*** | 1.167*** | 1.167*** | 1.130** |
|  |  | (0.0317) | (0.053) | (0.0318) | (0.0316) | (0.0317) | (0.0317) | (0.0318) | (0.0571) |
| European |  | 1.195*** | 1.195*** | 1.196*** | 1.195*** | 1.194*** | 1.198*** | 1.194*** | 1.200*** |
|  |  | (0.0561) | (0.0562) | (0.0554) | (0.0558) | (0.0561) | (0.0566) | (0.0563) | (0.0557) |
| Māori |  | 0.861*** | 0.861*** | 0.871* | 0.861*** | 0.861*** | 0.862*** | 0.862*** | 0,89 |
|  |  | (0.046) | (0.046) | (0.0618) | (0.0461) | (0.0461) | (0.0459) | (0.046) | (0.0682) |
| Pacific |  | 0.809*** | 0.808*** | 0.810*** | 0.809*** | 0.808*** | 0.811*** | 0.810*** | 0.813*** |
|  |  | (0.0451) | (0.0453) | (0.0453) | (0.0452) | (0.0452) | (0.0445) | (0.0451) | (0.0447) |
| Asian |  | 1,023 | 1,024 | 1,024 | 1,025 | 1,022 | 1,027 | 1,02 | 1,027 |
|  |  | (0.0688) | (0.0687) | (0.0697) | (0.069) | (0.0687) | (0.0666) | (0.0692) | (0.0676) |
| MELAA |  | 0,877 | 0,878 | 0,878 | 0,878 | 0,875 | 0,882 | 0,875 | 0,88 |
|  |  | (0.154) | (0.154) | (0.154) | (0.155) | (0.153) | (0.152) | (0.154) | (0.154) |
| Other |  | 0,837 | 0,837 | 0,838 | 0,836 | 0,837 | 0,838 | 0,836 | 0,838 |
|  |  | (0.0977) | (0.0978) | (0.0976) | (0.0976) | (0.0977) | (0.0978) | (0.0981) | (0.0979) |
| Migrant to NZ |  | 1.093*** | 1.093*** | 1.094*** | 1.093*** | 1.093*** | 1.117** | 1.092*** | 1.127** |
|  |  | (0.0374) | (0.0374) | (0.0373) | (0.0375) | (0.0373) | (0.0548) | (0.0372) | (0.0592) |
| Sole parents with dependent children |  | 0.615*** | 0.615*** | 0.615*** | 0.562*** | 0.615*** | 0.615*** | 0.619*** | 0.565*** |
|  |  | (0.0538) | (0.0539) | (0.0538) | (0.0579) | (0.0538) | (0.0538) | (0.0539) | (0.0587) |
| Couples with dependent children |  | 1.956*** | 1.955*** | 1.957*** | 1.951*** | 1.955*** | 1.957*** | 1.977*** | 1.974*** |
|  |  | (0.14) | (0.14) | (0.14) | (0.139) | (0.14) | (0.14) | (0.141) | (0.14) |
| Couples without dependent children |  | 2.274*** | 2.273*** | 2.273*** | 2.272*** | 2.274*** | 2.274*** | 2.277*** | 2.275*** |
|  |  | (0.0874) | (0.0875) | (0.0873) | (0.0874) | (0.0874) | (0.0874) | (0.0878) | (0.0879) |
| Employed during the EQ (1/0) |  | 0,994 | 0,994 | 0,994 | 0,994 | 0,994 | 0,994 | 1.111* | 1.121* |
|  |  | (0.0354) | (0.0356) | (0.0354) | (0.0354) | (0.0354) | (0.0355) | (0.0688) | (0.0697) |
| treatment#1.Female |  |  | 1,079 |  |  |  |  |  | 1,04 |
|  |  |  | (0.0499) |  |  |  |  |  | (0.053) |
| post_earthquake#Female |  |  | 1,054 |  |  |  |  |  | 1,042 |
|  |  |  | (0.0516) |  |  |  |  |  | (0.0557) |
| treatment#post_earthquake#Female |  |  | 0,984 |  |  |  |  |  | 1,07 |
|  |  |  | (0.0479) |  |  |  |  |  | (0.0574) |
| treatment#Maori |  |  |  | 1.612*** |  |  |  |  | 1.585*** |
|  |  |  |  | (0.107) |  |  |  |  | (0.117) |
| post_earthquake#Maori |  |  |  | 0,992 |  |  |  |  | 0,962 |
|  |  |  |  | (0.0743) |  |  |  |  | (0.0841) |
| treatment#post_earthquake#Maori |  |  |  | 0.455*** |  |  |  |  | 0.487*** |
|  |  |  |  | (0.0345) |  |  |  |  | (0.0441) |
| treatment#Sole parents with Dependent Children |  |  |  |  | 1.468*** |  |  |  | 1.524*** |
|  |  |  |  |  | (0.121) |  |  |  | (0.135) |
| post_earthquake#Sole parents with Dependent Children |  |  |  |  | 1,158 |  |  |  | 1,164 |
|  |  |  |  |  | (0.117) |  |  |  | (0.133) |
| treatment#post_earthquake#Sole parents with Dependent Children |  |  |  |  | 0.477*** |  |  |  | 0.455*** |
|  |  |  |  |  | (0.0472) |  |  |  | (0.0516) |
| treatment#Age (25-35) |  |  |  |  |  | 0.811*** |  |  | 0.787*** |
|  |  |  |  |  |  | (0.0531) |  |  | (0.0504) |
| post_earthquake#Age (25-35) |  |  |  |  |  | 1,041 |  |  | 1,028 |
|  |  |  |  |  |  | (0.0891) |  |  | (0.0876) |
| treatment#post_earthquake#Age (25-35) |  |  |  |  |  | 1,142 |  |  | 1.209** |
|  |  |  |  |  |  | (0.0982) |  |  | (0.106) |
| treatment#Migrant |  |  |  |  |  |  | 0,93 |  | 0,983 |
|  |  |  |  |  |  |  | (0.0444) |  | (0.0501) |
| post_earthquake#Migrant |  |  |  |  |  |  | 0,963 |  | 0,947 |
|  |  |  |  |  |  |  | (0.0488) |  | (0.052) |
| treatment#post_earthquake#Migrant |  |  |  |  |  |  | 1.248*** |  | 1.176*** |
|  |  |  |  |  |  |  | (0.0669) |  | (0.0711) |
| treatment#Employed during EQ |  |  |  |  |  |  |  | 0,987 | 0,971 |
|  |  |  |  |  |  |  |  | (0.0596) | (0.0607) |
| post_earthquake#Employed during EQ |  |  |  |  |  |  |  | 0.843*** | 0.830*** |
|  |  |  |  |  |  |  |  | (0.0532) | (0.0536) |
| treatment#post_earthquake#Employed during EQ |  |  |  |  |  |  |  | 1.183*** | 1.228*** |
|  |  |  |  |  |  |  |  | (0.0732) | (0.0781) |
| Constant | 5.375*** | 3.744*** | 3.823*** | 3.734*** | 3.780*** | 3.761*** | 3.720*** | 3.409*** | 3.432*** |
|  | (0.271) | (0.376) | (0.399) | (0.372) | (0.385) | (0.365) | (0.366) | (0.379) | (0.373) |
| **Observations** | **45,345** | **45,312** | **45,312** | **45,312** | **45,312** | **45,312** | **45,312** | **45,312** | **45,312** |
| *Note: All models are with years fixed effect. Cluster standard errors (at the Territorial Authority level) are in parentheses. *p < 0.1, **p < 0.05, ***p < 0.01* | | | | | | | | | |

**Table 5: Full model - Impact of Earthquake to Life Satisfaction (Model 2)**

| **Variables** | **Life Satisfaction** | | | | | | | | | | | | | | | | |
| --- | --- | --- | --- | --- | --- | --- | --- | --- | --- | --- | --- | --- | --- | --- | --- | --- | --- |
|  | (1) | | (2) | | (3) | | (4) | | (5) | | (6) | | (7) | (8) | | (9) | |
| treatment | 1.008 | 0.95 | | 0.955 | | 0.93 | | 0.937 | | 0.997 | | 0.951 | | | 1.047 | | 1.089 |
|  | (0.0688) | (0.0506) | | (0.103) | | (0.0494) | | (0.0625) | | (0.0633) | | (0.0386) | | | (0.176) | | (0.233) |
| treatment * post EQ | 0.789*** | 0.797*** | | 0.824*** | | 0.836*** | | 0.831** | | 0.770*** | | 0.784*** | | | 0.672*** | | 0.687*** |
|  | (0.0467) | (0.0487) | | (0.0467) | | (0.0573) | | (0.0632) | | (0.052) | | (0.0341) | | | (0.0926) | | (0.0743) |
| Age during the EQ (Ref: <25 y.o) |  |  | |  | |  | |  | |  | |  | | |  | |  |
| 25-35 years old |  | 0.743*** | | 0.743*** | | 0.742*** | | 0.744*** | | 0.729*** | | 0.743*** | | | 0.747*** | | 0.740*** |
|  |  | (0.0375) | | (0.0375) | | (0.0377) | | (0.0373) | | (0.0444) | | (0.0485) | | | (0.0382) | | (0.0436) |
| 36-55 years old |  | 0.592*** | | 0.592*** | | 0.592*** | | 0.594*** | | 0.592*** | | 0.592*** | | | 0.596*** | | 0.598*** |
|  |  | (0.0241) | | (0.0241) | | (0.0242) | | (0.024) | | (0.0237) | | (0.0241) | | | (0.0246) | | (0.0241) |
| 56-65 years old |  | 0.726*** | | 0.726*** | | 0.726*** | | 0.728*** | | 0.725*** | | 0.726*** | | | 0.731*** | | 0.732*** |
|  |  | (0.0452) | | (0.0453) | | (0.0454) | | (0.045) | | (0.0448) | | (0.0452) | | | (0.0458) | | (0.0452) |
| >65 years old |  | 1.223*** | | 1.223*** | | 1.222*** | | 1.224*** | | 1.220*** | | 1.222*** | | | 1.230*** | | 1.230*** |
|  |  | (0.0778) | | (0.0779) | | (0.0779) | | (0.0781) | | (0.0769) | | (0.0778) | | | (0.0792) | | (0.0786) |
| Household size |  | 1.01 | | 1.01 | | 1.011 | | 1.01 | | 1.01 | | 1.01 | | | 1.01 | | 1.01 |
|  |  | (0.0161) | | (0.0162) | | (0.0162) | | (0.0161) | | (0.0161) | | (0.0162) | | | (0.0161) | | (0.0162) |
| Dependent children (1/0) |  | 1.255*** | | 1.255*** | | 1.253*** | | 1.257*** | | 1.254*** | | 1.254*** | | | 1.247*** | | 1.246*** |
|  |  | (0.0889) | | (0.0889) | | (0.089) | | (0.089) | | (0.0886) | | (0.0886) | | | (0.0883) | | (0.088) |
| Gender (1=Female) |  | 1.161*** | | 1.124** | | 1.161*** | | 1.160*** | | 1.161*** | | 1.161*** | | | 1.161*** | | 1.131** |
|  |  | (0.0319) | | (0.053) | | (0.0319) | | (0.0318) | | (0.0319) | | (0.0319) | | | (0.032) | | (0.0571) |
| European |  | 1.194*** | | 1.194*** | | 1.196*** | | 1.195*** | | 1.194*** | | 1.197*** | | | 1.194*** | | 1.199*** |
|  |  | (0.056) | | (0.056) | | (0.0553) | | (0.0557) | | (0.056) | | (0.0565) | | | (0.0562) | | (0.0555) |
| Māori |  | 0.860*** | | 0.860*** | | 0.869** | | 0.860*** | | 0.860*** | | 0.861*** | | | 0.861*** | | 0.889 |
|  |  | (0.0457) | | (0.0457) | | (0.0616) | | (0.0458) | | (0.0457) | | (0.0456) | | | (0.0457) | | (0.0681) |
| Pacific |  | 0.806*** | | 0.806*** | | 0.807*** | | 0.806*** | | 0.806*** | | 0.808*** | | | 0.807*** | | 0.810*** |
|  |  | (0.0447) | | (0.0449) | | (0.0448) | | (0.0447) | | (0.0447) | | (0.0441) | | | (0.0447) | | (0.0443) |
| Asian |  | 1.024 | | 1.024 | | 1.024 | | 1.025 | | 1.022 | | 1.029 | | | 1.02 | | 1.028 |
|  |  | (0.0686) | | (0.0685) | | (0.0695) | | (0.0688) | | (0.0685) | | (0.0666) | | | (0.0689) | | (0.0675) |
| MELAA |  | 0.876 | | 0.877 | | 0.877 | | 0.877 | | 0.874 | | 0.882 | | | 0.873 | | 0.879 |
|  |  | (0.153) | | (0.153) | | (0.154) | | (0.155) | | (0.152) | | (0.152) | | | (0.154) | | (0.153) |
| Other |  | 0.820* | | 0.820* | | 0.821* | | 0.819* | | 0.820* | | 0.821* | | | 0.819* | | 0.820* |
|  |  | (0.0965) | | (0.0965) | | (0.0964) | | (0.0964) | | (0.0964) | | (0.0966) | | | (0.0968) | | (0.0967) |
| Migrant to NZ |  | 1.091** | | 1.090** | | 1.091** | | 1.090** | | 1.091** | | 1.117** | | | 1.089** | | 1.127** |
|  |  | (0.0373) | | (0.0373) | | (0.0372) | | (0.0374) | | (0.0372) | | (0.055) | | | (0.0371) | | (0.0592) |
| Sole parents with dependent children |  | 0.613*** | | 0.613*** | | 0.613*** | | 0.561*** | | 0.613*** | | 0.614*** | | | 0.617*** | | 0.563*** |
|  |  | (0.0537) | | (0.0538) | | (0.0537) | | (0.0577) | | (0.0536) | | (0.0537) | | | (0.0538) | | (0.0584) |
| Couples with dependent children |  | 1.946*** | | 1.946*** | | 1.946*** | | 1.941*** | | 1.945*** | | 1.947*** | | | 1.965*** | | 1.962*** |
|  |  | (0.139) | | (0.139) | | (0.139) | | (0.138) | | (0.139) | | (0.139) | | | (0.139) | | (0.139) |
| Couples without dependent children |  | 2.273*** | | 2.273*** | | 2.271*** | | 2.271*** | | 2.273*** | | 2.273*** | | | 2.276*** | | 2.274*** |
|  |  | (0.0871) | | (0.0872) | | (0.087) | | (0.087) | | (0.0871) | | (0.087) | | | (0.0875) | | (0.0876) |
| Employed during the EQ (1/0) |  | 0.992 | | 0.991 | | 0.991 | | 0.992 | | 0.991 | | 0.991 | | | 1.111* | | 1.122* |
|  |  | (0.0351) | | (0.0352) | | (0.0351) | | (0.0351) | | (0.0351) | | (0.0352) | | | (0.0688) | | (0.0697) |
| treatment#1.Female |  |  | | 0.99 | |  | |  | |  | |  | | |  | | 0.964 |
|  |  |  | | (0.114) | |  | |  | |  | |  | | |  | | (0.104) |
| post_earthquake#Female |  |  | | 1.054 | |  | |  | |  | |  | | |  | | 1.043 |
|  |  |  | | (0.0516) | |  | |  | |  | |  | | |  | | (0.0558) |
| treatment#post_earthquake#Female |  |  | | 0.941 | |  | |  | |  | |  | | |  | | 0.997 |
|  |  |  | | (0.0673) | |  | |  | |  | |  | | |  | | (0.0912) |
| treatment#Maori |  |  | |  | | 1.519*** | |  | |  | |  | | |  | | 1.492*** |
|  |  |  | |  | | (0.147) | |  | |  | |  | | |  | | (0.138) |
| post_earthquake#Maori |  |  | |  | | 0.992 | |  | |  | |  | | |  | | 0.962 |
|  |  |  | |  | | (0.0742) | |  | |  | |  | | |  | | (0.084) |
| treatment#post_earthquake#Maori |  |  | |  | | 0.499*** | |  | |  | |  | | |  | | 0.525*** |
|  |  |  | |  | | (0.0758) | |  | |  | |  | | |  | | (0.0689) |
| treatment#Sole parents with Dependent Children |  |  | |  | |  | | 1.161 | |  | |  | | |  | | 1.24 |
|  |  |  | |  | |  | | (0.308) | |  | |  | | |  | | (0.298) |
| post_earthquake#Sole parents with Dependent Children |  |  | |  | | 1.158 | |  | |  | |  | | | 1.164 | | 1.164 |
|  |  |  | |  | |  | | (0.117) | |  | |  | | |  | | (0.133) |
| treatment#post_earthquake#Sole parents with Dependent Children |  |  | | 0.627 | |  | |  | |  | | 0.611 | | | 0.611 | | 0.455*** |
|  |  |  | |  | |  | | (0.184) | |  | |  | | |  | | (0.196) |
| treatment#Age (25-35) |  |  | |  | |  | |  | | 0.760*** | |  | | |  | | 0.748*** |
|  |  |  | |  | |  | |  | | (0.0777) | |  | | |  | | (0.069) |
| post_earthquake#Age (25-35) |  |  | |  | |  | |  | | 1.041 | |  | | |  | | 1.028 |
|  |  |  | |  | |  | |  | | (0.0891) | |  | | |  | | (0.0876) |
| treatment#post_earthquake#Age (25-35) |  |  | |  | |  | |  | | 1.190* | |  | | |  | | 1.235** |
|  |  |  | |  | |  | |  | | (0.116) | |  | | |  | | (0.116) |
| treatment#Migrant |  |  | |  | |  | |  | |  | | 0.992 | | |  | | 1.025 |
|  |  |  | |  | |  | |  | |  | | (0.11) | | |  | | (0.0932) |
| post_earthquake#Migrant |  |  | |  | |  | |  | |  | | 0.961 | | |  | | 0.946 |
|  |  |  | |  | |  | |  | |  | | (0.0485) | | |  | | (0.0517) |
| treatment#post_earthquake#Migrant |  |  | |  | |  | |  | |  | | 1.083 | | |  | | 1.031 |
|  |  |  | |  | |  | |  | |  | | (0.214) | | |  | | (0.189) |
| treatment#Employed during EQ |  |  | |  | |  | |  | |  | |  | | | 0.883 | | 0.875 |
|  |  |  | |  | |  | |  | |  | |  | | | (0.136) | | (0.124) |
| post_earthquake#Employed during EQ |  |  | |  | |  | |  | |  | |  | | | 0.844*** | | 0.831*** |
|  |  |  | |  | |  | |  | |  | |  | | | (0.0531) | | (0.0535) |
| treatment#post_earthquake#Employed during EQ |  |  | |  | |  | |  | |  | |  | | | 1.248** | | 1.275*** |
|  |  |  | |  | |  | |  | |  | |  | | | (0.133) | | (0.116) |
| Constant | 5.382*** | 3.774*** | | 3.844*** | | 3.764*** | | 3.811*** | | 3.789*** | | 3.746*** | | | 3.428*** | | 3.441*** |
|  | (0.271) | (0.377) | | (0.398) | | (0.373) | | (0.386) | | (0.365) | | (0.367) | | | (0.38) | | (0.373) |
| **Observations** | **45,600** | **45,564** | | **45,564** | | **45,564** | | **45,564** | | **45,564** | | **45,564** | | | **45,564** | | **45,564** |
| *Note: All models are with years fixed effect. Cluster standard errors (at the Territorial Authority level) are in parentheses. *p < 0.1, **p < 0.05, ***p < 0.01* | | | | | | | | | | | | | | | | | |

**Table 6: Full model - Impact of Earthquake to Life Satisfaction (Model 3)**

| **Variables** | **Life Satisfaction** | | | | | | | | |
| --- | --- | --- | --- | --- | --- | --- | --- | --- | --- |
|  | (1) | (2) | (3) | (4) | (5) | (6) | (7) | (8) | (9) |
| treatment | 0.925 | 0.886** | 0.860*** | 0.872** | 0.899* | 0.908 | 0.880** | 0.972 | 0.93 |
|  | (0.071) | (0.0493) | (0.0481) | (0.0498) | (0.0518) | (0.0594) | (0.0497) | (0.113) | (0.123) |
| treatment * post EQ | 0.908* | 0.897** | 0.922** | 0.910* | 0.895** | 0.886** | 0.867*** | 0.859 | 0.847* |
|  | (0.0481) | (0.0416) | (0.0355) | (0.0456) | (0.0489) | (0.0483) | (0.0395) | (0.0954) | (0.0736) |
| Age during the EQ (Ref: <25 y.o) |  |  |  |  |  |  |  |  |  |
| 25-35 years old |  | 0.712*** | 0.712*** | 0.712*** | 0.713*** | 0.703*** | 0.712*** | 0.716*** | 0.714*** |
|  |  | (0.0445) | (0.0444) | (0.0446) | (0.0444) | (0.0483) | (0.0503) | (0.0451) | (0.0484) |
| 36-55 years old |  | 0.568*** | 0.568*** | 0.568*** | 0.570*** | 0.568*** | 0.568*** | 0.572*** | 0.574*** |
|  |  | (0.0317) | (0.0317) | (0.0318) | (0.0318) | (0.0314) | (0.0317) | (0.0321) | (0.032) |
| 56-65 years old |  | 0.701*** | 0.701*** | 0.701*** | 0.703*** | 0.700*** | 0.701*** | 0.706*** | 0.707*** |
|  |  | (0.0429) | (0.0429) | (0.043) | (0.0427) | (0.0426) | (0.0428) | (0.0435) | (0.043) |
| >65 years old |  | 1.202*** | 1.202*** | 1.202*** | 1.202*** | 1.199*** | 1.201*** | 1.209*** | 1.209*** |
|  |  | (0.0706) | (0.0706) | (0.0705) | (0.0707) | (0.0698) | (0.0705) | (0.0719) | (0.0712) |
| Household size |  | 1.012 | 1.012 | 1.012 | 1.012 | 1.012 | 1.012 | 1.011 | 1.011 |
|  |  | (0.0156) | (0.0157) | (0.0157) | (0.0156) | (0.0156) | (0.0156) | (0.0156) | (0.0156) |
| Dependent children (1/0) |  | 1.227*** | 1.227*** | 1.227*** | 1.229*** | 1.226*** | 1.227*** | 1.220*** | 1.222*** |
|  |  | (0.0859) | (0.086) | (0.0862) | (0.0857) | (0.0855) | (0.0853) | (0.0854) | (0.0842) |
| Gender (1=Female) |  | 1.168*** | 1.125** | 1.168*** | 1.168*** | 1.168*** | 1.168*** | 1.167*** | 1.130** |
|  |  | (0.0315) | (0.0532) | (0.0317) | (0.0315) | (0.0315) | (0.0317) | (0.0315) | (0.0571) |
| European |  | 1.176*** | 1.176*** | 1.174*** | 1.177*** | 1.177*** | 1.179*** | 1.176*** | 1.176*** |
|  |  | (0.0624) | (0.0625) | (0.0633) | (0.0618) | (0.0622) | (0.0634) | (0.0626) | (0.0645) |
| Māori |  | 0.864*** | 0.864*** | 0.864** | 0.862*** | 0.864*** | 0.862*** | 0.864*** | 0.877* |
|  |  | (0.0416) | (0.0416) | (0.061) | (0.0419) | (0.0417) | (0.0418) | (0.0418) | (0.0691) |
| Pacific |  | 0.790*** | 0.790*** | 0.789*** | 0.789*** | 0.790*** | 0.794*** | 0.791*** | 0.791*** |
|  |  | (0.0477) | (0.048) | (0.0485) | (0.0478) | (0.0478) | (0.0466) | (0.0478) | (0.0482) |
| Asian |  | 1.006 | 1.006 | 1.004 | 1.007 | 1.005 | 1.009 | 1.003 | 1.005 |
|  |  | (0.0664) | (0.0664) | (0.0674) | (0.0662) | (0.0664) | (0.0641) | (0.0666) | (0.0652) |
| MELAA |  | 0.787* | 0.788* | 0.786* | 0.787* | 0.786* | 0.788* | 0.784* | 0.782* |
|  |  | (0.102) | (0.102) | (0.102) | (0.102) | (0.102) | (0.102) | (0.102) | (0.102) |
| Other |  | 0.780** | 0.781** | 0.779** | 0.779** | 0.781** | 0.781** | 0.779** | 0.777** |
|  |  | (0.0935) | (0.0936) | (0.094) | (0.0934) | (0.0935) | (0.0939) | (0.0941) | (0.0948) |
| Migrant to NZ |  | 1.111*** | 1.111*** | 1.110*** | 1.110*** | 1.111*** | 1.115** | 1.109*** | 1.123** |
|  |  | (0.0348) | (0.0349) | (0.0347) | (0.0346) | (0.0348) | (0.0542) | (0.0346) | (0.0589) |
| Sole parents with dependent children |  | 0.623*** | 0.623*** | 0.623*** | 0.576*** | 0.623*** | 0.624*** | 0.627*** | 0.577*** |
|  |  | (0.05) | (0.0501) | (0.05) | (0.0599) | (0.0499) | (0.05) | (0.05) | (0.0609) |
| Couples with dependent children |  | 2.023*** | 2.022*** | 2.023*** | 2.016*** | 2.022*** | 2.025*** | 2.042*** | 2.036*** |
|  |  | (0.161) | (0.161) | (0.161) | (0.16) | (0.16) | (0.161) | (0.162) | (0.159) |
| Couples without dependent children |  | 2.264*** | 2.264*** | 2.264*** | 2.262*** | 2.265*** | 2.265*** | 2.268*** | 2.267*** |
|  |  | (0.08) | (0.0801) | (0.0799) | (0.0801) | (0.0801) | (0.0801) | (0.0804) | (0.0804) |
| Employed during the EQ (1/0) |  | 0.984 | 0.984 | 0.984 | 0.985 | 0.984 | 0.985 | 1.111* | 1.120* |
|  |  | (0.0312) | (0.0313) | (0.0312) | (0.0312) | (0.0312) | (0.0313) | (0.069) | (0.0699) |
| treatment#1.Female |  |  | 1.056 |  |  |  |  |  | 1.085 |
|  |  |  | (0.05) |  |  |  |  |  | (0.057) |
| post_earthquake#Female |  |  | 1.054 |  |  |  |  |  | 1.042 |
|  |  |  | (0.0517) |  |  |  |  |  | (0.0558) |
| treatment#post_earthquake#Female |  |  | 0.95 |  |  |  |  |  | 0.94 |
|  |  |  | (0.0857) |  |  |  |  |  | (0.0971) |
| treatment#Maori |  |  |  | 1.274*** |  |  |  |  | 1.321*** |
|  |  |  |  | (0.0899) |  |  |  |  | (0.104) |
| post_earthquake#Maori |  |  |  | 0.989 |  |  |  |  | 0.96 |
|  |  |  |  | (0.0737) |  |  |  |  | (0.0836) |
| treatment#post_earthquake#Maori |  |  |  | 0.793** |  |  |  |  | 0.812** |
|  |  |  |  | (0.0741) |  |  |  |  | (0.0813) |
| treatment#Sole parents with Dependent Children |  |  |  |  | 0.815*** |  |  |  | 0.796*** |
|  |  |  |  |  | (0.0648) |  |  |  | (0.0701) |
| post_earthquake#Sole parents with Dependent Children |  |  |  |  | 1.157 |  |  |  | 1,164 |
|  |  |  |  |  | (0.117) |  |  |  | (0.133) |
| treatment#post_earthquake#Sole parents with Dependent Children |  |  |  |  | 1.071 |  |  |  | 1.117 |
|  |  |  |  |  | (0.169) |  |  |  | (0.2) |
| treatment#Age (25-35) |  |  |  |  |  | 0.848* |  |  | 0.850* |
|  |  |  |  |  |  | (0.0812) |  |  | (0.0817) |
| post_earthquake#Age (25-35) |  |  |  |  |  | 1.042 |  |  | 1.029 |
|  |  |  |  |  |  | (0.0894) |  |  | (0.0878) |
| treatment#post_earthquake#Age (25-35) |  |  |  |  |  | 1.085 |  |  | 1.077 |
|  |  |  |  |  |  | (0.116) |  |  | (0.109) |
| treatment#Migrant |  |  |  |  |  |  | 1.037 |  | 1.041 |
|  |  |  |  |  |  |  | (0.048) |  | (0.052) |
| post_earthquake#Migrant |  |  |  |  |  |  | 0.968 |  | 0.953 |
|  |  |  |  |  |  |  | (0.0503) |  | (0.0534) |
| treatment#post_earthquake#Migrant |  |  |  |  |  |  | 1.165* |  | 1.166* |
|  |  |  |  |  |  |  | (0.098) |  | (0.0985) |
| treatment#Employed during EQ |  |  |  |  |  |  |  | 0.89 | 0.908 |
|  |  |  |  |  |  |  |  | (0.0806) | (0.0817) |
| post_earthquake#Employed during EQ |  |  |  |  |  |  |  | 0.851** | 0.837*** |
|  |  |  |  |  |  |  |  | (0.0536) | (0.0542) |
| treatment#post_earthquake#Employed during EQ |  |  |  |  |  |  |  | 1.054 | 1.064 |
|  |  |  |  |  |  |  |  | (0.104) | (0.0945) |
| Constant | 5.341*** | 3.898*** | 3.979*** | 3.903*** | 3.929*** | 3.908*** | 3.887*** | 3.524*** | 3.581*** |
|  | (0.253) | (0.388) | (0.413) | (0.39) | (0.39) | (0.375) | (0.388) | (0.388) | (0.404) |
| **Observations** | **49,608** | **49,572** | **49,572** | **49,572** | **49,572** | **49,572** | **49,572** | **49,569** | **49,572** |
| *Note: All models are with years fixed effect. Cluster standard errors (at the Territorial Authority level) are in parentheses. *p < 0.1, **p < 0.05, ***p < 0.01* | | | | | | | | | |

**Table 7: Full model - Impact of Earthquake to General Health (Model 1)**

| **Variables** | **General Health** | | | | | | | | |
| --- | --- | --- | --- | --- | --- | --- | --- | --- | --- |
|  | (1) | (2) | (3) | (4) | (5) | (6) | (7) | (8) | (9) |
| treatment | 1.300*** | 1.249*** | 1.305*** | 1.195*** | 1.323*** | 1.270*** | 1.239*** | 1.529*** | 1.440*** |
|  | (0.0571) | (0.0475) | (0.0673) | (0.0464) | (0.0529) | (0.0489) | (0.0531) | (0.0995) | (0.11) |
| treatment * post EQ | 0.797*** | 0.813*** | 0.848*** | 0.894** | 0.790*** | 0.809*** | 0.798*** | 0.707*** | 0.872 |
|  | (0.0437) | (0.0347) | (0.0489) | (0.042) | (0.0344) | (0.0354) | (0.0395) | (0.056) | (0.0817) |
| Age during the EQ (Ref: <25 y.o) |  |  |  |  |  |  |  |  |  |
| 25-35 years old |  | 0.723*** | 0.723*** | 0.721*** | 0.723*** | 0.724*** | 0.723*** | 0.727*** | 0.722*** |
|  |  | (0.059) | (0.0589) | (0.0587) | (0.0589) | (0.0743) | (0.0684) | (0.0596) | (0.0717) |
| 36-55 years old |  | 0.513*** | 0.513*** | 0.512*** | 0.512*** | 0.513*** | 0.513*** | 0.516*** | 0.515*** |
|  |  | (0.0451) | (0.045) | (0.045) | (0.0448) | (0.045) | (0.0449) | (0.0459) | (0.0452) |
| 56-65 years old |  | 0.396*** | 0.396*** | 0.396*** | 0.396*** | 0.396*** | 0.396*** | 0.399*** | 0.398*** |
|  |  | (0.0462) | (0.0461) | (0.0461) | (0.046) | (0.0461) | (0.0461) | (0.0466) | (0.0463) |
| >65 years old |  | 0.297*** | 0.297*** | 0.297*** | 0.297*** | 0.297*** | 0.297*** | 0.299*** | 0.299*** |
|  |  | (0.0329) | (0.0329) | (0.0328) | (0.0328) | (0.0329) | (0.0328) | (0.0333) | (0.0331) |
| Household size |  | 0.970** | 0.970** | 0.970** | 0.970** | 0.970** | 0.970** | 0.970** | 0.970** |
|  |  | (0.0146) | (0.0146) | (0.0146) | (0.0146) | (0.0146) | (0.0146) | (0.0146) | (0.0146) |
| Dependent children (1/0) |  | 1.192** | 1.192** | 1.189** | 1.193** | 1.192** | 1.191** | 1.187** | 1.184** |
|  |  | (0.0953) | (0.0953) | (0.0948) | (0.0956) | (0.0952) | (0.0955) | (0.0952) | (0.0946) |
| Gender (1=Female) |  | 1.034 | 1.015 | 1.034 | 1.034 | 1.034 | 1.034 | 1.034 | 1.008 |
|  |  | (0.0281) | (0.0476) | (0.028) | (0.0281) | (0.028) | (0.0281) | (0.0281) | (0.0455) |
| European |  | 1.173*** | 1.173*** | 1.175*** | 1.173*** | 1.173*** | 1.179*** | 1.173*** | 1.181*** |
|  |  | (0.0565) | (0.0565) | (0.0569) | (0.0565) | (0.0565) | (0.0568) | (0.0566) | (0.0572) |
| Maori |  | 0.672*** | 0.672*** | 0.692*** | 0.671*** | 0.672*** | 0.673*** | 0.672*** | 0.700*** |
|  |  | (0.0392) | (0.0392) | (0.0452) | (0.0392) | (0.0392) | (0.0391) | (0.0392) | (0.0431) |
| Pacific |  | 0.821** | 0.822** | 0.823** | 0.822** | 0.821** | 0.826** | 0.822** | 0.828** |
|  |  | (0.0697) | (0.0699) | (0.0705) | (0.07) | (0.0698) | (0.0693) | (0.0699) | (0.0704) |
| Asian |  | 1.123** | 1.123** | 1.124** | 1.123** | 1.122** | 1.132** | 1.119** | 1.131** |
|  |  | (0.0616) | (0.0616) | (0.0616) | (0.0616) | (0.0614) | (0.0613) | (0.0609) | (0.0608) |
| MELAA |  | 0.989 | 0.99 | 0.989 | 0.99 | 0.988 | 0.998 | 0.986 | 0.999 |
|  |  | (0.109) | (0.109) | (0.11) | (0.11) | (0.11) | (0.109) | (0.11) | (0.111) |
| Other |  | 0.989 | 0.989 | 0.99 | 0.988 | 0.989 | 0.991 | 0.988 | 0.991 |
|  |  | (0.0954) | (0.0954) | (0.0956) | (0.0953) | (0.0954) | (0.0954) | (0.0957) | (0.0958) |
| Migrant to NZ |  | 1.090** | 1.089** | 1.090** | 1.089** | 1.090** | 1.134** | 1.088** | 1.148** |
|  |  | (0.0424) | (0.0423) | (0.0425) | (0.0423) | (0.0424) | (0.0663) | (0.0423) | (0.0672) |
| Sole parents with dependent children |  | 0.828* | 0.828* | 0.828* | 0.859 | 0.828* | 0.828* | 0.831* | 0.862 |
|  |  | (0.0851) | (0.0851) | (0.085) | (0.107) | (0.0851) | (0.0854) | (0.0855) | (0.103) |
| Couples with dependent children |  | 1.771*** | 1.771*** | 1.773*** | 1.770*** | 1.770*** | 1.772*** | 1.782*** | 1.786*** |
|  |  | (0.162) | (0.162) | (0.162) | (0.161) | (0.162) | (0.162) | (0.162) | (0.163) |
| Couples without dependent children |  | 1.588*** | 1.588*** | 1.587*** | 1.588*** | 1.588*** | 1.588*** | 1.589*** | 1.589*** |
|  |  | (0.0641) | (0.0642) | (0.0638) | (0.0643) | (0.0642) | (0.0641) | (0.0645) | (0.0645) |
| Employed during the EQ (1/0) |  | 0.835*** | 0.835*** | 0.835*** | 0.835*** | 0.835*** | 0.834*** | 0.931 | 0.932 |
|  |  | (0.0306) | (0.0307) | (0.0307) | (0.0306) | (0.0307) | (0.0307) | (0.0616) | (0.0615) |
| treatment#1.Female |  |  | 0.929 |  |  |  |  |  | 1.003 |
|  |  |  | (0.0429) |  |  |  |  |  | (0.0442) |
| post_earthquake#Female |  |  | 1.034 |  |  |  |  |  | 1.042 |
|  |  |  | (0.0537) |  |  |  |  |  | (0.0517) |
| treatment#post_earthquake#Female |  |  | 0.928 |  |  |  |  |  | 0.888** |
|  |  |  | (0.0484) |  |  |  |  |  | (0.0438) |
| treatment#Maori |  |  |  | 4.035*** |  |  |  |  | 4.281*** |
|  |  |  |  | (0.239) |  |  |  |  | (0.242) |
| post_earthquake#Maori |  |  |  | 0.972 |  |  |  |  | 0.957 |
|  |  |  |  | (0.0674) |  |  |  |  | (0.0667) |
| treatment#post_earthquake#Maori |  |  |  | 0.151*** |  |  |  |  | 0.146*** |
|  |  |  |  | (0.0103) |  |  |  |  | (0.00993) |
| treatment#Sole parents with Dependent Children |  |  |  |  | 0.334*** |  |  |  | 0.338*** |
|  |  |  |  |  | (0.032) |  |  |  | (0.0308) |
| post_earthquake#Sole parents with Dependent Children |  |  |  |  | 0.971 |  |  |  | 1,164 |
|  |  |  |  |  | (0.113) |  |  |  | (0.108) |
| treatment#post_earthquake#Sole parents with Dependent Children |  |  |  |  | 1.997*** |  |  |  | 2.171*** |
|  |  |  |  |  | (0.225) |  |  |  | (0.231) |
| treatment#Age (25-35) |  |  |  |  |  | 0.877* |  |  | 0.972 |
|  |  |  |  |  |  | (0.0686) |  |  | (0.0735) |
| post_earthquake#Age (25-35) |  |  |  |  |  | 1.004 |  |  | 1.012 |
|  |  |  |  |  |  | (0.0981) |  |  | (0.095) |
| treatment#post_earthquake#Age (25-35) |  |  |  |  |  | 1.027 |  |  | 0.936 |
|  |  |  |  |  |  | (0.0998) |  |  | (0.0872) |
| treatment#Migrant |  |  |  |  |  |  | 1.044 |  | 1.044 |
|  |  |  |  |  |  |  | (0.0612) |  | (0.0618) |
| post_earthquake#Migrant |  |  |  |  |  |  | 0.939 |  | 0.924 |
|  |  |  |  |  |  |  | (0.0684) |  | (0.0676) |
| treatment#post_earthquake#Migrant |  |  |  |  |  |  | 1.088 |  | 1.007 |
|  |  |  |  |  |  |  | (0.0793) |  | (0.0749) |
| treatment#Employed during EQ |  |  |  |  |  |  |  | 0.782*** | 0.848** |
|  |  |  |  |  |  |  |  | (0.0502) | (0.0547) |
| post_earthquake#Employed during EQ |  |  |  |  |  |  |  | 0.866* | 0.861** |
|  |  |  |  |  |  |  |  | (0.0647) | (0.0645) |
| treatment#post_earthquake#Employed during EQ |  |  |  |  |  |  |  | 1.183** | 1.074 |
|  |  |  |  |  |  |  |  | (0.0851) | (0.0783) |
| Constant | 5.437*** | 9.510*** | 9.617*** | 9.461*** | 9.492*** | 9.511*** | 9.392*** | 8.651*** | 8.555*** |
|  | (0.387) | (1.456) | (1.542) | (1.445) | (1.474) | (1.455) | (1.418) | (1.373) | (1.369) |
| **Observations** | **45,384** | **45,348** | **45,348** | **45,348** | **45,348** | **45,348** | **45,348** | **45,348** | **45,348** |
| *Note: All models are with years fixed effect. Cluster standard errors (at the Territorial Authority level) are in parentheses. *p < 0.1, **p < 0.05, ***p < 0.01* | | | | | | | | | |

**Table 8: Full model - Impact of Earthquake to General Health (Model 2)**

| **Variables** | **General Health** | | | | | | | | |
| --- | --- | --- | --- | --- | --- | --- | --- | --- | --- |
|  | (1) | (2) | (3) | (4) | (5) | (6) | (7) | (8) | (9) |
| treatment | 1.202* | 1.16 | 1.281*** | 1.144* | 1.219* | 1.180* | 1.109 | 1.418*** | 1.422*** |
|  | (0.13) | (0.115) | (0.0771) | (0.0801) | (0.133) | (0.117) | (0.156) | (0.171) | (0.131) |
| treatment * post EQ | 0.871 | 0.882 | 0.876 | 0.93 | 0.862 | 0.885 | 0.906 | 0.776* | 0.902 |
|  | (0.112) | (0.106) | (0.0753) | (0.0742) | (0.109) | (0.108) | (0.158) | (0.116) | (0.11) |
| Age during the EQ (Ref: <25 y.o) |  |  |  |  |  |  |  |  |  |
| 25-35 years old |  | 0.723*** | 0.723*** | 0.722*** | 0.723*** | 0.726*** | 0.723*** | 0.727*** | 0.724*** |
|  |  | (0.0587) | (0.0586) | (0.0585) | (0.0586) | (0.0745) | (0.068) | (0.0594) | (0.072) |
| 36-55 years old |  | 0.514*** | 0.514*** | 0.514*** | 0.514*** | 0.514*** | 0.514*** | 0.517*** | 0.517*** |
|  |  | (0.0451) | (0.0451) | (0.045) | (0.0448) | (0.045) | (0.0449) | (0.0459) | (0.0453) |
| 56-65 years old |  | 0.396*** | 0.396*** | 0.396*** | 0.396*** | 0.396*** | 0.396*** | 0.399*** | 0.398*** |
|  |  | (0.0459) | (0.0459) | (0.0459) | (0.0458) | (0.0459) | (0.0458) | (0.0464) | (0.0461) |
| >65 years old |  | 0.297*** | 0.297*** | 0.297*** | 0.297*** | 0.297*** | 0.297*** | 0.299*** | 0.299*** |
|  |  | (0.0326) | (0.0326) | (0.0326) | (0.0326) | (0.0326) | (0.0326) | (0.0331) | (0.0329) |
| Household size |  | 0.970** | 0.970** | 0.971** | 0.970** | 0.970** | 0.970** | 0.970** | 0.970** |
|  |  | (0.0146) | (0.0146) | (0.0146) | (0.0146) | (0.0146) | (0.0146) | (0.0146) | (0.0145) |
| Dependent children (1/0) |  | 1.185** | 1.185** | 1.182** | 1.186** | 1.185** | 1.184** | 1.180** | 1.177** |
|  |  | (0.0944) | (0.0943) | (0.0938) | (0.0946) | (0.0942) | (0.0945) | (0.0942) | (0.0936) |
| Gender (1=Female) |  | 1.032 | 1.015 | 1.032 | 1.032 | 1.032 | 1.032 | 1.032 | 1.008 |
|  |  | (0.0279) | (0.0476) | (0.0279) | (0.0279) | (0.0279) | (0.0279) | (0.0279) | (0.0455) |
| European |  | 1.177*** | 1.177*** | 1.180*** | 1.177*** | 1.177*** | 1.184*** | 1.177*** | 1.186*** |
|  |  | (0.0564) | (0.0564) | (0.0568) | (0.0565) | (0.0564) | (0.0568) | (0.0565) | (0.0573) |
| Maori |  | 0.674*** | 0.674*** | 0.694*** | 0.673*** | 0.673*** | 0.675*** | 0.674*** | 0.702*** |
|  |  | (0.0392) | (0.0391) | (0.0453) | (0.0392) | (0.0392) | (0.0391) | (0.0392) | (0.0432) |
| Pacific |  | 0.821** | 0.822** | 0.823** | 0.822** | 0.821** | 0.827** | 0.822** | 0.829** |
|  |  | (0.0691) | (0.0693) | (0.0698) | (0.0694) | (0.0692) | (0.0688) | (0.0693) | (0.0699) |
| Asian |  | 1.126** | 1.127** | 1.128** | 1.127** | 1.126** | 1.138** | 1.123** | 1.137** |
|  |  | (0.0621) | (0.062) | (0.0621) | (0.0621) | (0.0618) | (0.062) | (0.0613) | (0.0616) |
| MELAA |  | 0.992 | 0.993 | 0.992 | 0.993 | 0.991 | 1.002 | 0.989 | 1.003 |
|  |  | (0.11) | (0.11) | (0.11) | (0.11) | (0.111) | (0.11) | (0.11) | (0.112) |
| Other |  | 0.976 | 0.976 | 0.978 | 0.976 | 0.976 | 0.98 | 0.976 | 0.98 |
|  |  | (0.0949) | (0.095) | (0.0952) | (0.0949) | (0.095) | (0.095) | (0.0953) | (0.0954) |
| Migrant to NZ |  | 1.091** | 1.091** | 1.092** | 1.091** | 1.091** | 1.135** | 1.090** | 1.150** |
|  |  | (0.0425) | (0.0424) | (0.0426) | (0.0424) | (0.0425) | (0.0664) | (0.0424) | (0.0673) |
| Sole parents with dependent children |  | 0.830* | 0.830* | 0.831* | 0.863 | 0.830* | 0.831* | 0.833* | 0.865 |
|  |  | (0.0849) | (0.0849) | (0.0848) | (0.107) | (0.0849) | (0.0852) | (0.0853) | (0.103) |
| Couples with dependent children |  | 1.766*** | 1.766*** | 1.767*** | 1.765*** | 1.765*** | 1.767*** | 1.778*** | 1.781*** |
|  |  | (0.16) | (0.16) | (0.16) | (0.16) | (0.16) | (0.16) | (0.161) | (0.161) |
| Couples without dependent children |  | 1.586*** | 1.586*** | 1.585*** | 1.586*** | 1.586*** | 1.586*** | 1.587*** | 1.586*** |
|  |  | (0.0636) | (0.0638) | (0.0633) | (0.0638) | (0.0637) | (0.0636) | (0.064) | (0.064) |
| Employed during the EQ (1/0) |  | 0.834*** | 0.833*** | 0.833*** | 0.834*** | 0.833*** | 0.833*** | 0.93 | 0.932 |
|  |  | (0.0305) | (0.0305) | (0.0305) | (0.0305) | (0.0305) | (0.0305) | (0.0615) | (0.0615) |
| treatment#1.Female |  |  | 0.849 |  |  |  |  |  | 0.891 |
|  |  |  | (0.0937) |  |  |  |  |  | (0.116) |
| post_earthquake#Female |  |  | 1.035 |  |  |  |  |  | 1.042 |
|  |  |  | (0.0538) |  |  |  |  |  | (0.0517) |
| treatment#post_earthquake#Female |  |  | 1.004 |  |  |  |  |  | 0.979 |
|  |  |  | (0.0991) |  |  |  |  |  | (0.105) |
| treatment#Maori |  |  |  | 1.325 |  |  |  |  | 1.442 |
|  |  |  |  | (0.948) |  |  |  |  | (0.974) |
| post_earthquake#Maori |  |  |  | 0.973 |  |  |  |  | 0.957 |
|  |  |  |  | (0.0675) |  |  |  |  | (0.0667) |
| treatment#post_earthquake#Maori |  |  |  | 0.508 |  |  |  |  | 0.478 |
|  |  |  |  | (0.434) |  |  |  |  | (0.385) |
| treatment#Sole parents with Dependent Children |  |  |  |  | 0.411*** |  |  |  | 0.447*** |
|  |  |  |  |  | (0.107) |  |  |  | (0.138) |
| post_earthquake#Sole parents with Dependent Children |  |  |  |  | 0.972 |  |  |  | 0.972 |
|  |  |  |  |  | (0.113) |  |  |  | (0.108) |
| treatment#post_earthquake#Sole parents with Dependent Children |  |  |  |  | 1.629* |  |  |  | 1.671* |
|  |  |  |  |  | (0.415) |  |  |  | (0.506) |
| treatment#Age (25-35) |  |  |  |  |  | 0.869* |  |  | 0.918 |
|  |  |  |  |  |  | (0.0695) |  |  | (0.0963) |
| post_earthquake#Age (25-35) |  |  |  |  |  | 1.005 |  |  | 1.013 |
|  |  |  |  |  |  | (0.0983) |  |  | (0.0952) |
| treatment#post_earthquake#Age (25-35) |  |  |  |  |  | 0.963 |  |  | 0.93 |
|  |  |  |  |  |  | (0.134) |  |  | (0.137) |
| treatment#Migrant |  |  |  |  |  |  | 1.294 |  | 1.272 |
|  |  |  |  |  |  |  | (0.365) |  | (0.335) |
| post_earthquake#Migrant |  |  |  |  |  |  | 0.937 |  | 0.922 |
|  |  |  |  |  |  |  | (0.068) |  | (0.0672) |
| treatment#post_earthquake#Migrant |  |  |  |  |  |  | 0.845 |  | 0.813 |
|  |  |  |  |  |  |  | (0.291) |  | (0.25) |
| treatment#Employed during EQ |  |  |  |  |  |  |  | 0.784*** | 0.845** |
|  |  |  |  |  |  |  |  | (0.0513) | (0.0563) |
| post_earthquake#Employed during EQ |  |  |  |  |  |  |  | 0.865* | 0.860** |
|  |  |  |  |  |  |  |  | (0.0645) | (0.0643) |
| treatment#post_earthquake#Employed during EQ |  |  |  |  |  |  |  | 1.166** | 1.071 |
|  |  |  |  |  |  |  |  | (0.0862) | (0.0794) |
| Constant | 5.420*** | 9.477*** | 9.579*** | 9.426*** | 9.450*** | 9.476*** | 9.350*** | 8.609*** | 8.489*** |
|  | (0.384) | (1.444) | (1.529) | (1.433) | (1.461) | (1.443) | (1.405) | (1.363) | (1.356) |
| **Observations** | **45,636** | **45,600** | **45,600** | **45,600** | **45,600** | **45,600** | **45,600** | **45,600** | **45,600** |
| *Note: All models are with years fixed effect. Cluster standard errors (at the Territorial Authority level) are in parentheses. *p < 0.1, **p < 0.05, ***p < 0.01* | | | | | | | | | |

**Table 9: Full model - Impact of Earthquake to General Health (Model 3)**

| **Variables** | **General Health** | | | | | | | | |
| --- | --- | --- | --- | --- | --- | --- | --- | --- | --- |
|  | (1) | (2) | (3) | (4) | (5) | (6) | (7) | (8) | (9) |
| treatment | 0.995 | 0.957 | 0.951 | 0.971 | 0.96 | 0.96 | 0.954 | 1.063 | 1.075 |
|  | (0.0531) | (0.0428) | (0.0602) | (0.0433) | (0.0443) | (0.0436) | (0.0504) | (0.0705) | (0.112) |
| treatment * post EQ | 1.01 | 0.988 | 1.03 | 0.978 | 0.995 | 0.989 | 0.955 | 0.985 | 0.962 |
|  | (0.061) | (0.0506) | (0.0793) | (0.0504) | (0.0547) | (0.0476) | (0.0564) | (0.0803) | (0.0981) |
| Age during the EQ (Ref: <25 y.o) |  |  |  |  |  |  |  |  |  |
| 25-35 years old |  | 0.729*** | 0.729*** | 0.729*** | 0.729*** | 0.733*** | 0.729*** | 0.733*** | 0.732*** |
|  |  | (0.0559) | (0.0558) | (0.0559) | (0.0557) | (0.074) | (0.0683) | (0.0565) | (0.0718) |
| 36-55 years old |  | 0.519*** | 0.519*** | 0.518*** | 0.518*** | 0.519*** | 0.519*** | 0.522*** | 0.521*** |
|  |  | (0.0427) | (0.0427) | (0.0427) | (0.0425) | (0.0427) | (0.0425) | (0.0435) | (0.0429) |
| 56-65 years old |  | 0.406*** | 0.406*** | 0.405*** | 0.405*** | 0.406*** | 0.406*** | 0.408*** | 0.408*** |
|  |  | (0.0469) | (0.0469) | (0.0469) | (0.0468) | (0.0469) | (0.0468) | (0.0473) | (0.0471) |
| >65 years old |  | 0.304*** | 0.304*** | 0.304*** | 0.304*** | 0.304*** | 0.304*** | 0.306*** | 0.305*** |
|  |  | (0.0331) | (0.033) | (0.033) | (0.033) | (0.0331) | (0.0329) | (0.0334) | (0.0333) |
| Household size |  | 0.968** | 0.968** | 0.968** | 0.968** | 0.968** | 0.968** | 0.968** | 0.967** |
|  |  | (0.0136) | (0.0136) | (0.0136) | (0.0136) | (0.0136) | (0.0136) | (0.0136) | (0.0135) |
| Dependent children (1/0) |  | 1.232** | 1.232** | 1.231** | 1.232** | 1.232** | 1.231** | 1.226** | 1.225** |
|  |  | (0.106) | (0.106) | (0.105) | (0.106) | (0.106) | (0.106) | (0.106) | (0.106) |
| Gender (1=Female) |  | 1.032 | 1.011 | 1.032 | 1.032 | 1.032 | 1.032 | 1.032 | 1.005 |
|  |  | (0.026) | (0.0475) | (0.026) | (0.026) | (0.0259) | (0.026) | (0.026) | (0.0455) |
| European |  | 1.182*** | 1.182*** | 1.184*** | 1.183*** | 1.182*** | 1.188*** | 1.182*** | 1.189*** |
|  |  | (0.0533) | (0.0533) | (0.0537) | (0.0534) | (0.0533) | (0.0537) | (0.0535) | (0.0541) |
| Maori |  | 0.678*** | 0.678*** | 0.694*** | 0.678*** | 0.678*** | 0.678*** | 0.678*** | 0.701*** |
|  |  | (0.0372) | (0.0371) | (0.045) | (0.0372) | (0.0372) | (0.037) | (0.0372) | (0.0426) |
| Pacific |  | 0.830** | 0.830** | 0.831** | 0.830** | 0.830** | 0.836** | 0.831** | 0.838** |
|  |  | (0.0683) | (0.0685) | (0.069) | (0.0685) | (0.0683) | (0.0678) | (0.0685) | (0.0687) |
| Asian |  | 1.157** | 1.157** | 1.158** | 1.157** | 1.157** | 1.166*** | 1.153** | 1.165*** |
|  |  | (0.0685) | (0.0686) | (0.0693) | (0.0687) | (0.0683) | (0.0672) | (0.0675) | (0.0673) |
| MELAA |  | 1.02 | 1.021 | 1.021 | 1.021 | 1.02 | 1.027 | 1.016 | 1.026 |
|  |  | (0.112) | (0.112) | (0.113) | (0.112) | (0.113) | (0.109) | (0.111) | (0.111) |
| Other |  | 0.93 | 0.93 | 0.931 | 0.93 | 0.93 | 0.932 | 0.928 | 0.931 |
|  |  | (0.0907) | (0.0908) | (0.0904) | (0.0907) | (0.0907) | (0.0907) | (0.0912) | (0.0911) |
| Migrant to NZ |  | 1.094** | 1.094** | 1.095** | 1.094** | 1.094** | 1.129** | 1.093** | 1.142** |
|  |  | (0.0407) | (0.0406) | (0.041) | (0.0406) | (0.0408) | (0.0666) | (0.0405) | (0.0675) |
| Sole parents with dependent children |  | 0.818** | 0.817** | 0.818** | 0.844 | 0.817** | 0.819** | 0.821** | 0.846 |
|  |  | (0.0812) | (0.0812) | (0.081) | (0.104) | (0.0812) | (0.0814) | (0.0816) | (0.101) |
| Couples with dependent children |  | 1.751*** | 1.751*** | 1.752*** | 1.751*** | 1.751*** | 1.753*** | 1.764*** | 1.768*** |
|  |  | (0.152) | (0.152) | (0.152) | (0.152) | (0.152) | (0.153) | (0.153) | (0.153) |
| Couples without dependent children |  | 1.599*** | 1.600*** | 1.600*** | 1.600*** | 1.599*** | 1.600*** | 1.601*** | 1.602*** |
|  |  | (0.0625) | (0.0626) | (0.0624) | (0.0627) | (0.0625) | (0.0627) | (0.063) | (0.0631) |
| Employed during the EQ (1/0) |  | 0.829*** | 0.828*** | 0.829*** | 0.829*** | 0.829*** | 0.829*** | 0.932 | 0.934 |
|  |  | (0.0289) | (0.029) | (0.0289) | (0.0289) | (0.029) | (0.0289) | (0.0617) | (0.0617) |
| treatment#1.Female |  |  | 1.011 |  |  |  |  |  | 1.015 |
|  |  |  | (0.0703) |  |  |  |  |  | (0.0794) |
| post_earthquake#Female |  |  | 1.037 |  |  |  |  |  | 1.044 |
|  |  |  | (0.054) |  |  |  |  |  | (0.0519) |
| treatment#post_earthquake#Female |  |  | 0.928 |  |  |  |  |  | 0.937 |
|  |  |  | (0.0745) |  |  |  |  |  | (0.0817) |
| treatment#Maori |  |  |  | 0.845* |  |  |  |  | 0.844 |
|  |  |  |  | (0.0803) |  |  |  |  | (0.099) |
| post_earthquake#Maori |  |  |  | 0.977 |  |  |  |  | 0.962 |
|  |  |  |  | (0.0677) |  |  |  |  | (0.0669) |
| treatment#post_earthquake#Maori |  |  |  | 1.134 |  |  |  |  | 1.191 |
|  |  |  |  | (0.195) |  |  |  |  | (0.225) |
| treatment#Sole parents with Dependent Children |  |  |  |  | 0.96 |  |  |  | 0.992 |
|  |  |  |  |  | (0.153) |  |  |  | (0.18) |
| post_earthquake#Sole parents with Dependent Children |  |  |  |  | 0.973 |  |  |  | 0.973 |
|  |  |  |  |  | (0.113) |  |  |  | (0.108) |
| treatment#post_earthquake#Sole parents with Dependent Children |  |  |  |  | 0.891 |  |  |  | 0.897 |
|  |  |  |  |  | (0.161) |  |  |  | (0.198) |
| treatment#Age (25-35) |  |  |  |  |  | 0.972 |  |  | 0.981 |
|  |  |  |  |  |  | (0.105) |  |  | (0.11) |
| post_earthquake#Age (25-35) |  |  |  |  |  | 0.998 |  |  | 1.006 |
|  |  |  |  |  |  | (0.0978) |  |  | (0.0948) |
| treatment#post_earthquake#Age (25-35) |  |  |  |  |  | 0.992 |  |  | 0.98 |
|  |  |  |  |  |  | (0.148) |  |  | (0.156) |
| treatment#Migrant |  |  |  |  |  |  | 1.017 |  | 0.994 |
|  |  |  |  |  |  |  | (0.0969) |  | (0.0995) |
| post_earthquake#Migrant |  |  |  |  |  |  | 0.936 |  | 0.922 |
|  |  |  |  |  |  |  | (0.0683) |  | (0.0677) |
| treatment#post_earthquake#Migrant |  |  |  |  |  |  | 1.173** |  | 1.180* |
|  |  |  |  |  |  |  | (0.0946) |  | (0.1) |
| treatment#Employed during EQ |  |  |  |  |  |  |  | 0.880* | 0.88 |
|  |  |  |  |  |  |  |  | (0.0684) | (0.0687) |
| post_earthquake#Employed during EQ |  |  |  |  |  |  |  | 0.867* | 0.861** |
|  |  |  |  |  |  |  |  | (0.0647) | (0.0644) |
| treatment#post_earthquake#Employed during EQ |  |  |  |  |  |  |  | 1.002 | 1.024 |
|  |  |  |  |  |  |  |  | (0.0932) | (0.094) |
| Constant | 5.299*** | 9.102*** | 9.212*** | 9.066*** | 9.078*** | 9.096*** | 9.003*** | 8.223*** | 8.159*** |
|  | (0.363) | (1.36) | (1.448) | (1.359) | (1.378) | (1.361) | (1.318) | (1.311) | (1.312) |
| **Observations** | **49,647** | **49,611** | **49,611** | **49,611** | **49,611** | **49,611** | **49,611** | **49,611** | **49,611** |
| *Note: All models are with years fixed effect. Cluster standard errors (at the Territorial Authority level) are in parentheses. *p < 0.1, **p < 0.05, ***p < 0.01* | | | | | | | | | |

**Table 10: Full model - Impact of Earthquake to Income Adequacy (Model 1)**

| **Variables** | **Income Adequacy** | | | | | | | | |
| --- | --- | --- | --- | --- | --- | --- | --- | --- | --- |
|  | (1) | (2) | (3) | (4) | (5) | (6) | (7) | (8) | (9) |
| treatment | 1.403*** | 1.236*** | 1.117** | 1.267*** | 1.250*** | 1.208*** | 1.129*** | 1.878*** | 1.468*** |
|  | (0.0753) | (0.0477) | (0.0542) | (0.0489) | (0.055) | (0.0489) | (0.045) | (0.0915) | (0.0908) |
| treatment * post EQ | 0.829** | 0.832*** | 0.998 | 0.808*** | 0.864*** | 0.904** | 0.942 | 0.620*** | 0.985 |
|  | (0.0651) | (0.0401) | (0.0719) | (0.0389) | (0.047) | (0.0455) | (0.0408) | (0.0502) | (0.0841) |
| Age during the EQ (Ref: <25 y.o) |  |  |  |  |  |  |  |  |  |
| 25-35 years old |  | 0.927 | 0.925 | 0.927 | 0.928 | 0.807*** | 0.928 | 0.926 | 0.801*** |
|  |  | (0.0537) | (0.054) | (0.0537) | (0.0539) | (0.0475) | (0.0674) | (0.0531) | (0.0462) |
| 36-55 years old |  | 0.767*** | 0.766*** | 0.767*** | 0.768*** | 0.763*** | 0.767*** | 0.766*** | 0.762*** |
|  |  | (0.0345) | (0.0347) | (0.0344) | (0.0348) | (0.0338) | (0.035) | (0.0338) | (0.0336) |
| 56-65 years old |  | 0.748*** | 0.746*** | 0.748*** | 0.749*** | 0.744*** | 0.748*** | 0.746*** | 0.741*** |
|  |  | (0.0341) | (0.0341) | (0.0339) | (0.0341) | (0.0334) | (0.0339) | (0.0339) | (0.0331) |
| >65 years old |  | 1.543*** | 1.540*** | 1.543*** | 1.544*** | 1.529*** | 1.542*** | 1.541*** | 1.522*** |
|  |  | (0.0869) | (0.0867) | (0.087) | (0.087) | (0.0868) | (0.0866) | (0.0873) | (0.0871) |
| Household size |  | 0.925*** | 0.925*** | 0.925*** | 0.925*** | 0.925*** | 0.925*** | 0.925*** | 0.925*** |
|  |  | (0.0113) | (0.0113) | (0.0113) | (0.0113) | (0.0113) | (0.0114) | (0.0113) | (0.0113) |
| Dependent children (1/0) |  | 0.817*** | 0.814*** | 0.817*** | 0.819*** | 0.818*** | 0.814*** | 0.820*** | 0.817*** |
|  |  | (0.0521) | (0.0521) | (0.0521) | (0.0526) | (0.0522) | (0.0516) | (0.0525) | (0.0526) |
| Gender (1=Female) |  | 0.938** | 1.038 | 0.938** | 0.937** | 0.938** | 0.937** | 0.937** | 1.048 |
|  |  | (0.0263) | (0.039) | (0.0262) | (0.0262) | (0.0264) | (0.0263) | (0.0262) | (0.0384) |
| European |  | 1.426*** | 1.428*** | 1.426*** | 1.427*** | 1.426*** | 1.439*** | 1.426*** | 1.440*** |
|  |  | (0.0812) | (0.081) | (0.0812) | (0.0811) | (0.0814) | (0.0859) | (0.0812) | (0.0857) |
| Maori |  | 0.663*** | 0.663*** | 0.678*** | 0.662*** | 0.663*** | 0.664*** | 0.662*** | 0.707*** |
|  |  | (0.0333) | (0.0333) | (0.0435) | (0.0334) | (0.0334) | (0.0335) | (0.0333) | (0.0494) |
| Pacific |  | 0.428*** | 0.429*** | 0.428*** | 0.429*** | 0.429*** | 0.432*** | 0.428*** | 0.432*** |
|  |  | (0.038) | (0.0379) | (0.038) | (0.0382) | (0.0383) | (0.037) | (0.038) | (0.0375) |
| Asian |  | 1.078 | 1.076 | 1.077 | 1.079 | 1.074 | 1.096** | 1.078 | 1.091* |
|  |  | (0.0515) | (0.0512) | (0.0516) | (0.0516) | (0.0514) | (0.0501) | (0.0511) | (0.0496) |
| MELAA |  | 0.742** | 0.741** | 0.741** | 0.743** | 0.739** | 0.758** | 0.742** | 0.752** |
|  |  | (0.103) | (0.102) | (0.102) | (0.103) | (0.102) | (0.106) | (0.103) | (0.105) |
| Other |  | 0.991 | 0.989 | 0.99 | 0.99 | 0.993 | 0.996 | 0.989 | 0.989 |
|  |  | (0.123) | (0.123) | (0.123) | (0.122) | (0.123) | (0.124) | (0.123) | (0.123) |
| Migrant to NZ |  | 0.937 | 0.937 | 0.937 | 0.936 | 0.937 | 1.02 | 0.937 | 1.038 |
|  |  | (0.0416) | (0.0416) | (0.0416) | (0.0416) | (0.0416) | (0.0596) | (0.0414) | (0.0633) |
| Sole parents with dependent children |  | 0.669*** | 0.672*** | 0.669*** | 0.649*** | 0.667*** | 0.672*** | 0.667*** | 0.638*** |
|  |  | (0.0512) | (0.0514) | (0.0512) | (0.0769) | (0.0516) | (0.0512) | (0.0515) | (0.0733) |
| Couples with dependent children |  | 2.527*** | 2.538*** | 2.527*** | 2.521*** | 2.517*** | 2.531*** | 2.516*** | 2.514*** |
|  |  | (0.221) | (0.224) | (0.221) | (0.223) | (0.222) | (0.219) | (0.223) | (0.225) |
| Couples without dependent children |  | 2.698*** | 2.700*** | 2.699*** | 2.698*** | 2.701*** | 2.697*** | 2.697*** | 2.701*** |
|  |  | (0.111) | (0.111) | (0.111) | (0.111) | (0.111) | (0.111) | (0.112) | (0.112) |
| Employed during the EQ (1/0) |  | 0.883*** | 0.883*** | 0.883*** | 0.883*** | 0.882*** | 0.881*** | 0.875** | 0.877** |
|  |  | (0.0308) | (0.0308) | (0.0309) | (0.0308) | (0.0309) | (0.0307) | (0.0547) | (0.054) |
| treatment#1.Female |  |  | 1.207*** |  |  |  |  |  | 1.240*** |
|  |  |  | (0.0455) |  |  |  |  |  | (0.0466) |
| post_earthquake#Female |  |  | 0.858*** |  |  |  |  |  | 0.844*** |
|  |  |  | (0.05) |  |  |  |  |  | (0.0455) |
| treatment#post_earthquake#Female |  |  | 0.726*** |  |  |  |  |  | 0.728*** |
|  |  |  | (0.0414) |  |  |  |  |  | (0.039) |
| treatment#Maori |  |  |  | 0.711*** |  |  |  |  | 0.740*** |
|  |  |  |  | (0.0415) |  |  |  |  | (0.0469) |
| post_earthquake#Maori |  |  |  | 0.968 |  |  |  |  | 0.913 |
|  |  |  |  | (0.067) |  |  |  |  | (0.0724) |
| treatment#post_earthquake#Maori |  |  |  | 1.451*** |  |  |  |  | 1.353*** |
|  |  |  |  | (0.1) |  |  |  |  | (0.112) |
| treatment#Sole parents with Dependent Children |  |  |  |  | 0.836** |  |  |  | 0.845** |
|  |  |  |  |  | (0.0726) |  |  |  | (0.0721) |
| post_earthquake#Sole parents with Dependent Children |  |  |  |  | 1.063 |  |  |  | 1.084 |
|  |  |  |  |  | (0.113) |  |  |  | (0.11) |
| treatment#post_earthquake#Sole parents with Dependent Children |  |  |  |  | 0.804** |  |  |  | 0.851 |
|  |  |  |  |  | (0.0889) |  |  |  | (0.0927) |
| treatment#Age (25-35) |  |  |  |  |  | 1.143** |  |  | 1.172*** |
|  |  |  |  |  |  | (0.0608) |  |  | (0.0612) |
| post_earthquake#Age (25-35) |  |  |  |  |  | 1.248*** |  |  | 1.254*** |
|  |  |  |  |  |  | (0.0969) |  |  | (0.0951) |
| treatment#post_earthquake#Age (25-35) |  |  |  |  |  | 0.615*** |  |  | 0.633*** |
|  |  |  |  |  |  | (0.0477) |  |  | (0.0479) |
| treatment#Migrant |  |  |  |  |  |  | 1.577*** |  | 1.464*** |
|  |  |  |  |  |  |  | (0.0836) |  | (0.0808) |
| post_earthquake#Migrant |  |  |  |  |  |  | 0.878* |  | 0.856* |
|  |  |  |  |  |  |  | (0.0674) |  | (0.0696) |
| treatment#post_earthquake#Migrant |  |  |  |  |  |  | 0.549*** |  | 0.574*** |
|  |  |  |  |  |  |  | (0.0431) |  | (0.0496) |
| treatment#Employed during EQ |  |  |  |  |  |  |  | 0.605*** | 0.654*** |
|  |  |  |  |  |  |  |  | (0.0354) | (0.0387) |
| post_earthquake#Employed during EQ |  |  |  |  |  |  |  | 1.026 | 1.02 |
|  |  |  |  |  |  |  |  | (0.0823) | (0.0793) |
| treatment#post_earthquake#Employed during EQ |  |  |  |  |  |  |  | 1.402*** | 1.254*** |
|  |  |  |  |  |  |  |  | (0.111) | (0.0945) |
| Constant | 4.982*** | 4.644*** | 4.370*** | 4.629*** | 4.663*** | 4.787*** | 4.521*** | 4.687*** | 4.357*** |
|  | (0.35) | (0.443) | (0.401) | (0.444) | (0.437) | (0.448) | (0.481) | (0.431) | (0.445) |
| **Observations** | **44,475** | **44,451** | **44,451** | **44,451** | **44,451** | **44,451** | **44,451** | **44,451** | **44,451** |
| *Note: All models are with years fixed effect. Cluster standard errors (at the Territorial Authority level) are in parentheses. *p < 0.1, **p < 0.05, ***p < 0.01* | | | | | | | | | |

**Table 11: Full model - Impact of Earthquake to Income Adequacy (Model 2)**

| **Variables** | **Income Adequacy** | | | | | | | | |
| --- | --- | --- | --- | --- | --- | --- | --- | --- | --- |
|  | (1) | (2) | (3) | (4) | (5) | (6) | (7) | (8) | (9) |
| treatment | 1.294** | 1.123 | 1.144** | 1.169 | 1.131 | 1.088 | 1.018 | 1.824*** | 1.632*** |
|  | (0.138) | (0.132) | (0.0624) | (0.118) | (0.141) | (0.135) | (0.125) | (0.132) | (0.198) |
| treatment * post EQ | 0.938 | 0.931 | 1.003 | 0.878 | 0.964 | 1.004 | 1.062 | 0.643*** | 0.863 |
|  | (0.145) | (0.122) | (0.0751) | (0.0892) | (0.127) | (0.121) | (0.145) | (0.0598) | (0.143) |
| Age during the EQ (Ref: <25 y.o) |  |  |  |  |  |  |  |  |  |
| 25-35 years old |  | 0.93 | 0.928 | 0.929 | 0.93 | 0.805*** | 0.931 | 0.928 | 0.800*** |
|  |  | (0.0536) | (0.0538) | (0.0535) | (0.0537) | (0.0476) | (0.0677) | (0.0529) | (0.0461) |
| 36-55 years old |  | 0.768*** | 0.767*** | 0.768*** | 0.769*** | 0.764*** | 0.768*** | 0.767*** | 0.763*** |
|  |  | (0.0343) | (0.0345) | (0.0342) | (0.0346) | (0.0336) | (0.0348) | (0.0336) | (0.0334) |
| 56-65 years old |  | 0.747*** | 0.745*** | 0.746*** | 0.747*** | 0.742*** | 0.747*** | 0.745*** | 0.740*** |
|  |  | (0.0339) | (0.0339) | (0.0337) | (0.0339) | (0.0332) | (0.0337) | (0.0337) | (0.0329) |
| >65 years old |  | 1.541*** | 1.538*** | 1.540*** | 1.541*** | 1.527*** | 1.540*** | 1.539*** | 1.520*** |
|  |  | (0.0862) | (0.086) | (0.0862) | (0.0862) | (0.086) | (0.0859) | (0.0866) | (0.0863) |
| Household size |  | 0.926*** | 0.926*** | 0.925*** | 0.926*** | 0.925*** | 0.926*** | 0.926*** | 0.926*** |
|  |  | (0.0113) | (0.0112) | (0.0112) | (0.0113) | (0.0113) | (0.0113) | (0.0112) | (0.0112) |
| Dependent children (1/0) |  | 0.808*** | 0.805*** | 0.808*** | 0.810*** | 0.809*** | 0.805*** | 0.811*** | 0.808*** |
|  |  | (0.0523) | (0.0524) | (0.0524) | (0.0528) | (0.0524) | (0.0518) | (0.0528) | (0.0529) |
| Gender (1=Female) |  | 0.934** | 1.037 | 0.934** | 0.934** | 0.934** | 0.934** | 0.933** | 1.047 |
|  |  | (0.0265) | (0.039) | (0.0264) | (0.0264) | (0.0266) | (0.0265) | (0.0264) | (0.0383) |
| European |  | 1.423*** | 1.424*** | 1.422*** | 1.423*** | 1.422*** | 1.436*** | 1.423*** | 1.436*** |
|  |  | (0.0811) | (0.0809) | (0.0811) | (0.081) | (0.0812) | (0.0858) | (0.0811) | (0.0856) |
| Maori |  | 0.662*** | 0.662*** | 0.678*** | 0.662*** | 0.663*** | 0.664*** | 0.662*** | 0.706*** |
|  |  | (0.0331) | (0.0331) | (0.0435) | (0.0332) | (0.0332) | (0.0333) | (0.0332) | (0.0493) |
| Pacific |  | 0.428*** | 0.429*** | 0.428*** | 0.429*** | 0.429*** | 0.432*** | 0.428*** | 0.433*** |
|  |  | (0.0381) | (0.038) | (0.0381) | (0.0383) | (0.0384) | (0.0372) | (0.0381) | (0.0376) |
| Asian |  | 1.074 | 1.072 | 1.073 | 1.075 | 1.07 | 1.092* | 1.074 | 1.087* |
|  |  | (0.0512) | (0.0508) | (0.0514) | (0.0513) | (0.0511) | (0.0499) | (0.0508) | (0.0496) |
| MELAA |  | 0.741** | 0.739** | 0.739** | 0.741** | 0.737** | 0.756** | 0.741** | 0.750** |
|  |  | (0.102) | (0.102) | (0.102) | (0.102) | (0.102) | (0.106) | (0.103) | (0.105) |
| Other |  | 0.978 | 0.976 | 0.977 | 0.977 | 0.98 | 0.982 | 0.976 | 0.976 |
|  |  | (0.121) | (0.121) | (0.121) | (0.121) | (0.122) | (0.123) | (0.121) | (0.122) |
| Migrant to NZ |  | 0.937 | 0.937 | 0.937 | 0.937 | 0.937 | 1.017 | 0.937 | 1.035 |
|  |  | (0.0414) | (0.0414) | (0.0414) | (0.0415) | (0.0414) | (0.0588) | (0.0413) | (0.0626) |
| Sole parents with dependent children |  | 0.675*** | 0.677*** | 0.674*** | 0.654*** | 0.672*** | 0.677*** | 0.672*** | 0.643*** |
|  |  | (0.052) | (0.0522) | (0.0521) | (0.0778) | (0.0524) | (0.0519) | (0.0523) | (0.074) |
| Couples with dependent children |  | 2.532*** | 2.543*** | 2.533*** | 2.527*** | 2.522*** | 2.536*** | 2.521*** | 2.520*** |
|  |  | (0.221) | (0.224) | (0.222) | (0.223) | (0.222) | (0.219) | (0.223) | (0.226) |
| Couples without dependent children |  | 2.695*** | 2.698*** | 2.698*** | 2.695*** | 2.698*** | 2.694*** | 2.694*** | 2.698*** |
|  |  | (0.11) | (0.11) | (0.11) | (0.11) | (0.11) | (0.11) | (0.111) | (0.111) |
| Employed during the EQ (1/0) |  | 0.882*** | 0.882*** | 0.882*** | 0.882*** | 0.881*** | 0.880*** | 0.877** | 0.878** |
|  |  | (0.0306) | (0.0307) | (0.0307) | (0.0306) | (0.0308) | (0.0305) | (0.0549) | (0.0542) |
| treatment#1.Female |  |  | 0.97 |  |  |  |  |  | 0.982 |
|  |  |  | (0.233) |  |  |  |  |  | (0.245) |
| post_earthquake#Female |  |  | 0.858*** |  |  |  |  |  | 0.845*** |
|  |  |  | (0.05) |  |  |  |  |  | (0.0455) |
| treatment#post_earthquake#Female |  |  | 0.886 |  |  |  |  |  | 0.895 |
|  |  |  | (0.196) |  |  |  |  |  | (0.198) |
| treatment#Maori |  |  |  | 0.615*** |  |  |  |  | 0.648*** |
|  |  |  |  | (0.09) |  |  |  |  | (0.0822) |
| post_earthquake#Maori |  |  |  | 0.967 |  |  |  |  | 0.913 |
|  |  |  |  | (0.0668) |  |  |  |  | (0.0724) |
| treatment#post_earthquake#Maori |  |  |  | 1.893** |  |  |  |  | 1.774** |
|  |  |  |  | (0.539) |  |  |  |  | (0.482) |
| treatment#Sole parents with Dependent Children |  |  |  |  | 0.894 |  |  |  | 1.01 |
|  |  |  |  |  | (0.106) |  |  |  | (0.199) |
| post_earthquake#Sole parents with Dependent Children |  |  |  |  | 1.062 |  |  |  | 1.083 |
|  |  |  |  |  | (0.113) |  |  |  | (0.11) |
| treatment#post_earthquake#Sole parents with Dependent Children |  |  |  |  | 0.782** |  |  |  | 0.728* |
|  |  |  |  |  | (0.0908) |  |  |  | (0.132) |
| treatment#Age (25-35) |  |  |  |  |  | 1.206*** |  |  | 1.190*** |
|  |  |  |  |  |  | (0.0854) |  |  | (0.0631) |
| post_earthquake#Age (25-35) |  |  |  |  |  | 1.251*** |  |  | 1.258*** |
|  |  |  |  |  |  | (0.0973) |  |  | (0.0952) |
| treatment#post_earthquake#Age (25-35) |  |  |  |  |  | 0.635*** |  |  | 0.680*** |
|  |  |  |  |  |  | (0.0609) |  |  | (0.0847) |
| treatment#Migrant |  |  |  |  |  |  | 1.705*** |  | 1.540*** |
|  |  |  |  |  |  |  | (0.163) |  | (0.112) |
| post_earthquake#Migrant |  |  |  |  |  |  | 0.880* |  | 0.858* |
|  |  |  |  |  |  |  | (0.0677) |  | (0.0699) |
| treatment#post_earthquake#Migrant |  |  |  |  |  |  | 0.504*** |  | 0.551*** |
|  |  |  |  |  |  |  | (0.0684) |  | (0.0593) |
| treatment#Employed during EQ |  |  |  |  |  |  |  | 0.561*** | 0.597*** |
|  |  |  |  |  |  |  |  | (0.0529) | (0.0628) |
| post_earthquake#Employed during EQ |  |  |  |  |  |  |  | 1.023 | 1.017 |
|  |  |  |  |  |  |  |  | (0.0826) | (0.0797) |
| treatment#post_earthquake#Employed during EQ |  |  |  |  |  |  |  | 1.530*** | 1.409** |
|  |  |  |  |  |  |  |  | (0.19) | (0.199) |
| Constant | 5.018*** | 4.701*** | 4.419*** | 4.687*** | 4.718*** | 4.848*** | 4.578*** | 4.728*** | 4.395*** |
|  | (0.356) | (0.452) | (0.408) | (0.453) | (0.446) | (0.458) | (0.49) | (0.438) | (0.451) |
| **Observations** | **44,676** | **44,649** | **44,649** | **44,649** | **44,649** | **44,649** | **44,649** | **44,649** | **44,649** |
| *Note: All models are with years fixed effect. Cluster standard errors (at the Territorial Authority level) are in parentheses. *p < 0.1, **p < 0.05, ***p < 0.01* | | | | | | | | | |

**Table 12: Full model - Impact of Earthquake to Income Adequacy (Model 3)**

| **Variables** | **Income Adequacy** | | | | | | | | |  |
| --- | --- | --- | --- | --- | --- | --- | --- | --- | --- | --- |
|  | (1) | (2) | (3) | (4) | (5) | (6) | (7) | (8) | (9) |  |
| treatment | 1.141** | 0.993 | 1.065 | 1.02 | 1.064 | 0.915 | 0.973 | 1.116 | 1.075 |  |
|  | (0.0743) | (0.0779) | (0.111) | (0.0744) | (0.0907) | (0.0596) | (0.079) | (0.0881) | (0.0832) |  |
| treatment * post EQ | 1.098 | 1.079 | 1.098 | 1.056 | 1.045 | 1.237*** | 1.073 | 0.965 | 1.097 |  |
|  | (0.106) | (0.0828) | (0.139) | (0.0755) | (0.0804) | (0.0807) | (0.0853) | (0.0982) | (0.122) |  |
| Age during the EQ (Ref: <25 y.o) |  |  |  |  |  |  |  |  |  |  |
| 25-35 years old |  | 0.931 | 0.93 | 0.931 | 0.933 | 0.807*** | 0.933 | 0.93 | 0.801*** |  |
|  |  | (0.0487) | (0.0488) | (0.0487) | (0.0489) | (0.0471) | (0.068) | (0.0481) | (0.0461) |  |
| 36-55 years old |  | 0.758*** | 0.758*** | 0.758*** | 0.760*** | 0.756*** | 0.759*** | 0.757*** | 0.757*** |  |
|  |  | (0.0341) | (0.0341) | (0.0341) | (0.0341) | (0.0329) | (0.0345) | (0.0335) | (0.0325) |  |
| 56-65 years old |  | 0.747*** | 0.746*** | 0.746*** | 0.748*** | 0.745*** | 0.748*** | 0.745*** | 0.744*** |  |
|  |  | (0.0315) | (0.0316) | (0.0314) | (0.0316) | (0.0312) | (0.0315) | (0.0314) | (0.031) |  |
| >65 years old |  | 1.561*** | 1.559*** | 1.560*** | 1.562*** | 1.553*** | 1.561*** | 1.559*** | 1.549*** |  |
|  |  | (0.082) | (0.0818) | (0.082) | (0.0824) | (0.0828) | (0.0818) | (0.0824) | (0.0834) |  |
| Household size |  | 0.925*** | 0.925*** | 0.925*** | 0.925*** | 0.925*** | 0.925*** | 0.925*** | 0.925*** |  |
|  |  | (0.0107) | (0.0107) | (0.0107) | (0.0107) | (0.0107) | (0.0108) | (0.0107) | (0.0107) |  |
| Dependent children (1/0) |  | 0.801*** | 0.798*** | 0.801*** | 0.805*** | 0.801*** | 0.800*** | 0.804*** | 0.803*** |  |
|  |  | (0.0496) | (0.0498) | (0.0498) | (0.0501) | (0.0499) | (0.049) | (0.0502) | (0.0502) |  |
| Gender (1=Female) |  | 0.930*** | 1.046 | 0.930*** | 0.930*** | 0.931** | 0.930*** | 0.930*** | 1.05 |  |
|  |  | (0.0258) | (0.0393) | (0.0258) | (0.0256) | (0.026) | (0.0258) | (0.0257) | (0.0383) |  |
| European |  | 1.428*** | 1.428*** | 1.430*** | 1.429*** | 1.426*** | 1.441*** | 1.428*** | 1.443*** |  |
|  |  | (0.0759) | (0.0758) | (0.0755) | (0.0757) | (0.0763) | (0.081) | (0.0759) | (0.0806) |  |
| Maori |  | 0.663*** | 0.663*** | 0.685*** | 0.661*** | 0.664*** | 0.664*** | 0.663*** | 0.708*** |  |
|  |  | (0.0312) | (0.0313) | (0.0435) | (0.0314) | (0.0313) | (0.0316) | (0.0312) | (0.0487) |  |
| Pacific |  | 0.436*** | 0.436*** | 0.436*** | 0.436*** | 0.436*** | 0.440*** | 0.436*** | 0.441*** |  |
|  |  | (0.0389) | (0.0389) | (0.0392) | (0.039) | (0.0393) | (0.0378) | (0.0389) | (0.0386) |  |
| Asian |  | 1.089* | 1.088* | 1.090* | 1.091* | 1.087* | 1.107** | 1.091* | 1.105** |  |
|  |  | (0.0521) | (0.0519) | (0.053) | (0.0525) | (0.0525) | (0.05) | (0.052) | (0.0507) |  |
| MELAA |  | 0.765** | 0.763** | 0.765** | 0.765** | 0.765** | 0.781* | 0.766** | 0.781* |  |
|  |  | (0.0985) | (0.0981) | (0.0982) | (0.0984) | (0.0984) | (0.101) | (0.0989) | (0.101) |  |
| Other |  | 0.97 | 0.968 | 0.971 | 0.967 | 0.972 | 0.974 | 0.969 | 0.969 |  |
|  |  | (0.113) | (0.113) | (0.113) | (0.112) | (0.113) | (0.114) | (0.113) | (0.113) |  |
| Migrant to NZ |  | 0.939 | 0.939 | 0.94 | 0.938 | 0.94 | 1.013 | 0.939 | 1.033 |  |
|  |  | (0.0384) | (0.0383) | (0.0384) | (0.0382) | (0.0384) | (0.0599) | (0.0382) | (0.0633) |  |
| Sole parents with dependent children |  | 0.670*** | 0.672*** | 0.669*** | 0.673*** | 0.669*** | 0.671*** | 0.667*** | 0.662*** |  |
|  |  | (0.0491) | (0.0494) | (0.0492) | (0.08) | (0.0495) | (0.0489) | (0.0495) | (0.0763) |  |
| Couples with dependent children |  | 2.600*** | 2.613*** | 2.601*** | 2.587*** | 2.598*** | 2.605*** | 2.590*** | 2.594*** |  |
|  |  | (0.231) | (0.234) | (0.232) | (0.231) | (0.237) | (0.229) | (0.233) | (0.238) |  |
| Couples without dependent children |  | 2.775*** | 2.779*** | 2.776*** | 2.774*** | 2.776*** | 2.774*** | 2.774*** | 2.776*** |  |
|  |  | (0.126) | (0.127) | (0.126) | (0.127) | (0.125) | (0.126) | (0.127) | (0.126) |  |
| Employed during the EQ (1/0) |  | 0.889*** | 0.889*** | 0.889*** | 0.890*** | 0.888*** | 0.888*** | 0.881** | 0.880** |  |
|  |  | (0.0276) | (0.0276) | (0.0276) | (0.0275) | (0.0276) | (0.0276) | (0.0553) | (0.0542) |  |
| treatment#1.Female |  |  | 0.887* |  |  |  |  |  | 0.969 |  |
|  |  |  | (0.062) |  |  |  |  |  | (0.0676) |  |
| post_earthquake#Female |  |  | 0.856*** |  |  |  |  |  | 0.844*** |  |
|  |  |  | (0.0497) |  |  |  |  |  | (0.0453) |  |
| treatment#post_earthquake#Female |  |  | 0.972 |  |  |  |  |  | 0.917 |  |
|  |  |  | (0.0938) |  |  |  |  |  | (0.0845) |  |
| treatment#Maori |  |  |  | 0.763** |  |  |  |  | 0.786* |  |
|  |  |  |  | (0.104) |  |  |  |  | (0.11) |  |
| post_earthquake#Maori |  |  |  | 0.968 |  |  |  |  | 0.914 |  |
|  |  |  |  | (0.0675) |  |  |  |  | (0.0726) |  |
| treatment#post_earthquake#Maori |  |  |  | 1.25 |  |  |  |  | 1.293 |  |
|  |  |  |  | (0.233) |  |  |  |  | (0.233) |  |
| treatment#Sole parents with Dependent Children |  |  |  |  | 0.525*** |  |  |  | 0.512*** |  |
|  |  |  |  |  | (0.0524) |  |  |  | (0.0584) |  |
| post_earthquake#Sole parents with Dependent Children |  |  |  |  | 1.052 |  |  |  | 1.073 |  |
|  |  |  |  |  | (0.112) |  |  |  | (0.109) |  |
| treatment#post_earthquake#Sole parents with Dependent Children |  |  |  |  | 1.412*** |  |  |  | 1.591*** |  |
|  |  |  |  |  | (0.168) |  |  |  | (0.178) |  |
| treatment#Age (25-35) |  |  |  |  |  | 1.816*** |  |  | 1.967*** |  |
|  |  |  |  |  |  | (0.292) |  |  | (0.346) |  |
| post_earthquake#Age (25-35) |  |  |  |  |  | 1.247*** |  |  | 1.254*** |  |
|  |  |  |  |  |  | (0.0973) |  |  | (0.0953) |  |
| treatment#post_earthquake#Age (25-35) |  |  |  |  |  | 0.402*** |  |  | 0.377*** |  |
|  |  |  |  |  |  | (0.0661) |  |  | (0.0663) |  |
| treatment#Migrant |  |  |  |  |  |  | 1.120* |  | 1.03 |  |
|  |  |  |  |  |  |  | (0.071) |  | (0.0687) |  |
| post_earthquake#Migrant |  |  |  |  |  |  | 0.876* |  | 0.853* |  |
|  |  |  |  |  |  |  | (0.068) |  | (0.0699) |  |
| treatment#post_earthquake#Migrant |  |  |  |  |  |  | 0.991 |  | 1.084 |  |
|  |  |  |  |  |  |  | (0.101) |  | (0.118) |  |
| treatment#Employed during EQ |  |  |  |  |  |  |  | 0.866* | 0.932 |  |
|  |  |  |  |  |  |  |  | (0.0751) | (0.0785) |  |
| post_earthquake#Employed during EQ |  |  |  |  |  |  |  | 1.022 | 1.015 |  |
|  |  |  |  |  |  |  |  | (0.0826) | (0.0793) |  |
| treatment#post_earthquake#Employed during EQ |  |  |  |  |  |  |  | 1.148 | 1.097 |  |
|  |  |  |  |  |  |  |  | (0.13) | (0.119) |  |
| Constant | 4.970*** | 4.592*** | 4.288*** | 4.562*** | 4.573*** | 4.726*** | 4.474*** | 4.630*** | 4.260*** |  |
|  | (0.339) | (0.396) | (0.353) | (0.394) | (0.387) | (0.401) | (0.437) | (0.396) | (0.411) |  |
| **Observations** | **48,606** | **48,579** | **48,579** | **48,579** | **48,579** | **48,579** | **48,579** | **48,579** | **48,579** |  |
| *Note: All models are with years fixed effect. Cluster standard errors (at the Territorial Authority level) are in parentheses. *p < 0.1, **p < 0.05, ***p < 0.01* | | | | | | | | | |  |

**Table 13: Full model - Impact of Earthquake to Safety(Model 1)**

| **Variables** | **Safety** | | | | | | | | |
| --- | --- | --- | --- | --- | --- | --- | --- | --- | --- |
|  | (1) | (2) | (3) | (4) | (5) | (6) | (7) | (8) | (9) |
| treatment | 1.08 | 1.085 | 1.544*** | 1.093 | 1.117 | 1.120* | 1.170** | 0.616*** | 0.986 |
|  | (0.0666) | (0.0784) | (0.132) | (0.0781) | (0.0806) | (0.0766) | (0.0856) | (0.0555) | (0.0921) |
| treatment * post EQ | 0.880*** | 0.911* | 0.678*** | 0.905** | 0.899** | 0.909* | 0.832*** | 1.609*** | 1.075 |
|  | (0.0434) | (0.0473) | (0.0533) | (0.0436) | (0.047) | (0.0477) | (0.0424) | (0.131) | (0.0975) |
| Age during the EQ (Ref: <25 y.o) |  |  |  |  |  |  |  |  |  |
| 25-35 years old |  | 1.087** | 1.085** | 1.087** | 1.085** | 1.079 | 1.086** | 1.091** | 1.068 |
|  |  | (0.0424) | (0.0425) | (0.0423) | (0.042) | (0.0655) | (0.0574) | (0.0427) | (0.062) |
| 36-55 years old |  | 1.107*** | 1.106*** | 1.107*** | 1.103** | 1.106*** | 1.106*** | 1.111*** | 1.107*** |
|  |  | (0.0429) | (0.0429) | (0.0426) | (0.0425) | (0.0432) | (0.0428) | (0.0428) | (0.0423) |
| 56-65 years old |  | 0.774*** | 0.773*** | 0.774*** | 0.772*** | 0.773*** | 0.773*** | 0.777*** | 0.774*** |
|  |  | (0.0453) | (0.0454) | (0.045) | (0.0454) | (0.0459) | (0.0453) | (0.045) | (0.0454) |
| >65 years old |  | 0.411*** | 0.411*** | 0.411*** | 0.411*** | 0.411*** | 0.411*** | 0.412*** | 0.411*** |
|  |  | (0.0361) | (0.0362) | (0.0361) | (0.0362) | (0.0363) | (0.036) | (0.0362) | (0.0364) |
| Household size |  | 1.015 | 1.015 | 1.015 | 1.015 | 1.015 | 1.015 | 1.015 | 1.015 |
|  |  | (0.0133) | (0.0133) | (0.0134) | (0.0133) | (0.0133) | (0.0133) | (0.0132) | (0.0133) |
| Dependent children (1/0) |  | 0.948 | 0.947 | 0.948 | 0.948 | 0.948 | 0.95 | 0.942 | 0.944 |
|  |  | (0.0528) | (0.0526) | (0.0528) | (0.053) | (0.053) | (0.0534) | (0.0524) | (0.0531) |
| Gender (1=Female) |  | 0.209*** | 0.234*** | 0.209*** | 0.209*** | 0.209*** | 0.209*** | 0.209*** | 0.232*** |
|  |  | (0.00798) | (0.0155) | (0.00798) | (0.00798) | (0.00798) | (0.008) | (0.00799) | (0.0156) |
| European |  | 1.096 | 1.096 | 1.096 | 1.097 | 1.096 | 1.088 | 1.096 | 1.089 |
|  |  | (0.067) | (0.0668) | (0.067) | (0.0671) | (0.0671) | (0.067) | (0.0671) | (0.067) |
| Maori |  | 1.143** | 1.143** | 1.148 | 1.143** | 1.143** | 1.140** | 1.145** | 1.108 |
|  |  | (0.0672) | (0.0671) | (0.102) | (0.0671) | (0.0673) | (0.0671) | (0.0674) | (0.101) |
| Pacific |  | 0.851** | 0.852** | 0.851** | 0.853** | 0.851** | 0.845*** | 0.851** | 0.849** |
|  |  | (0.0534) | (0.0535) | (0.054) | (0.0534) | (0.0535) | (0.0533) | (0.0537) | (0.0542) |
| Asian |  | 0.849*** | 0.848*** | 0.849*** | 0.849*** | 0.848*** | 0.837*** | 0.847*** | 0.836*** |
|  |  | (0.0531) | (0.0532) | (0.0534) | (0.0532) | (0.0526) | (0.0532) | (0.0528) | (0.0532) |
| MELAA |  | 0.849 | 0.847 | 0.849 | 0.851 | 0.847 | 0.837 | 0.849 | 0.837 |
|  |  | (0.159) | (0.158) | (0.158) | (0.159) | (0.159) | (0.157) | (0.16) | (0.157) |
| Other |  | 1.063 | 1.061 | 1.063 | 1.063 | 1.063 | 1.058 | 1.066 | 1.061 |
|  |  | (0.15) | (0.15) | (0.15) | (0.151) | (0.15) | (0.149) | (0.15) | (0.149) |
| Migrant to NZ |  | 1.278*** | 1.277*** | 1.278*** | 1.278*** | 1.278*** | 1.207*** | 1.277*** | 1.210*** |
|  |  | (0.0453) | (0.0454) | (0.0453) | (0.0453) | (0.0454) | (0.0595) | (0.0456) | (0.0603) |
| Sole parents with dependent children |  | 0.95 | 0.951 | 0.949 | 1.079 | 0.949 | 0.947 | 0.955 | 1.054 |
|  |  | (0.0652) | (0.0653) | (0.0652) | (0.096) | (0.0653) | (0.0655) | (0.0646) | (0.0953) |
| Couples with dependent children |  | 1.116* | 1.118* | 1.116* | 1.118* | 1.115* | 1.114* | 1.126* | 1.125* |
|  |  | (0.0706) | (0.0708) | (0.0703) | (0.071) | (0.0708) | (0.0707) | (0.0705) | (0.0712) |
| Couples without dependent children |  | 1.183*** | 1.183*** | 1.183*** | 1.184*** | 1.183*** | 1.183*** | 1.185*** | 1.186*** |
|  |  | (0.0496) | (0.0493) | (0.0495) | (0.0498) | (0.0498) | (0.0497) | (0.0498) | (0.05) |
| Employed during the EQ (1/0) |  | 0.921** | 0.920** | 0.921** | 0.920** | 0.920** | 0.922** | 0.975 | 0.966 |
|  |  | (0.0336) | (0.0335) | (0.0336) | (0.0336) | (0.0335) | (0.0336) | (0.055) | (0.0548) |
| treatment#1.Female |  |  | 0.617*** |  |  |  |  |  | 0.621*** |
|  |  |  | (0.0413) |  |  |  |  |  | (0.0423) |
| post_earthquake#Female |  |  | 0.850** |  |  |  |  |  | 0.859* |
|  |  |  | (0.0653) |  |  |  |  |  | (0.0669) |
| treatment#post_earthquake#Female |  |  | 1.506*** |  |  |  |  |  | 1.511*** |
|  |  |  | (0.115) |  |  |  |  |  | (0.117) |
| treatment#Maori |  |  |  | 0.847* |  |  |  |  | 0.835* |
|  |  |  |  | (0.0743) |  |  |  |  | (0.0768) |
| post_earthquake#Maori |  |  |  | 0.995 |  |  |  |  | 1.044 |
|  |  |  |  | (0.0907) |  |  |  |  | (0.101) |
| treatment#post_earthquake#Maori |  |  |  | 1.176* |  |  |  |  | 1.222* |
|  |  |  |  | (0.112) |  |  |  |  | (0.126) |
| treatment#Sole parents with Dependent Children |  |  |  |  | 0.600*** |  |  |  | 0.582*** |
|  |  |  |  |  | (0.0457) |  |  |  | (0.0459) |
| post_earthquake#Sole parents with Dependent Children |  |  |  |  | 0.841** |  |  |  | 0.872 |
|  |  |  |  |  | (0.0717) |  |  |  | (0.0802) |
| treatment#post_earthquake#Sole parents with Dependent Children |  |  |  |  | 1.345*** |  |  |  | 1.469*** |
|  |  |  |  |  | (0.117) |  |  |  | (0.141) |
| treatment#Age (25-35) |  |  |  |  |  | 0.832*** |  |  | 0.902* |
|  |  |  |  |  |  | (0.0496) |  |  | (0.0532) |
| post_earthquake#Age (25-35) |  |  |  |  |  | 1.024 |  |  | 1.037 |
|  |  |  |  |  |  | (0.0715) |  |  | (0.0711) |
| treatment#post_earthquake#Age (25-35) |  |  |  |  |  | 0.988 |  |  | 0.91 |
|  |  |  |  |  |  | (0.0684) |  |  | (0.0629) |
| treatment#Migrant |  |  |  |  |  |  | 0.715*** |  | 0.760*** |
|  |  |  |  |  |  |  | (0.0352) |  | (0.0367) |
| post_earthquake#Migrant |  |  |  |  |  |  | 1.093* |  | 1.085 |
|  |  |  |  |  |  |  | (0.0574) |  | (0.0595) |
| treatment#post_earthquake#Migrant |  |  |  |  |  |  | 1.522*** |  | 1.452*** |
|  |  |  |  |  |  |  | (0.0754) |  | (0.0761) |
| treatment#Employed during EQ |  |  |  |  |  |  |  | 2.074*** | 2.047*** |
|  |  |  |  |  |  |  |  | (0.119) | (0.116) |
| post_earthquake#Employed during EQ |  |  |  |  |  |  |  | 0.91 | 0.923 |
|  |  |  |  |  |  |  |  | (0.0598) | (0.0612) |
| treatment#post_earthquake#Employed during EQ |  |  |  |  |  |  |  | 0.481*** | 0.489*** |
|  |  |  |  |  |  |  |  | (0.0316) | (0.0323) |
| Constant | 3.912*** | 11.90*** | 10.94*** | 11.89*** | 11.77*** | 11.92*** | 12.13*** | 11.34*** | 10.73*** |
|  | (0.281) | (1.499) | (1.373) | (1.492) | (1.476) | (1.504) | (1.526) | (1.55) | (1.431) |
| **Observations** | **42,054** | **42,036** | **42,036** | **42,036** | **42,036** | **42,036** | **42,036** | **42,036** | **42,036** |
| *Note: All models are with years fixed effect. Cluster standard errors (at the Territorial Authority level) are in parentheses. *p < 0.1, **p < 0.05, ***p < 0.01* | | | | | | | | | |

**Table 14: Full model - Impact of Earthquake to Safety(Model 2)**

| **Variables** | **Safety** | | | | | | | | |  |
| --- | --- | --- | --- | --- | --- | --- | --- | --- | --- | --- |
|  | (1) | (2) | (3) | (4) | (5) | (6) | (7) | (8) | (9) |  |
| treatment | 1.134 | 1.155 | 1.641*** | 1.165 | 1.186* | 1.226 | 1.228** | 0.683** | 1.113 |  |
|  | (0.0941) | (0.118) | (0.183) | (0.121) | (0.119) | (0.154) | (0.11) | (0.107) | (0.187) |  |
| treatment * post EQ | 0.872** | 0.888* | 0.654*** | 0.884** | 0.873** | 0.865* | 0.850*** | 1.412* | 0.969 |  |
|  | (0.0476) | (0.0553) | (0.0576) | (0.0551) | (0.0555) | (0.0751) | (0.0518) | (0.263) | (0.166) |  |
| Age during the EQ (Ref: <25 y.o) |  |  |  |  |  |  |  |  |  |  |
| 25-35 years old |  | 1.082** | 1.081** | 1.082** | 1.081** | 1.081 | 1.082** | 1.086** | 1.07 |  |
|  |  | (0.0424) | (0.0425) | (0.0423) | (0.042) | (0.0653) | (0.0571) | (0.0427) | (0.0618) |  |
| 36-55 years old |  | 1.107*** | 1.107*** | 1.107*** | 1.104*** | 1.107*** | 1.107*** | 1.112*** | 1.107*** |  |
|  |  | (0.0428) | (0.0428) | (0.0425) | (0.0423) | (0.043) | (0.0426) | (0.0427) | (0.0421) |  |
| 56-65 years old |  | 0.771*** | 0.770*** | 0.771*** | 0.770*** | 0.771*** | 0.771*** | 0.775*** | 0.772*** |  |
|  |  | (0.0452) | (0.0452) | (0.0449) | (0.0452) | (0.0457) | (0.0452) | (0.0449) | (0.0452) |  |
| >65 years old |  | 0.413*** | 0.412*** | 0.413*** | 0.412*** | 0.412*** | 0.413*** | 0.414*** | 0.413*** |  |
|  |  | (0.036) | (0.0361) | (0.036) | (0.0362) | (0.0362) | (0.0359) | (0.0361) | (0.0362) |  |
| Household size |  | 1.016 | 1.016 | 1.016 | 1.017 | 1.016 | 1.017 | 1.016 | 1.016 |  |
|  |  | (0.0132) | (0.0132) | (0.0133) | (0.0133) | (0.0132) | (0.0133) | (0.0132) | (0.0132) |  |
| Dependent children (1/0) |  | 0.944 | 0.943 | 0.944 | 0.944 | 0.944 | 0.946 | 0.938 | 0.939 |  |
|  |  | (0.0525) | (0.0523) | (0.0524) | (0.0526) | (0.0527) | (0.053) | (0.052) | (0.0527) |  |
| Gender (1=Female) |  | 0.209*** | 0.234*** | 0.209*** | 0.209*** | 0.209*** | 0.209*** | 0.209*** | 0.232*** |  |
|  |  | (0.00795) | (0.0155) | (0.00795) | (0.00795) | (0.00795) | (0.00796) | (0.00796) | (0.0156) |  |
| European |  | 1.102 | 1.102 | 1.103 | 1.103 | 1.102 | 1.094 | 1.102 | 1.096 |  |
|  |  | (0.0672) | (0.0669) | (0.0672) | (0.0672) | (0.0673) | (0.0671) | (0.0673) | (0.0672) |  |
| Maori |  | 1.144** | 1.144** | 1.15 | 1.144** | 1.143** | 1.142** | 1.146** | 1.112 |  |
|  |  | (0.0672) | (0.0671) | (0.103) | (0.067) | (0.0672) | (0.067) | (0.0673) | (0.102) |  |
| Pacific |  | 0.853** | 0.854** | 0.854** | 0.855** | 0.853** | 0.847*** | 0.854** | 0.851** |  |
|  |  | (0.0534) | (0.0534) | (0.054) | (0.0533) | (0.0534) | (0.0533) | (0.0536) | (0.0541) |  |
| Asian |  | 0.855** | 0.854** | 0.856** | 0.855** | 0.854** | 0.844*** | 0.853** | 0.843*** |  |
|  |  | (0.0536) | (0.0537) | (0.054) | (0.0538) | (0.0532) | (0.0539) | (0.0533) | (0.0541) |  |
| MELAA |  | 0.854 | 0.851 | 0.854 | 0.855 | 0.851 | 0.843 | 0.853 | 0.842 |  |
|  |  | (0.16) | (0.159) | (0.159) | (0.16) | (0.16) | (0.158) | (0.16) | (0.158) |  |
| Other |  | 1.067 | 1.065 | 1.067 | 1.068 | 1.067 | 1.063 | 1.07 | 1.067 |  |
|  |  | (0.151) | (0.151) | (0.151) | (0.151) | (0.151) | (0.149) | (0.151) | (0.15) |  |
| Migrant to NZ |  | 1.272*** | 1.272*** | 1.273*** | 1.273*** | 1.273*** | 1.208*** | 1.272*** | 1.212*** |  |
|  |  | (0.0454) | (0.0455) | (0.0453) | (0.0454) | (0.0455) | (0.0597) | (0.0457) | (0.0605) |  |
| Sole parents with dependent children |  | 0.954 | 0.955 | 0.954 | 1.083 | 0.954 | 0.952 | 0.961 | 1.059 |  |
|  |  | (0.0654) | (0.0655) | (0.0655) | (0.0963) | (0.0655) | (0.0657) | (0.0648) | (0.0957) |  |
| Couples with dependent children |  | 1.119* | 1.121* | 1.119* | 1.120* | 1.117* | 1.117* | 1.128* | 1.127* |  |
|  |  | (0.0706) | (0.0708) | (0.0703) | (0.071) | (0.0707) | (0.0708) | (0.0704) | (0.0711) |  |
| Couples without dependent children |  | 1.183*** | 1.184*** | 1.183*** | 1.184*** | 1.183*** | 1.184*** | 1.185*** | 1.186*** |  |
|  |  | (0.0495) | (0.0492) | (0.0494) | (0.0497) | (0.0498) | (0.0496) | (0.0497) | (0.0499) |  |
| Employed during the EQ (1/0) |  | 0.922** | 0.922** | 0.922** | 0.922** | 0.922** | 0.923** | 0.972 | 0.964 |  |
|  |  | (0.0335) | (0.0334) | (0.0335) | (0.0336) | (0.0334) | (0.0335) | (0.0548) | (0.0547) |  |
| treatment#1.Female |  |  | 0.620*** |  |  |  |  |  | 0.625*** |  |
|  |  |  | (0.0417) |  |  |  |  |  | (0.0432) |  |
| post_earthquake#Female |  |  | 0.850** |  |  |  |  |  | 0.859* |  |
|  |  |  | (0.0653) |  |  |  |  |  | (0.0669) |  |
| treatment#post_earthquake#Female |  |  | 1.519*** |  |  |  |  |  | 1.514*** |  |
|  |  |  | (0.132) |  |  |  |  |  | (0.132) |  |
| treatment#Maori |  |  |  | 0.860* |  |  |  |  | 0.827** |  |
|  |  |  |  | (0.0741) |  |  |  |  | (0.0772) |  |
| post_earthquake#Maori |  |  |  | 0.995 |  |  |  |  | 1.044 |  |
|  |  |  |  | (0.0909) |  |  |  |  | (0.101) |  |
| treatment#post_earthquake#Maori |  |  |  | 1.109 |  |  |  |  | 1.141 |  |
|  |  |  |  | (0.142) |  |  |  |  | (0.174) |  |
| treatment#Sole parents with Dependent Children |  |  |  |  | 0.640*** |  |  |  | 0.647*** |  |
|  |  |  |  |  | (0.069) |  |  |  | (0.0911) |  |
| post_earthquake#Sole parents with Dependent Children |  |  |  |  | 0.840** |  |  |  | 0.871 |  |
|  |  |  |  |  | (0.0716) |  |  |  | (0.0802) |  |
| treatment#post_earthquake#Sole parents with Dependent Children |  |  |  |  | 1.360*** |  |  |  | 1.418** |  |
|  |  |  |  |  | (0.158) |  |  |  | (0.198) |  |
| treatment#Age (25-35) |  |  |  |  |  | 0.691 |  |  | 0.725 |  |
|  |  |  |  |  |  | (0.162) |  |  | (0.199) |  |
| post_earthquake#Age (25-35) |  |  |  |  |  | 1.021 |  |  | 1.035 |  |
|  |  |  |  |  |  | (0.0711) |  |  | (0.0708) |  |
| treatment#post_earthquake#Age (25-35) |  |  |  |  |  | 1.141 |  |  | 1.102 |  |
|  |  |  |  |  |  | (0.264) |  |  | (0.314) |  |
| treatment#Migrant |  |  |  |  |  |  | 0.742*** |  | 0.796** |  |
|  |  |  |  |  |  |  | (0.0606) |  | (0.0737) |  |
| post_earthquake#Migrant |  |  |  |  |  |  | 1.091* |  | 1.083 |  |
|  |  |  |  |  |  |  | (0.0571) |  | (0.0592) |  |
| treatment#post_earthquake#Migrant |  |  |  |  |  |  | 1.249 |  | 1.187 |  |
|  |  |  |  |  |  |  | (0.293) |  | (0.285) |  |
| treatment#Employed during EQ |  |  |  |  |  |  |  | 1.953*** | 1.928*** |  |
|  |  |  |  |  |  |  |  | (0.192) | (0.181) |  |
| post_earthquake#Employed during EQ |  |  |  |  |  |  |  | 0.912 | 0.924 |  |
|  |  |  |  |  |  |  |  | (0.0599) | (0.0613) |  |
| treatment#post_earthquake#Employed during EQ |  |  |  |  |  |  |  | 0.556*** | 0.553*** |  |
|  |  |  |  |  |  |  |  | (0.109) | (0.0982) |  |
| Constant | 3.918*** | 11.85*** | 10.89*** | 11.83*** | 11.72*** | 11.86*** | 12.06*** | 11.33*** | 10.68*** |  |
|  | (0.282) | (1.486) | (1.361) | (1.477) | (1.463) | (1.489) | (1.51) | (1.547) | (1.42) |  |
| **Observations** | **42,294** | **42,276** | **42,276** | **42,276** | **42,276** | **42,276** | **42,276** | **42,276** | **42,276** |  |
| *Note: All models are with years fixed effect. Cluster standard errors (at the Territorial Authority level) are in parentheses. *p < 0.1, **p < 0.05, ***p < 0.01* | | | | | | | | | |  |

**Table 15: Full model - Impact of Earthquake to Safety(Model 3)**

| **Variables** | **Safety** | | | | | | | | |
| --- | --- | --- | --- | --- | --- | --- | --- | --- | --- |
|  | (1) | (2) | (3) | (4) | (5) | (6) | (7) | (8) | (9) |
| treatment | 1.033 | 0.985 | 1.181 | 0.987 | 1.024 | 1.037 | 0.999 | 1.012 | 1.288** |
|  | (0.108) | (0.119) | (0.137) | (0.121) | (0.119) | (0.132) | (0.112) | (0.133) | (0.164) |
| treatment * post EQ | 0.993 | 1.035 | 0.884 | 1.054 | 0.979 | 1.009 | 1.045 | 0.892 | 0.781 |
|  | (0.0629) | (0.066) | (0.076) | (0.0677) | (0.0611) | (0.0731) | (0.0687) | (0.11) | (0.128) |
| Age during the EQ (Ref: <25 y.o) |  |  |  |  |  |  |  |  |  |
| 25-35 years old |  | 1.076* | 1.074* | 1.075* | 1.075* | 1.093 | 1.075* | 1.079* | 1.081 |
|  |  | (0.0451) | (0.0449) | (0.0453) | (0.0444) | (0.0639) | (0.057) | (0.0459) | (0.0603) |
| 36-55 years old |  | 1.116*** | 1.115*** | 1.115*** | 1.115*** | 1.115*** | 1.115*** | 1.119*** | 1.115*** |
|  |  | (0.0392) | (0.0391) | (0.039) | (0.0386) | (0.0393) | (0.0391) | (0.0394) | (0.0384) |
| 56-65 years old |  | 0.788*** | 0.787*** | 0.787*** | 0.787*** | 0.787*** | 0.787*** | 0.790*** | 0.786*** |
|  |  | (0.039) | (0.039) | (0.0388) | (0.0389) | (0.0394) | (0.0389) | (0.039) | (0.0392) |
| >65 years old |  | 0.420*** | 0.420*** | 0.420*** | 0.420*** | 0.419*** | 0.420*** | 0.421*** | 0.419*** |
|  |  | (0.0335) | (0.0336) | (0.0335) | (0.0336) | (0.0335) | (0.0334) | (0.0336) | (0.0335) |
| Household size |  | 1.016 | 1.016 | 1.016 | 1.017 | 1.016 | 1.017 | 1.016 | 1.016 |
|  |  | (0.0122) | (0.0122) | (0.0123) | (0.0123) | (0.0122) | (0.0122) | (0.0122) | (0.0122) |
| Dependent children (1/0) |  | 0.944 | 0.943 | 0.943 | 0.944 | 0.945 | 0.945 | 0.941 | 0.94 |
|  |  | (0.0482) | (0.048) | (0.0478) | (0.0484) | (0.0486) | (0.0483) | (0.0483) | (0.0487) |
| Gender (1=Female) |  | 0.207*** | 0.233*** | 0.207*** | 0.207*** | 0.207*** | 0.207*** | 0.207*** | 0.232*** |
|  |  | (0.00744) | (0.0156) | (0.00745) | (0.00743) | (0.00745) | (0.00745) | (0.00744) | (0.0157) |
| European |  | 1.106* | 1.106* | 1.111* | 1.106* | 1.107* | 1.099 | 1.106* | 1.105* |
|  |  | (0.0641) | (0.0639) | (0.0646) | (0.0641) | (0.0644) | (0.064) | (0.0644) | (0.0645) |
| Maori |  | 1.136** | 1.136** | 1.161* | 1.136** | 1.136** | 1.135** | 1.137** | 1.123 |
|  |  | (0.0632) | (0.0631) | (0.104) | (0.0632) | (0.0633) | (0.0632) | (0.0632) | (0.103) |
| Pacific |  | 0.857*** | 0.858** | 0.860** | 0.856*** | 0.856*** | 0.849*** | 0.857*** | 0.855*** |
|  |  | (0.051) | (0.0511) | (0.052) | (0.0509) | (0.051) | (0.051) | (0.0512) | (0.0519) |
| Asian |  | 0.850*** | 0.850*** | 0.853*** | 0.850*** | 0.849*** | 0.840*** | 0.849*** | 0.840*** |
|  |  | (0.048) | (0.0482) | (0.0489) | (0.0481) | (0.0476) | (0.0486) | (0.048) | (0.0492) |
| MELAA |  | 0.897 | 0.895 | 0.898 | 0.897 | 0.895 | 0.885 | 0.897 | 0.887 |
|  |  | (0.161) | (0.161) | (0.161) | (0.161) | (0.161) | (0.158) | (0.162) | (0.16) |
| Other |  | 1.057 | 1.054 | 1.061 | 1.057 | 1.058 | 1.052 | 1.058 | 1.059 |
|  |  | (0.141) | (0.141) | (0.141) | (0.141) | (0.141) | (0.14) | (0.141) | (0.14) |
| Migrant to NZ |  | 1.266*** | 1.266*** | 1.268*** | 1.266*** | 1.266*** | 1.205*** | 1.266*** | 1.212*** |
|  |  | (0.043) | (0.043) | (0.0427) | (0.043) | (0.0431) | (0.0584) | (0.0431) | (0.0593) |
| Sole parents with dependent children |  | 0.975 | 0.976 | 0.976 | 1.1 | 0.973 | 0.973 | 0.979 | 1.066 |
|  |  | (0.0581) | (0.0582) | (0.0581) | (0.0948) | (0.0583) | (0.0582) | (0.058) | (0.0943) |
| Couples with dependent children |  | 1.129** | 1.132** | 1.131** | 1.130** | 1.127** | 1.128** | 1.135** | 1.134** |
|  |  | (0.064) | (0.0644) | (0.0638) | (0.0645) | (0.0642) | (0.0641) | (0.0642) | (0.0651) |
| Couples without dependent children |  | 1.195*** | 1.195*** | 1.194*** | 1.195*** | 1.195*** | 1.195*** | 1.195*** | 1.196*** |
|  |  | (0.0487) | (0.0485) | (0.0485) | (0.0487) | (0.0493) | (0.0489) | (0.0485) | (0.0488) |
| Employed during the EQ (1/0) |  | 0.918*** | 0.918*** | 0.918*** | 0.919*** | 0.918*** | 0.919** | 0.965 | 0.958 |
|  |  | (0.03) | (0.03) | (0.03) | (0.0301) | (0.0299) | (0.0301) | (0.0546) | (0.0547) |
| treatment#1.Female |  |  | 0.771*** |  |  |  |  |  | 0.799*** |
|  |  |  | (0.0597) |  |  |  |  |  | (0.0606) |
| post_earthquake#Female |  |  | 0.849** |  |  |  |  |  | 0.858* |
|  |  |  | (0.0654) |  |  |  |  |  | (0.0671) |
| treatment#post_earthquake#Female |  |  | 1.256* |  |  |  |  |  | 1.186 |
|  |  |  | (0.151) |  |  |  |  |  | (0.14) |
| treatment#Maori |  |  |  | 0.997 |  |  |  |  | 1.05 |
|  |  |  |  | (0.0954) |  |  |  |  | (0.101) |
| post_earthquake#Maori |  |  |  | 0.992 |  |  |  |  | 1.042 |
|  |  |  |  | (0.0918) |  |  |  |  | (0.102) |
| treatment#post_earthquake#Maori |  |  |  | 0.771** |  |  |  |  | 0.693*** |
|  |  |  |  | (0.0874) |  |  |  |  | (0.0813) |
| treatment#Sole parents with Dependent Children |  |  |  |  | 0.571*** |  |  |  | 0.609*** |
|  |  |  |  |  | (0.0658) |  |  |  | (0.0698) |
| post_earthquake#Sole parents with Dependent Children |  |  |  |  | 0.840** |  |  |  | 0.871 |
|  |  |  |  |  | (0.0717) |  |  |  | (0.0806) |
| treatment#post_earthquake#Sole parents with Dependent Children |  |  |  |  | 2.198*** |  |  |  | 2.212*** |
|  |  |  |  |  | (0.231) |  |  |  | (0.255) |
| treatment#Age (25-35) |  |  |  |  |  | 0.711*** |  |  | 0.731*** |
|  |  |  |  |  |  | (0.0719) |  |  | (0.0746) |
| post_earthquake#Age (25-35) |  |  |  |  |  | 1.019 |  |  | 1.033 |
|  |  |  |  |  |  | (0.0713) |  |  | (0.0709) |
| treatment#post_earthquake#Age (25-35) |  |  |  |  |  | 1.178 |  |  | 1.131 |
|  |  |  |  |  |  | (0.15) |  |  | (0.157) |
| treatment#Migrant |  |  |  |  |  |  | 0.933 |  | 0.911 |
|  |  |  |  |  |  |  | (0.0779) |  | (0.0851) |
| post_earthquake#Migrant |  |  |  |  |  |  | 1.096* |  | 1.087 |
|  |  |  |  |  |  |  | (0.057) |  | (0.059) |
| treatment#post_earthquake#Migrant |  |  |  |  |  |  | 0.969 |  | 1.011 |
|  |  |  |  |  |  |  | (0.175) |  | (0.201) |
| treatment#Employed during EQ |  |  |  |  |  |  |  | 0.967 | 0.98 |
|  |  |  |  |  |  |  |  | (0.114) | (0.117) |
| post_earthquake#Employed during EQ |  |  |  |  |  |  |  | 0.914 | 0.926 |
|  |  |  |  |  |  |  |  | (0.0603) | (0.0619) |
| treatment#post_earthquake#Employed during EQ |  |  |  |  |  |  |  | 1.218* | 1.175 |
|  |  |  |  |  |  |  |  | (0.135) | (0.144) |
| Constant | 3.922*** | 11.85*** | 10.85*** | 11.79*** | 11.73*** | 11.84*** | 12.06*** | 11.36*** | 10.58*** |
|  | (0.274) | (1.425) | (1.31) | (1.407) | (1.403) | (1.421) | (1.446) | (1.506) | (1.366) |
| **Observations** | **46,083** | **46,065** | **46,065** | **46,065** | **46,065** | **46,065** | **46,065** | **46,065** | **46,065** |
| *Note: All models are with years fixed effect. Cluster standard errors (at the Territorial Authority level) are in parentheses. *p < 0.1, **p < 0.05, ***p < 0.01* | | | | | | | | | |

**Table 16: ologit – Impact of Earthquake to Life Satisfaction(Model 1)**

| **Variables** | **Life Satisfaction** | | | | | | | | |
| --- | --- | --- | --- | --- | --- | --- | --- | --- | --- |
|  | (1) | (2) | (3) | (4) | (5) | (6) | (7) | (8) | (9) |
| treatment | 0.932** | 0.925** | 0.900*** | 0.900*** | 0.916** | 0.951 | 0.931** | 0.902** | 0.880** |
|  | (0.0333) | (0.0306) | (0.0292) | (0.0293) | (0.0319) | (0.0331) | (0.0323) | (0.0447) | (0.0481) |
| treatment#post_EQ | 0.904*** | 0.903*** | 0.937** | 0.957 | 0.930** | 0.885*** | 0.899*** | 0.811*** | 0.887** |
|  | (0.0305) | (0.0272) | (0.0293) | (0.0282) | (0.0291) | (0.0302) | (0.029) | (0.0405) | (0.0523) |
| treatment#post_EQ# |  |  |  |  |  |  |  |  |  |
| Female |  |  | 0.935** |  |  |  |  |  | 0.966 |
|  |  |  | (0.0312) |  |  |  |  |  | (0.034) |
| Maori |  |  |  | 0.429*** |  |  |  |  | 0.434*** |
|  |  |  |  | (0.0267) |  |  |  |  | (0.0295) |
| Sole_Dependent_Children |  |  |  |  | 0.564*** |  |  |  | 0.597*** |
|  |  |  |  |  | (0.0515) |  |  |  | (0.0597) |
| Age(25-35) |  |  |  |  |  | 1.134** |  |  | 1.189*** |
|  |  |  |  |  |  | (0.0632) |  |  | (0.0677) |
| Migrant |  |  |  |  |  |  | 1.028 |  | 0.96 |
|  |  |  |  |  |  |  | (0.0487) |  | (0.0474) |
| Employed_During_Earthquake |  |  |  |  |  |  |  | 1.158*** | 1.154*** |
|  |  |  |  |  |  |  |  | (0.0511) | (0.0498) |
| Control | N | Y | Y | Y | Y | Y | Y | Y | Y |
| **Observations** | **45,345** | **45,312** | **45,312** | **45,312** | **45,312** | **45,312** | **45,312** | **45,312** | **45,312** |
| *Note: All models are with years fixed effect. Cluster standard errors (at the Territorial Authority level) are in parentheses. *p < 0.1, **p < 0.05, ***p < 0.01* | | | | | | | | | |

**Table 17: ologit – Impact of Earthquake to General Health(Model 1)**

| **Variables** | **General Health** | | | | | | | | |
| --- | --- | --- | --- | --- | --- | --- | --- | --- | --- |
|  | (1) | (2) | (3) | (4) | (5) | (6) | (7) | (8) | (9) |
| treatment | 1.132*** | 1.081** | 1.178*** | 1.04 | 1.115*** | 1.081** | 1.019 | 1.371*** | 1.280*** |
|  | (0.0436) | (0.0384) | (0.054) | (0.0382) | (0.04) | (0.0386) | (0.0434) | (0.0642) | (0.0801) |
| treatment#post_EQ | 0.874** | 0.880*** | 0.838*** | 0.939 | 0.853*** | 0.874*** | 0.924 | 0.616*** | 0.690*** |
|  | (0.0473) | (0.0394) | (0.0453) | (0.0444) | (0.0371) | (0.0399) | (0.0483) | (0.0416) | (0.06) |
| treatment#post_EQ# |  |  |  |  |  |  |  |  |  |
| Female |  |  | 1.095** |  |  |  |  |  | 1.033 |
|  |  |  | (0.0426) |  |  |  |  |  | (0.0384) |
| Maori |  |  |  | 0.311*** |  |  |  |  | 0.297*** |
|  |  |  |  | (0.0157) |  |  |  |  | (0.016) |
| Sole_Dependent_Children |  |  |  |  | 2.702*** |  |  |  | 2.493*** |
|  |  |  |  |  | (0.211) |  |  |  | (0.197) |
| Age(25-35) |  |  |  |  |  | 1.057 |  |  | 1.05 |
|  |  |  |  |  |  | (0.0642) |  |  | (0.0625) |
| Migrant |  |  |  |  |  |  | 0.814*** |  | 0.807*** |
|  |  |  |  |  |  |  | (0.0401) |  | (0.0428) |
| Employed_During_Earthquake |  |  |  |  |  |  |  | 1.593*** | 1.497*** |
|  |  |  |  |  |  |  |  | (0.075) | (0.0724) |
| Control | N | Y | Y | Y | Y | Y | Y | Y | Y |
| **Observations** | **45,381** | **45,348** | **45,348** | **45,348** | **45,348** | **45,348** | **45,348** | **45,348** | **45,348** |
| *Note: All models are with years fixed effect. Cluster standard errors (at the Territorial Authority level) are in parentheses. *p < 0.1, **p < 0.05, ***p < 0.01* | | | | | | | | | |

**Table 18: ologit – Impact of Earthquake to Income adequacy(Model 1)**

| **Variables** | **Income Adequacy** | | | | | | | | |
| --- | --- | --- | --- | --- | --- | --- | --- | --- | --- |
|  | (1) | (2) | (3) | (4) | (5) | (6) | (7) | (8) | (9) |
| treatment | 1.387*** | 1.275*** | 1.200*** | 1.255*** | 1.286*** | 1.346*** | 1.246*** | 1.539*** | 1.431*** |
|  | (0.0766) | (0.0649) | (0.0679) | (0.0649) | (0.0678) | (0.0638) | (0.0646) | (0.0744) | (0.081) |
| treatment#post_EQ | 0.92 | 0.926** | 1.002 | 0.96 | 0.937* | 0.912*** | 0.927** | 0.719*** | 0.819*** |
|  | (0.0558) | (0.0299) | (0.0413) | (0.0291) | (0.0315) | (0.0282) | (0.0295) | (0.0292) | (0.0344) |
| treatment#post_EQ# |  |  |  |  |  |  |  |  |  |
| Female |  |  | 0.868*** |  |  |  |  |  | 0.889*** |
|  |  |  | (0.0324) |  |  |  |  |  | (0.0335) |
| Maori |  |  |  | 0.541*** |  |  |  |  | 0.553*** |
|  |  |  |  | (0.0369) |  |  |  |  | (0.0481) |
| Sole_Dependent_Children |  |  |  |  | 0.89 |  |  |  | 0.844** |
|  |  |  |  |  | (0.0661) |  |  |  | (0.0622) |
| Age(25-35) |  |  |  |  |  | 1.026 |  |  | 1.03 |
|  |  |  |  |  |  | (0.0557) |  |  | (0.0551) |
| Migrant |  |  |  |  |  |  | 0.988 |  | 0.979 |
|  |  |  |  |  |  |  | (0.0823) |  | (0.0902) |
| Employed_During_Earthquake |  |  |  |  |  |  |  | 1.385*** | 1.334*** |
|  |  |  |  |  |  |  |  | (0.0546) | (0.0499) |
| Control | N | Y | Y | Y | Y | Y | Y | Y | Y |
| **Observations** | **44,427** | **44,403** | **44,403** | **44,400** | **44,403** | **44,403** | **44,400** | **44,403** | **44,403** |
| *Note: All models are with years fixed effect. Cluster standard errors (at the Territorial Authority level) are in parentheses. *p < 0.1, **p < 0.05, ***p < 0.01* | | | | | | | | | |

**Table 19: ologit – Impact of Earthquake to Safety(Model 1)**

| **Variables** | **Safety** | | | | | | | | |
| --- | --- | --- | --- | --- | --- | --- | --- | --- | --- |
|  | (1) | (2) | (3) | (4) | (5) | (6) | (7) | (8) | (9) |
| treatment | 0.965 | 0.953 | 1.014 | 0.966 | 0.965 | 0.99 | 0.996 | 0.632*** | 0.748*** |
|  | (0.0592) | (0.0646) | (0.0754) | (0.0649) | (0.0662) | (0.0657) | (0.0677) | (0.0553) | (0.0678) |
| treatment#post_EQ | 0.94 | 0.986 | 0.978 | 0.979 | 0.973 | 0.969 | 0.933 | 1.561*** | 1.419*** |
|  | (0.0381) | (0.0411) | (0.0559) | (0.0382) | (0.0418) | (0.0424) | (0.04) | (0.11) | (0.113) |
| treatment#post_EQ# |  |  |  |  |  |  |  |  |  |
| Female |  |  | 1.018 |  |  |  |  |  | 1.006 |
|  |  |  | (0.0633) |  |  |  |  |  | (0.0637) |
| Maori |  |  |  | 1.244*** |  |  |  |  | 1.316*** |
|  |  |  |  | (0.0988) |  |  |  |  | (0.107) |
| Sole_Dependent_Children |  |  |  |  | 1.367*** |  |  |  | 1.521*** |
|  |  |  |  |  | (0.0806) |  |  |  | (0.0927) |
| Age(25-35) |  |  |  |  |  | 1.081 |  |  | 1.012 |
|  |  |  |  |  |  | (0.0532) |  |  | (0.0493) |
| Migrant |  |  |  |  |  |  | 1.284*** |  | 1.264*** |
|  |  |  |  |  |  |  | (0.0488) |  | (0.0475) |
| Employed_During_Earthquake |  |  |  |  |  |  |  | 0.557*** | 0.565*** |
|  |  |  |  |  |  |  |  | (0.0297) | (0.0301) |
| Control | N | Y | Y | Y | Y | Y | Y | Y | Y |
| **Observations** | **42,054** | **42,036** | **42,036** | **42,036** | **42,036** | **42,033** | **42,036** | **42,033** | **42,036** |
| *Note: All models are with years fixed effect. Cluster standard errors (at the Territorial Authority level) are in parentheses. *p < 0.1, **p < 0.05, ***p < 0.01* | | | | | | | | | |

**Table 20: ologit – Impact of Earthquake to Life Satisfaction(Model 2)**

| **Variables** | **Life Satisfaction** | | | | | | | | |
| --- | --- | --- | --- | --- | --- | --- | --- | --- | --- |
|  | (1) | (2) | (3) | (4) | (5) | (6) | (7) | (8) | (9) |
| treatment | 0.969 | 0.947 | 0.973 | 0.923* | 0.94 | 0.973 | 0.942 | 0.916* | 0.935 |
|  | (0.0566) | (0.04) | (0.0948) | (0.0399) | (0.0428) | (0.0424) | (0.0348) | (0.0476) | (0.0802) |
| treatment#post_EQ | 0.891*** | 0.890*** | 0.899 | 0.937 | 0.910** | 0.874*** | 0.914** | 0.857* | 0.948 |
|  | (0.0392) | (0.0318) | (0.0668) | (0.0392) | (0.0366) | (0.0333) | (0.0344) | (0.0707) | (0.101) |
| treatment#post_EQ# |  |  |  |  |  |  |  |  |  |
| Female |  |  | 0.983 |  |  |  |  |  | 1.002 |
|  |  |  | (0.091) |  |  |  |  |  | (0.0836) |
| Maori |  |  |  | 0.502*** |  |  |  |  | 0.486*** |
|  |  |  |  | (0.112) |  |  |  |  | (0.0892) |
| Sole_Dependent_Children |  |  |  |  | 0.662** |  |  |  | 0.681** |
|  |  |  |  |  | (0.129) |  |  |  | (0.131) |
| Age(25-35) |  |  |  |  |  | 1.126** |  |  | 1.185*** |
|  |  |  |  |  |  | (0.0651) |  |  | (0.0677) |
| Migrant |  |  |  |  |  |  | 0.894 |  | 0.83 |
|  |  |  |  |  |  |  | (0.158) |  | (0.148) |
| Employed_During_Earthquake |  |  |  |  |  |  |  | 1.056 | 1.04 |
|  |  |  |  |  |  |  |  | (0.12) | (0.131) |
| Control | N | Y | Y | Y | Y | Y | Y | Y | Y |
| **Observations** | **45,600** | **45,564** | **45,564** | **45,564** | **45,564** | **45,564** | **45,564** | **45,564** | **45,564** |
| *Note: All models are with years fixed effect. Cluster standard errors (at the Territorial Authority level) are in parentheses. *p < 0.1, **p < 0.05, ***p < 0.01* | | | | | | | | | |

**Table 21: ologit – Impact of Earthquake to General Health (Model 2)**

| **Variables** | **General Health** | | | | | | | | |
| --- | --- | --- | --- | --- | --- | --- | --- | --- | --- |
|  | (1) | (2) | (3) | (4) | (5) | (6) | (7) | (8) | (9) |
| treatment | 1.107** | 1.065 | 1.175*** | 1.028 | 1.087* | 1.058 | 0.973 | 1.279** | 1.157 |
|  | (0.0541) | (0.0432) | (0.0537) | (0.0414) | (0.0533) | (0.0474) | (0.0705) | (0.149) | (0.195) |
| treatment#post_EQ | 0.914 | 0.916 | 0.845*** | 0.965 | 0.897 | 0.915 | 0.997 | 0.683** | 0.768 |
|  | (0.0678) | (0.0581) | (0.0465) | (0.0541) | (0.0642) | (0.062) | (0.102) | (0.104) | (0.14) |
| treatment#post_EQ# |  |  |  |  |  |  |  |  |  |
| Female |  |  | 1.158** |  |  |  |  |  | 1.116 |
|  |  |  | (0.0832) |  |  |  |  |  | (0.0976) |
| Maori |  |  |  | 0.424** |  |  |  |  | 0.383*** |
|  |  |  |  | (0.15) |  |  |  |  | (0.113) |
| Sole_Dependent_Children |  |  |  |  | 1.965 |  |  |  | 1.779 |
|  |  |  |  |  | (0.807) |  |  |  | (0.733) |
| Age(25-35) |  |  |  |  |  | 1.018 |  |  | 1.042 |
|  |  |  |  |  |  | (0.0742) |  |  | (0.0662) |
| Migrant |  |  |  |  |  |  | 0.688* |  | 0.673 |
|  |  |  |  |  |  |  | (0.156) |  | (0.165) |
| Employed_During_Earthquake |  |  |  |  |  |  |  | 1.465*** | 1.365** |
|  |  |  |  |  |  |  |  | (0.19) | (0.199) |
| Control | N | Y | Y | Y | Y | Y | Y | Y | Y |
| **Observations** | **45,636** | **45,600** | **45,600** | **45,600** | **45,600** | **45,600** | **45,600** | **45,600** | **45,600** |
| *Note: All models are with years fixed effect. Cluster standard errors (at the Territorial Authority level) are in parentheses. *p < 0.1, **p < 0.05, ***p < 0.01* | | | | | | | | | |

**Table 22: ologit – Impact of Earthquake to Income adequacy (Model 2)**

| **Variables** | **Income Adequacy** | | | | | | | | |
| --- | --- | --- | --- | --- | --- | --- | --- | --- | --- |
|  | (1) | (2) | (3) | (4) | (5) | (6) | (7) | (8) | (9) |
| treatment | 1.318*** | 1.194* | 1.193*** | 1.176 | 1.204* | 1.247** | 1.144 | 1.463*** | 1.390*** |
|  | (0.114) | (0.118) | (0.0795) | (0.117) | (0.121) | (0.136) | (0.138) | (0.141) | (0.152) |
| treatment#post_EQ | 0.981 | 0.977 | 0.984 | 1.003 | 0.985 | 0.973 | 1.001 | 0.760*** | 0.824*** |
|  | (0.0925) | (0.0728) | (0.0457) | (0.0626) | (0.0703) | (0.0813) | (0.0981) | (0.0658) | (0.0492) |
| treatment#post_EQ# |  |  |  |  |  |  |  |  |  |
| Female |  |  | 0.989 |  |  |  |  |  | 1.003 |
|  |  |  | (0.149) |  |  |  |  |  | (0.142) |
| Maori |  |  |  | 0.665* |  |  |  |  | 0.674* |
|  |  |  |  | (0.156) |  |  |  |  | (0.149) |
| Sole_Dependent_Children |  |  |  |  | 0.92 |  |  |  | 0.859* |
|  |  |  |  |  | (0.0779) |  |  |  | (0.0674) |
| Age(25-35) |  |  |  |  |  | 0.984 |  |  | 0.997 |
|  |  |  |  |  |  | (0.0679) |  |  | (0.0638) |
| Migrant |  |  |  |  |  |  | 0.891 |  | 0.901 |
|  |  |  |  |  |  |  | (0.137) |  | (0.129) |
| Employed_During_Earthquake |  |  |  |  |  |  |  | 1.380*** | 1.319*** |
|  |  |  |  |  |  |  |  | (0.0606) | (0.0637) |
| Control | N | Y | Y | Y | Y | Y | Y | Y | Y |
| **Observations** | **44,676** | **44,649** | **44,649** | **44,649** | **44,649** | **44,649** | **44,649** | **44,649** | **44,649** |
| *Note: All models are with years fixed effect. Cluster standard errors (at the Territorial Authority level) are in parentheses. *p < 0.1, **p < 0.05, ***p < 0.01* | | | | | | | | | |

**Table 23: ologit – Impact of Earthquake to Safety(Model 2)**

| **Variables** | **Safety** | | | | | | | | |
| --- | --- | --- | --- | --- | --- | --- | --- | --- | --- |
|  | (1) | (2) | (3) | (4) | (5) | (6) | (7) | (8) | (9) |
| treatment | 0.989 | 1.001 | 1.069 | 1.016 | 1.012 | 1.055 | 1.024 | 0.691*** | 0.813 |
|  | (0.0662) | (0.0858) | (0.101) | (0.0879) | (0.0865) | (0.1) | (0.0754) | (0.0918) | (0.102) |
| treatment#post_EQ | 0.968 | 0.99 | 0.957 | 0.978 | 0.975 | 0.961 | 0.975 | 1.484*** | 1.357*** |
|  | (0.0526) | (0.0469) | (0.0611) | (0.0481) | (0.0446) | (0.0516) | (0.0648) | (0.141) | (0.133) |
| treatment#post_EQ# |  |  |  |  |  |  |  |  |  |
| Female |  |  | 1.063 |  |  |  |  |  | 1.046 |
|  |  |  | (0.0888) |  |  |  |  |  | (0.0839) |
| Maori |  |  |  | 1.257** |  |  |  |  | 1.271** |
|  |  |  |  | (0.144) |  |  |  |  | (0.135) |
| Sole_Dependent_Children |  |  |  |  | 1.372** |  |  |  | 1.451** |
|  |  |  |  |  | (0.199) |  |  |  | (0.233) |
| Age(25-35) |  |  |  |  |  | 1.18 |  |  | 1.139 |
|  |  |  |  |  |  | (0.155) |  |  | (0.184) |
| Migrant |  |  |  |  |  |  | 1.09 |  | 1.074 |
|  |  |  |  |  |  |  | (0.214) |  | (0.212) |
| Employed_During_Earthquake |  |  |  |  |  |  |  | 0.600*** | 0.599*** |
|  |  |  |  |  |  |  |  | (0.0588) | (0.0493) |
| Control | N | Y | Y | Y | Y | Y | Y | Y | Y |
| **Observations** | **42,294** | **42,273** | **42,276** | **42,276** | **42,276** | **42,273** | **42,276** | **42,276** | **42,276** |
| *Note: All models are with years fixed effect. Cluster standard errors (at the Territorial Authority level) are in parentheses. *p < 0.1, **p < 0.05, ***p < 0.01* | | | | | | | | | |

**Table 24: ologit – Impact of Earthquake to Life Satisfaction (Model 3)**

| **Variables** | **Life Satisfaction** | | | | | | | | |
| --- | --- | --- | --- | --- | --- | --- | --- | --- | --- |
|  | (1) | (2) | (3) | (4) | (5) | (6) | (7) | (8) | (9) |
| treatment | 0.993 | 0.978 | 0.991 | 0.963 | 0.988 | 0.987 | 0.946 | 0.976 | 0.931 |
|  | (0.0847) | (0.0637) | (0.0746) | (0.0611) | (0.0676) | (0.0682) | (0.055) | (0.111) | (0.107) |
| treatment#post_EQ | 0.9 | 0.895* | 0.911 | 0.91 | 0.893* | 0.885* | 0.889*** | 0.943 | 0.959 |
|  | (0.0626) | (0.0528) | (0.0628) | (0.0552) | (0.0588) | (0.0582) | (0.0335) | (0.0955) | (0.0747) |
| treatment#post_EQ# |  |  |  |  |  |  |  |  |  |
| Female |  |  | 0.968 |  |  |  |  |  | 0.966 |
|  |  |  | (0.0404) |  |  |  |  |  | (0.0403) |
| Maori |  |  |  | 0.868 |  |  |  |  | 0.859 |
|  |  |  |  | (0.08) |  |  |  |  | (0.0816) |
| Sole_Dependent_Children |  |  |  |  | 1.091 |  |  |  | 1.129 |
|  |  |  |  |  | (0.178) |  |  |  | (0.167) |
| Age(25-35) |  |  |  |  |  | 1.07 |  |  | 1.062 |
|  |  |  |  |  |  | (0.0684) |  |  | (0.0713) |
| Migrant |  |  |  |  |  |  | 1.026 |  | 0.993 |
|  |  |  |  |  |  |  | (0.145) |  | (0.141) |
| Employed_During_Earthquake |  |  |  |  |  |  |  | 0.932 | 0.943 |
|  |  |  |  |  |  |  |  | (0.0702) | (0.0603) |
| Control | N | Y | Y | Y | Y | Y | Y | Y | Y |
| **Observations** | **49,608** | **49,572** | **49,569** | **49,572** | **49,572** | **49,572** | **49,572** | **49,569** | **49,569** |
| *Note: All models are with years fixed effect. Cluster standard errors (at the Territorial Authority level) are in parentheses. *p < 0.1, **p < 0.05, ***p < 0.01* | | | | | | | | | |

**Table 25: ologit – Impact of Earthquake to General Health (Model 3)**

| **Variables** | **General Health** | | | | | | | | |
| --- | --- | --- | --- | --- | --- | --- | --- | --- | --- |
|  | (1) | (2) | (3) | (4) | (5) | (6) | (7) | (8) | (9) |
| treatment | 0.964 | 0.928** | 0.959 | 0.915** | 0.935* | 0.915** | 0.891*** | 1.015 | 0.957 |
|  | (0.0407) | (0.035) | (0.0416) | (0.0359) | (0.033) | (0.0388) | (0.0393) | (0.0531) | (0.062) |
| treatment#post_EQ | 1.044 | 1.024 | 0.976 | 1.043 | 1.019 | 1.028 | 1.046 | 0.928 | 0.936 |
|  | (0.0543) | (0.0445) | (0.0532) | (0.0492) | (0.0443) | (0.0464) | (0.056) | (0.0605) | (0.0863) |
| treatment#post_EQ# |  |  |  |  |  |  |  |  |  |
| Female |  |  | 1.091* |  |  |  |  |  | 1.079* |
|  |  |  | (0.0493) |  |  |  |  |  | (0.0467) |
| Maori |  |  |  | 0.756** |  |  |  |  | 0.734** |
|  |  |  |  | (0.106) |  |  |  |  | (0.105) |
| Sole_Dependent_Children |  |  |  |  | 1.129 |  |  |  | 1.097 |
|  |  |  |  |  | (0.238) |  |  |  | (0.263) |
| Age(25-35) |  |  |  |  |  | 0.964 |  |  | 0.969 |
|  |  |  |  |  |  | (0.0821) |  |  | (0.0879) |
| Migrant |  |  |  |  |  |  | 0.902 |  | 0.886 |
|  |  |  |  |  |  |  | (0.0764) |  | (0.0811) |
| Employed_During_Earthquake |  |  |  |  |  |  |  | 1.135*** | 1.129** |
|  |  |  |  |  |  |  |  | (0.055) | (0.0578) |
| Control | N | Y | Y | Y | Y | Y | Y | Y | Y |
| **Observations** | **49,647** | **49,611** | **49,611** | **49,611** | **49,611** | **49,611** | **49,611** | **49,611** | **49,611** |
| *Note: All models are with years fixed effect. Cluster standard errors (at the Territorial Authority level) are in parentheses. *p < 0.1, **p < 0.05, ***p < 0.01* | | | | | | | | | |

**Table 26: ologit – Impact of Earthquake to Income adequacy (Model 3)**

| **Variables** | **Income Adequacy** | | | | | | | | |
| --- | --- | --- | --- | --- | --- | --- | --- | --- | --- |
|  | (1) | (2) | (3) | (4) | (5) | (6) | (7) | (8) | (9) |
| treatment | 1.121* | 1.003 | 1.043 | 1.009 | 1.032 | 0.997 | 0.985 | 1.176*** | 1.178** |
|  | (0.0706) | (0.0743) | (0.085) | (0.0774) | (0.0803) | (0.0697) | (0.0823) | (0.0711) | (0.0929) |
| treatment#post_EQ | 1.157** | 1.162*** | 1.155*** | 1.174*** | 1.139*** | 1.205*** | 1.179*** | 0.972 | 1.047 |
|  | (0.0754) | (0.0536) | (0.0607) | (0.0496) | (0.0502) | (0.0481) | (0.0649) | (0.0469) | (0.0502) |
| treatment#post_EQ# |  |  |  |  |  |  |  |  |  |
| Female |  |  | 1.007 |  |  |  |  |  | 0.978 |
|  |  |  | (0.0369) |  |  |  |  |  | (0.0368) |
| Maori |  |  |  | 0.921 |  |  |  |  | 0.886 |
|  |  |  |  | (0.081) |  |  |  |  | (0.0835) |
| Sole_Dependent_Children |  |  |  |  | 1.535*** |  |  |  | 1.572*** |
|  |  |  |  |  | (0.217) |  |  |  | (0.207) |
| Age(25-35) |  |  |  |  |  | 0.792*** |  |  | 0.777*** |
|  |  |  |  |  |  | (0.0613) |  |  | (0.0597) |
| Migrant |  |  |  |  |  |  | 0.924 |  | 0.961 |
|  |  |  |  |  |  |  | (0.0966) |  | (0.103) |
| Employed_During_Earthquake |  |  |  |  |  |  |  | 1.256*** | 1.208*** |
|  |  |  |  |  |  |  |  | (0.0568) | (0.0491) |
| Control | N | Y | Y | Y | Y | Y | Y | Y | Y |
| **Observations** | **48,606** | **48,579** | **48,579** | **48,579** | **48,579** | **48,579** | **48,579** | **48,579** | **48,582** |
| *Note: All models are with years fixed effect. Cluster standard errors (at the Territorial Authority level) are in parentheses. *p < 0.1, **p < 0.05, ***p < 0.01* | | | | | | | | | |

**Table 27: ologit – Impact of Earthquake to Safety (Model 3)**

| **Variables** | **Safety** | | | | | | | | |
| --- | --- | --- | --- | --- | --- | --- | --- | --- | --- |
|  | (1) | (2) | (3) | (4) | (5) | (6) | (7) | (8) | (9) |
| treatment | 0.971 | 0.933 | 0.98 | 0.929 | 0.952 | 0.986 | 0.94 | 0.868 | 0.961 |
|  | (0.104) | (0.117) | (0.118) | (0.116) | (0.118) | (0.121) | (0.116) | (0.132) | (0.141) |
| treatment#post_EQ | 1.014 | 1.057 | 1.021 | 1.07 | 1.028 | 0.996 | 1.06 | 1.077 | 1.006 |
|  | (0.0637) | (0.0741) | (0.0813) | (0.08) | (0.0705) | (0.0734) | (0.0702) | (0.12) | (0.12) |
| treatment#post_EQ# |  |  |  |  |  |  |  |  |  |
| Female |  |  | 1.067 |  |  |  |  |  | 1.034 |
|  |  |  | (0.0712) |  |  |  |  |  | (0.0701) |
| Maori |  |  |  | 0.808 |  |  |  |  | 0.756** |
|  |  |  |  | (0.107) |  |  |  |  | (0.102) |
| Sole_Dependent_Children |  |  |  |  | 1.638*** |  |  |  | 1.643*** |
|  |  |  |  |  | (0.126) |  |  |  | (0.156) |
| Age(25-35) |  |  |  |  |  | 1.468*** |  |  | 1.450*** |
|  |  |  |  |  |  | (0.108) |  |  | (0.11) |
| Migrant |  |  |  |  |  |  | 1.002 |  | 1.006 |
|  |  |  |  |  |  |  | (0.0748) |  | (0.0729) |
| Employed_During_Earthquake |  |  |  |  |  |  |  | 0.982 | 0.962 |
|  |  |  |  |  |  |  |  | (0.0648) | (0.0626) |
| Control | N | Y | Y | Y | Y | Y | Y | Y | Y |
| **Observations** | **46,086** | **46,065** | **46,062** | **46,065** | **46,065** | **46,065** | **46,062** | **46,065** | **46,062** |
| *Note: All models are with years fixed effect. Cluster standard errors (at the Territorial Authority level) are in parentheses. *p < 0.1, **p < 0.05, ***p < 0.01* | | | | | | | | | |

**Table 28: gologit – Impact of Earthquake to Life Satisfaction (Model 1)**

| **Variables** | **>1 vs <=1** | **>2 vs <=2** | **>3 vs <=3** | **>4 vs <=4** | **>1 vs <=1** | **>2 vs <=2** | **>3 vs <=3** | **>4 vs <=4** | **>1 vs <=1** | **>2 vs <=2** | **>3 vs <=3** | **>4 vs <=4** |
| --- | --- | --- | --- | --- | --- | --- | --- | --- | --- | --- | --- | --- |
|  | (1) | (2) | (3) | (4) | (5) | (6) | (7) | (8) | (9) | (10) | (11) | (12) |
| treatment | 2.318*** | 1.230*** | 0.961 | 0.906** | 2.136*** | 1.187*** | 0.900*** | 0.911** | 1.298 | 0.716*** | 0.882* | 0.895* |
|  | (0.243) | (0.0626) | (0.0329) | (0.0353) | (0.226) | (0.0542) | (0.0279) | (0.0332) | (0.383) | (0.0723) | (0.0654) | (0.0555) |
| treatment#post_EQ | 0.281*** | 0.560*** | 0.810*** | 0.975 | 0.310*** | 0.599*** | 0.845*** | 0.972 | 0.436** | 1.063 | 0.761*** | 0.95 |
|  | (0.0287) | (0.0315) | (0.0311) | (0.0384) | (0.0322) | (0.0304) | (0.0299) | (0.0352) | (0.163) | (0.143) | (0.0636) | (0.0675) |
| treatment#post_EQ# |  |  |  |  |  |  |  |  |  |  |  |  |
| Female |  |  |  |  |  |  |  |  | 1.355 | 0.561*** | 1.025 | 0.931* |
|  |  |  |  |  |  |  |  |  | (0.405) | (0.0509) | (0.0547) | (0.0346) |
| Maori |  |  |  |  |  |  |  |  | 0.788 | 0.0345*** | 0.553*** | 0.523*** |
|  |  |  |  |  |  |  |  |  |  | (0.0356) | (0.0563) | (0.0416) |
| Migrant |  |  |  |  |  |  |  |  | 0.187 | 1.783*** | 1.243*** | 0.886* |
|  |  |  |  |  |  |  |  |  | (0.201) | (0.167) | (0.0781) | (0.058) |
| Sole Dependent Children |  |  |  |  |  |  |  |  | 0.318 | 111.8*** | 0.0143*** | 0.663*** |
|  |  |  |  |  |  |  |  |  | (0.351) | (126.1) | (0.0145) | (0.0662) |
| Age(25-35) |  |  |  |  |  |  |  |  | 0.0832*** | 0.942 | 1.312*** | 1.148** |
|  |  |  |  |  |  |  |  |  | (0.076) | (0.0913) | (0.115) | (0.0773) |
| Employed During Earthquake |  |  |  |  |  |  |  |  | 1.092 | 0.706*** | 1.136* | 1.192*** |
|  |  |  |  |  |  |  |  |  | (0.373) | (0.0954) | (0.0762) | (0.0636) |
| Constant | 70.09*** | 12.23*** | 5.361*** | 0.459*** | 112.7*** | 14.79*** | 3.755*** | 0.300*** | 104.1*** | 15.26*** | 3.513*** | 0.308*** |
|  | (11.31) | (0.793) | (0.27) | (0.0249) | (27.54) | (1.794) | (0.377) | (0.0252) | (31.91) | (2.049) | (0.387) | (0.0319) |
| Control variables | N | N | N | N | Y | Y | Y | Y | Y | Y | Y | Y |
| **Observations** | **45,348** | **45,348** | **45,348** | **45,345** | **45,312** | **45,312** | **45,312** | **45,312** | **45,312** | **45,312** | **45,312** | **45,312** |
| *Note: Generalized ordered logistic (GOL) regression model compares all the categories greater than the current category to those less than or equal to the current category (i.e. > vs. ≤ categories comparison). Then we have each column from GOL compare from ( >1 vs <=1) to (>4 vs <=4).* | | | | | | | | | | | | |

**Table 29: gologit – Impact of Earthquake to General Health (Model 1)**

| **Variables** | **>1 vs <=1** | **>2 vs <=2** | **>3 vs <=3** | **>4 vs <=4** | **>1 vs <=1** | **>2 vs <=2** | **>3 vs <=3** | **>4 vs <=4** | **>1 vs <=1** | **>2 vs <=2** | **>3 vs <=3** | **>4 vs <=4** |
| --- | --- | --- | --- | --- | --- | --- | --- | --- | --- | --- | --- | --- |
|  | (1) | (2) | (3) | (4) | (5) | (6) | (7) | (8) | (9) | (10) | (11) | (12) |
| treatment | 1.212*** | 1.298*** | 1.162*** | 1.026 | 1.153** | 1.254*** | 1.109*** | 0.978 | 2.550*** | 1.476*** | 1.221*** | 1.344*** |
|  | (0.0804) | (0.0574) | (0.0444) | (0.0418) | (0.0763) | (0.0486) | (0.0394) | (0.0386) | (0.369) | (0.121) | (0.0728) | (0.11) |
| treatment#post_EQ | 0.752*** | 0.800*** | 0.836*** | 0.976 | 0.758*** | 0.799*** | 0.838*** | 0.991 | 0.457*** | 0.810** | 0.708*** | 0.570*** |
|  | (0.0499) | (0.0438) | (0.0476) | (0.0548) | (0.0457) | (0.0354) | (0.0416) | (0.0476) | (0.0751) | (0.0822) | (0.0632) | (0.0574) |
| treatment#post_EQ# |  |  |  |  |  |  |  |  |  |  |  |  |
| Female |  |  |  |  |  |  |  |  | 1.309** | 0.860*** | 1.100** | 1.035 |
|  |  |  |  |  |  |  |  |  | (0.154) | (0.04) | (0.0449) | (0.0572) |
| Maori |  |  |  |  |  |  |  |  | 6.96e-08*** | 0.151*** | 0.282*** | 0.387*** |
|  |  |  |  |  |  |  |  |  | (0.0000000715) | (0.0116) | (0.0179) | (0.0319) |
| Migrant |  |  |  |  |  |  |  |  | 0.257*** | 1.099 | 0.683*** | 0.865* |
|  |  |  |  |  |  |  |  |  | (0.0425) | (0.0846) | (0.0376) | (0.0714) |
| Sole Dependent Children |  |  |  |  |  |  |  |  | 1.95e-06*** | 2.238*** | 3.049*** | 2.831*** |
|  |  |  |  |  |  |  |  |  | (0.00000202) | (0.235) | (0.248) | (0.325) |
| Age(25-35) |  |  |  |  |  |  |  |  | 2.85e-06*** | 1.041 | 0.944 | 1.174** |
|  |  |  |  |  |  |  |  |  | (0.00000301) | (0.0976) | (0.059) | (0.0953) |
| Employed During Earthquake |  |  |  |  |  |  |  |  | 2.775*** | 1.159* | 1.353*** | 2.125*** |
|  |  |  |  |  |  |  |  |  | (0.395) | (0.0887) | (0.0737) | (0.136) |
| Constant | 26.56*** | 5.464*** | 1.267*** | 0.268*** | 48.69*** | 9.509*** | 1.759*** | 0.401*** | 39.73*** | 8.508*** | 1.647*** | 0.348*** |
|  | (2.693) | (0.391) | (0.0816) | (0.0209) | (13.08) | (1.43) | (0.214) | (0.061) | (11.65) | (1.335) | (0.212) | (0.0563) |
| Control variables | N | N | N | N | Y | Y | Y | Y | Y | Y | Y | Y |
| **Observations** | **45,381** | **45,384** | **45,384** | **45,384** | **45,348** | **45,348** | **45,348** | **45,348** | **45,348** | **45,348** | **45,348** | **45,348** |
| *Note: Generalized ordered logistic (GOL) regression model compares all the categories greater than the current category to those less than or equal to the current category (i.e. > vs. ≤ categories comparison). Then we have each column from GOL compare from ( >1 vs <=1) to (>4 vs <=4).* | | | | | | | | | | | | |

**Table 30: gologit – Impact of Earthquake to Income Adequacy (Model 1)**

| **Variables** | **>0 vs <=0** | **>1 vs <=1** | **>2 vs <=2** | **>0 vs <=0** | **>1 vs <=1** | **>2 vs <=2** | **>0 vs <=0** | **>1 vs <=1** | **>2 vs <=2** |
| --- | --- | --- | --- | --- | --- | --- | --- | --- | --- |
|  | (1) | (2) | (3) | (4) | (5) | (6) | (7) | (8) | (9) |
| treatment | 1.407*** | 1.503*** | 1.159* | 1.244*** | 1.386*** | 1.089 | 1.614*** | 1.639*** | 1.146 |
|  | (0.0756) | (0.0756) | (0.0907) | (0.0462) | (0.064) | (0.0814) | (0.102) | (0.0895) | (0.104) |
| treatment#post_EQ | 0.823** | 0.843*** | 1.152*** | 0.830*** | 0.841*** | 1.174*** | 0.924 | 0.662*** | 1.02 |
|  | (0.0662) | (0.0555) | (0.0563) | (0.0407) | (0.0316) | (0.0431) | (0.0802) | (0.0321) | (0.0815) |
| treatment#post_EQ# |  |  |  |  |  |  |  |  |  |
| Female |  |  |  |  |  |  | 0.797*** | 1.028 | 0.751*** |
|  |  |  |  |  |  |  | (0.0361) | (0.0361) | (0.0361) |
| Maori |  |  |  |  |  |  | 1.699*** | 0.505*** | 0.402*** |
|  |  |  |  |  |  |  | (0.145) | (0.0496) | (0.0502) |
| Migrant |  |  |  |  |  |  | 0.587*** | 1.087 | 1.074 |
|  |  |  |  |  |  |  | (0.0472) | (0.104) | (0.134) |
| Sole Dependent Children |  |  |  |  |  |  | 0.979 | 1.03 | 0.885 |
|  |  |  |  |  |  |  | (0.0996) | (0.0971) | (0.158) |
| Age(25-35) |  |  |  |  |  |  | 0.558*** | 1.138** | 1.156 |
|  |  |  |  |  |  |  | (0.0444) | -0.0662 | (0.139) |
| Employed During Earthquake |  |  |  |  |  |  | 1.246*** | 1.324*** | 1.505*** |
|  |  |  |  |  |  |  | (0.0903) | (0.0527) | (0.122) |
| Constant | 44,427 | 44,427 | 44,427 | 44,402 | 44,402 | 44,402 | 44,402 | 44,402 | 44,402 |
|  | (2.693) | (0.391) | (0.0816) | (0.0209) | (13.08) | (1.43) | (0.214) | (0.061) | (11.65) |
| Control variables | N | N | N | Y | Y | Y | Y | Y | Y |
| **Observations** | **45,381** | **45,384** | **45,384** | **45,384** | **45,348** | **45,348** | **45,348** | **45,348** | **45,348** |
| *Note: Generalized ordered logistic (GOL) regression model compares all the categories greater than the current category to those less than or equal to the current category (i.e. > vs. ≤ categories comparison). Then we have each column from GOL compare from ( >1 vs <=1) to (>4 vs <=4).* | | | | | | | | | |

**Table 31: gologit – Impact of Earthquake to Safety (Model 1)**

| **Variables** | **>1 vs <=1** | **>2 vs <=2** | **>3 vs <=3** | **>4 vs <=4** | **>1 vs <=1** | **>2 vs <=2** | **>3 vs <=3** | **>4 vs <=4** | **>1 vs <=1** | **>2 vs <=2** | **>3 vs <=3** | **>4 vs <=4** |
| --- | --- | --- | --- | --- | --- | --- | --- | --- | --- | --- | --- | --- |
|  | (1) | (2) | (3) | (4) | (5) | (6) | (7) | (8) | (9) | (10) | (11) | (12) |
| treatment | 1.448*** | 1.079 | 0.906 | 0.834** | 1.503*** | 1.082 | 0.876* | 0.811** | 5.510*** | 0.991 | 0.801*** | 0.446*** |
|  | (0.0863) | (0.0684) | (0.0559) | (0.0715) | (0.108) | (0.0772) | (0.0631) | (0.0711) | (0.657) | (0.0985) | (0.0663) | (0.0457) |
| treatment#post_EQ | 0.679*** | 0.879*** | 0.957 | 1.104 | 0.712*** | 0.920* | 1.024 | 1.143* | 0.293*** | 1.117 | 1.379*** | 2.159*** |
|  | (0.0489) | (0.0437) | (0.0426) | (0.086) | (0.0397) | (0.0464) | (0.0473) | (0.0825) | (0.045) | (0.109) | (0.11) | (0.21) |
| treatment#post_EQ# |  |  |  |  |  |  |  |  |  |  |  |  |
| Female |  |  |  |  |  |  |  |  | 2.304*** | 1.497*** | 1.141** | 1.029 |
|  |  |  |  |  |  |  |  |  | (0.303) | (0.118) | (0.0683) | (0.0685) |
| Maori |  |  |  |  |  |  |  |  | 2.345*** | 1.055 | 1.364*** | 0.723*** |
|  |  |  |  |  |  |  |  |  | (0.296) | (0.101) | (0.111) | (0.0864) |
| Migrant |  |  |  |  |  |  |  |  | 1.691*** | 1.463*** | 0.891** | 1.976*** |
|  |  |  |  |  |  |  |  |  | (0.134) | (0.0728) | (0.0447) | (0.163) |
| Sole Dependent Children |  |  |  |  |  |  |  |  | 3.875*** | 1.643*** | 1.792*** | 0.299*** |
|  |  |  |  |  |  |  |  |  | (0.432) | (0.136) | (0.143) | (0.0449) |
| Age(25-35) |  |  |  |  |  |  |  |  | 1.328** | 0.758*** | 0.993 | 1.169** |
|  |  |  |  |  |  |  |  |  | (0.175) | (0.0536) | (0.0556) | (0.0845) |
| Employed During Earthquake |  |  |  |  |  |  |  |  | 0.884 | 0.480*** | 0.593*** | 0.390*** |
|  |  |  |  |  |  |  |  |  | (0.0899) | (0.0328) | (0.0309) | (0.0272) |
| Constant | 7.711*** | 3.912*** | 1.186** | 0.157*** | 22.62*** | 11.82*** | 3.160*** | 0.376*** | 21.31*** | 10.57*** | 3.293*** | 0.405*** |
|  | (0.673) | (0.282) | (0.0986) | (0.0165) | (3.944) | (1.433) | (0.35) | (0.0457) | (3.779) | (1.46) | (0.381) | (0.0543) |
| Control variables | N | N | N | N | Y | Y | Y | Y | Y | Y | Y | Y |
| **Observations** | **42,054** | **42,051** | **42,054** | **42,054** | **42,033** | **42,033** | **42,036** | **42,033** | **42,036** | **42,033** | **42,036** | **42,033** |
| *Note: Generalized ordered logistic (GOL) regression model compares all the categories greater than the current category to those less than or equal to the current category (i.e. > vs. ≤ categories comparison). Then we have each column from GOL compare from ( >1 vs <=1) to (>4 vs <=4).* | | | | | | | | | | | | |

**Table 32: gologit – Impact of Earthquake to Life Satisfaction (Model 2)**

| **Variables** | **>1 vs <=1** | **>2 vs <=2** | **>3 vs <=3** | **>4 vs <=4** | **>1 vs <=1** | **>2 vs <=2** | **>3 vs <=3** | **>4 vs <=4** | **>1 vs <=1** | **>2 vs <=2** | **>3 vs <=3** | **>4 vs <=4** |
| --- | --- | --- | --- | --- | --- | --- | --- | --- | --- | --- | --- | --- |
|  | (1) | (2) | (3) | (4) | (5) | (6) | (7) | (8) | (9) | (10) | (11) | (12) |
| treatment | 2.636*** | 1.181** | 1.009 | 0.94 | 2.432*** | 1.131 | 0.937 | 0.927* | 1.311 | 0.872 | 1.033 | 0.904 |
|  | (0.526) | (0.087) | (0.0683) | (0.0562) | (0.504) | (0.0871) | (0.0532) | (0.0387) | (0.358) | (0.228) | (0.221) | (0.057) |
| treatment#post_EQ | 0.262*** | 0.605*** | 0.787*** | 0.962 | 0.280*** | 0.638*** | 0.809*** | 0.966 | 0.475* | 0.988 | 0.744*** | 1.051 |
|  | (0.0459) | (0.061) | (0.0468) | (0.0448) | (0.0528) | (0.0542) | (0.0526) | (0.0362) | (0.191) | (0.185) | (0.0812) | (0.15) |
| treatment#post_EQ# |  |  |  |  |  |  |  |  |  |  |  |  |
| Female |  |  |  |  |  |  |  |  | 1.234 | 0.653* | 0.968 | 1.019 |
|  |  |  |  |  |  |  |  |  | (0.419) | (0.152) | (0.0787) | (0.134) |
| Maori |  |  |  |  |  |  |  |  | 2.86e-07*** | 0.194 | 0.503*** | 0.561*** |
|  |  |  |  |  |  |  |  |  | (0.000000362) | (0.23) | (0.0691) | (0.105) |
| Migrant |  |  |  |  |  |  |  |  | 2.72e-06*** | 1.296 | 1.052 | 0.756 |
|  |  |  |  |  |  |  |  |  | (0.00000263) | (0.48) | (0.192) | (0.142) |
| Sole Dependent Children |  |  |  |  |  |  |  |  | 1.36e-06*** | 1.475 | 0.620* | 0.945 |
|  |  |  |  |  |  |  |  |  | (0.0000013) | (0.51) | (0.175) | (0.204) |
| Age(25-35) |  |  |  |  |  |  |  |  | 8.83e-06*** | 1.139 | 1.271** | 1.107 |
|  |  |  |  |  |  |  |  |  | (0.00000908) | (0.224) | (0.119) | (0.0836) |
| Employed During Earthquake |  |  |  |  |  |  |  |  | 0.938 | 0.801 | 1.186** | 1.005 |
|  |  |  |  |  |  |  |  |  | (0.41) | (0.163) | (0.103) | (0.202) |
| Constant | 70.03*** | 12.24*** | 5.369*** | 0.461*** | 113.0*** | 14.89*** | 3.782*** | 0.301*** | 103.3*** | 14.83*** | 3.463*** | 0.306*** |
|  | (11.28) | (0.791) | (0.27) | (0.0251) | (27.56) | (1.804) | (0.378) | (0.0252) | (31.68) | (2.002) | (0.38) | (0.0316) |
| Control variables | N | N | N | N | Y | Y | Y | Y | Y | Y | Y | Y |
| **Observations** | **45,600** | **45,600** | **45,600** | **45,600** | **45,564** | **45,564** | **45,564** | **45,564** | **45,564** | **45,564** | **45,564** | **45,564** |
| *Note: Generalized ordered logistic (GOL) regression model compares all the categories greater than the current category to those less than or equal to the current category (i.e. > vs. ≤ categories comparison). Then we have each column from GOL compare from ( >1 vs <=1) to (>4 vs <=4).* | | | | | | | | | | | | |

**Table 33: gologit – Impact of Earthquake to General Health (Model 2)**

| **Variables** | **>1 vs <=1** | **>2 vs <=2** | **>3 vs <=3** | **>4 vs <=4** | **>1 vs <=1** | **>2 vs <=2** | **>3 vs <=3** | **>4 vs <=4** | **>1 vs <=1** | **>2 vs <=2** | **>3 vs <=3** | **>4 vs <=4** |
| --- | --- | --- | --- | --- | --- | --- | --- | --- | --- | --- | --- | --- |
|  | (1) | (2) | (3) | (4) | (5) | (6) | (7) | (8) | (9) | (10) | (11) | (12) |
| treatment | 1.154 | 1.200* | 1.119* | 1.047 | 1.089 | 1.165 | 1.075 | 1.005 | 2.455*** | 1.404*** | 1.018 | 1.277* |
|  | (0.104) | (0.13) | (0.068) | (0.0485) | (0.0998) | (0.115) | (0.0564) | (0.0507) | (0.376) | (0.15) | (0.249) | (0.176) |
| treatment#post_EQ | 0.782*** | 0.874 | 0.892 | 0.982 | 0.787*** | 0.869 | 0.89 | 0.992 | 0.506*** | 0.88 | 0.864 | 0.585*** |
|  | (0.0612) | (0.112) | (0.0826) | (0.063) | (0.0558) | (0.106) | (0.0758) | (0.0601) | (0.101) | (0.13) | (0.236) | (0.071) |
| treatment#post_EQ# |  |  |  |  |  |  |  |  |  |  |  |  |
| Female |  |  |  |  |  |  |  |  | 1.301** | 0.946 | 1.171** | 1.139 |
|  |  |  |  |  |  |  |  |  | (0.168) | (0.11) | (0.0902) | (0.132) |
| Maori |  |  |  |  |  |  |  |  | 9.81e-08*** | 0.501 | 0.384*** | 0.447*** |
|  |  |  |  |  |  |  |  |  | (0.000000089) | (0.416) | (0.122) | (0.0757) |
| Migrant |  |  |  |  |  |  |  |  | 0.217*** | 0.88 | 0.562** | 0.741 |
|  |  |  |  |  |  |  |  |  | (0.0667) | (0.271) | (0.142) | (0.163) |
| Sole Dependent Children |  |  |  |  |  |  |  |  | 0.616 | 1.825** | 1.789 | 1.795 |
|  |  |  |  |  |  |  |  |  | (0.588) | (0.479) | (1.015) | (0.776) |
| Age(25-35) |  |  |  |  |  |  |  |  | 5.94e-07*** | 1.018 | 0.961 | 1.170* |
|  |  |  |  |  |  |  |  |  | (0.000000523) | (0.138) | (0.0715) | (0.0965) |
| Employed During Earthquake |  |  |  |  |  |  |  |  | 2.396*** | 1.092 | 1.143 | 2.027*** |
|  |  |  |  |  |  |  |  |  | (0.502) | (0.112) | (0.268) | (0.2) |
| Constant | 26.46*** | 5.447*** | 1.263*** | 0.267*** | 48.71*** | 9.464*** | 1.744*** | 0.397*** | 39.63*** | 8.440*** | 1.635*** | 0.345*** |
|  | (2.668) | (0.388) | (0.0813) | (0.0208) | (13.01) | (1.418) | (0.212) | (0.0604) | (11.58) | (1.323) | (0.21) | (0.0558) |
| Control variables | N | N | N | N | Y | Y | Y | Y | Y | Y | Y | Y |
| **Observations** | **45,636** | **45,636** | **45,636** | **45,636** | **45,600** | **45,600** | **45,600** | **45,600** | **45,600** | **45,600** | **45,600** | **45,600** |
| *Note: Generalized ordered logistic (GOL) regression model compares all the categories greater than the current category to those less than or equal to the current category (i.e. > vs. ≤ categories comparison). Then we have each column from GOL compare from ( >1 vs <=1) to (>4 vs <=4).* | | | | | | | | | | | | |

**Table 34: gologit – Impact of Earthquake to Income Adequacy (Model 2)**

| **Variables** | **>0 vs <=0** | **>1 vs <=1** | **>2 vs <=2** | **>0 vs <=0** | **>1 vs <=1** | **>2 vs <=2** | **>0 vs <=0** | **>1 vs <=1** | **>2 vs <=2** |
| --- | --- | --- | --- | --- | --- | --- | --- | --- | --- |
|  | (1) | (2) | (3) | (4) | (5) | (6) | (7) | (8) | (9) |
| treatment | 1.297** | 1.411*** | 1.143 | 1.126 | 1.279** | 1.069 | 1.674*** | 1.531*** | 1.128 |
|  | (0.138) | (0.133) | (0.101) | (0.134) | (0.141) | (0.0936) | (0.129) | (0.187) | (0.216) |
| treatment#post_EQ | 0.934 | 0.927 | 1.139** | 0.931 | 0.92 | 1.136** | 0.876 | 0.734** | 0.956 |
|  | (0.146) | (0.116) | (0.0632) | (0.123) | (0.103) | (0.065) | (0.0992) | (0.0915) | (0.165) |
| treatment#post_EQ# |  |  |  |  |  |  |  |  |  |
| Female |  |  |  |  |  |  | 0.934 | 1.115 | 0.893 |
|  |  |  |  |  |  |  | (0.178) | (0.178) | (0.178) |
| Maori |  |  |  |  |  |  | 2.079*** | 0.597** | 0.399*** |
|  |  |  |  |  |  |  | (0.446) | (0.131) | (0.0503) |
| Migrant |  |  |  |  |  |  | 0.554*** | 0.98 | 0.922 |
|  |  |  |  |  |  |  | (0.0582) | (0.145) | (0.241) |
| Sole Dependent Children |  |  |  |  |  |  | 0.801 | 1.007 | 1.335 |
|  |  |  |  |  |  |  | (0.18) | (0.0984) | (0.686) |
| Age(25-35) |  |  |  |  |  |  | 0.619*** | 1.045 | 1.224 |
|  |  |  |  |  |  |  | (0.0948) | (0.114) | (0.174) |
| Employed During Earthquake |  |  |  |  |  |  | 1.324*** | 1.279*** | 1.433** |
|  |  |  |  |  |  |  | (0.131) | (0.0721) | (0.23) |
| Constant | 5.008*** | 1 | 0.153*** | 4.536*** | 0.877* | 0.135*** | 4.242*** | 0.842* | 0.121*** |
|  | (0.355) | (0.0614) | (0.0171) | (0.446) | (0.0662) | (0.0192) | (0.441) | (0.0784) | (0.0188) |
| Control variables | N | N | N | Y | Y | Y | Y | Y | Y |
| **Observations** | **44,676** | **44,676** | **44,676** | **44,649** | **44,649** | **44,649** | **44,649** | **44,652** | **44,649** |
| *Note: Generalized ordered logistic (GOL) regression model compares all the categories greater than the current category to those less than or equal to the current category (i.e. > vs. ≤ categories comparison). Then we have each column from GOL compare from ( >1 vs <=1) to (>4 vs <=4).* | | | | | | | | | |

**Table 35: gologit – Impact of Earthquake to Safety (Model 2)**

| **Variables** | **>1 vs <=1** | **>2 vs <=2** | **>3 vs <=3** | **>4 vs <=4** | **>1 vs <=1** | **>2 vs <=2** | **>3 vs <=3** | **>4 vs <=4** | **>1 vs <=1** | **>2 vs <=2** | **>3 vs <=3** | **>4 vs <=4** |
| --- | --- | --- | --- | --- | --- | --- | --- | --- | --- | --- | --- | --- |
|  | (1) | (2) | (3) | (4) | (5) | (6) | (7) | (8) | (9) | (10) | (11) | (12) |
| treatment | 1.579*** | 1.134 | 0.91 | 0.867 | 1.658*** | 1.168 | 0.901 | 0.853 | 7.289*** | 1.119 | 0.865 | 0.516*** |
|  | (0.197) | (0.0971) | (0.0575) | (0.0886) | (0.241) | (0.135) | (0.0695) | (0.0911) | (2.369) | (0.186) | (0.0968) | (0.103) |
| treatment#post_EQ | 0.660*** | 0.868** | 1.002 | 1.155 | 0.689*** | 0.883* | 1.044 | 1.173** | 0.206*** | 0.999 | 1.349*** | 1.942*** |
|  | (0.0597) | (0.0493) | (0.0669) | (0.106) | (0.0556) | (0.0637) | (0.0546) | (0.0922) | (0.0808) | (0.172) | (0.124) | (0.322) |
| treatment#post_EQ# |  |  |  |  |  |  |  |  |  |  |  |  |
| Female |  |  |  |  |  |  |  |  | 3.163*** | 1.534*** | 1.121* | 1.387 |
|  |  |  |  |  |  |  |  |  | (1.127) | (0.134) | (0.0749) | (0.485) |
| Maori |  |  |  |  |  |  |  |  | 1.301 | 1.023 | 1.194 | 1.244 |
|  |  |  |  |  |  |  |  |  | (0.708) | (0.168) | (0.146) | (0.772) |
| Migrant |  |  |  |  |  |  |  |  | 1.515** | 1.206 | 0.74 | 1.611* |
|  |  |  |  |  |  |  |  |  | (0.276) | (0.29) | (0.17) | (0.417) |
| Sole Dependent Children |  |  |  |  |  |  |  |  | 2.969*** | 1.526*** | 1.784*** | 0.503 |
|  |  |  |  |  |  |  |  |  | (0.718) | (0.224) | (0.201) | (0.247) |
| Age(25-35) |  |  |  |  |  |  |  |  | 1.686 | 0.956 | 1.181 | 1.065 |
|  |  |  |  |  |  |  |  |  | (0.576) | (0.297) | (0.253) | (0.126) |
| Employed During Earthquake |  |  |  |  |  |  |  |  | 0.971 | 0.526*** | 0.656*** | 0.421*** |
|  |  |  |  |  |  |  |  |  | (0.205) | (0.0706) | (0.0841) | (0.0561) |
| Constant | 7.726*** | 3.919*** | 1.189** | 0.156*** | 22.54*** | 11.75*** | 3.139*** | 0.374*** | 21.30*** | 10.51*** | 3.267*** | 0.404*** |
|  | (0.674) | (0.282) | (0.0989) | (0.0164) | (3.919) | (1.419) | (0.345) | (0.0453) | (3.767) | (1.447) | (0.377) | (0.0542) |
| Control variables | N | N | N | N | Y | Y | Y | Y | Y | Y | Y | Y |
| **Observations** | **42,294** | **42,294** | **42,294** | **42,294** | **42,276** | **42,273** | **42,276** | **42,273** | **42,276** | **42,276** | **42,276** | **42,276** |
| *Note: Generalized ordered logistic (GOL) regression model compares all the categories greater than the current category to those less than or equal to the current category (i.e. > vs. ≤ categories comparison). Then we have each column from GOL compare from ( >1 vs <=1) to (>4 vs <=4).* | | | | | | | | | | | | |

**Table 36: gologit – Impact of Earthquake to Life Satisfaction (Model 3)**

| **Variables** | **>1 vs <=1** | **>2 vs <=2** | **>3 vs <=3** | **>4 vs <=4** | **>1 vs <=1** | **>2 vs <=2** | **>3 vs <=3** | **>4 vs <=4** | **>1 vs <=1** | **>2 vs <=2** | **>3 vs <=3** | **>4 vs <=4** |
| --- | --- | --- | --- | --- | --- | --- | --- | --- | --- | --- | --- | --- |
|  | (1) | (2) | (3) | (4) | (5) | (6) | (7) | (8) | (9) | (10) | (11) | (12) |
| treatment | 1.810*** | 1.05 | 0.925 | 1.021 | 1.833*** | 1.059 | 0.898* | 1.01 | 1.462 | 0.845 | 0.916 | 0.948 |
|  | (0.376) | (0.0666) | (0.0711) | (0.089) | (0.363) | (0.0535) | (0.051) | (0.068) | (0.464) | (0.121) | (0.125) | (0.101) |
| treatment#post_EQ | 0.394*** | 0.727*** | 0.907* | 0.906 | 0.388*** | 0.708*** | 0.887*** | 0.912 | 0.375*** | 0.862 | 0.867 | 1.005 |
|  | (0.0804) | (0.0398) | (0.0479) | (0.0717) | (0.0794) | (0.0341) | (0.041) | (0.0625) | (0.122) | (0.113) | (0.0796) | (0.0892) |
| treatment#post_EQ# |  |  |  |  |  |  |  |  |  |  |  |  |
| Female |  |  |  |  |  |  |  |  | 0.451*** | 0.703*** | 0.924 | 1.006 |
|  |  |  |  |  |  |  |  |  | (0.0857) | (0.0888) | (0.0892) | (0.0752) |
| Maori |  |  |  |  |  |  |  |  | 1.693 | 0.593*** | 0.785** | 0.953 |
|  |  |  |  |  |  |  |  |  | (0.558) | (0.0823) | (0.08) | (0.111) |
| Migrant |  |  |  |  |  |  |  |  | 1.287 | 1.252* | 1.171* | 0.921 |
|  |  |  |  |  |  |  |  |  | (0.317) | (0.157) | (0.104) | (0.163) |
| Sole Dependent Children |  |  |  |  |  |  |  |  | 1.407 | 1.636** | 1.147 | 1.006 |
|  |  |  |  |  |  |  |  |  | (0.398) | (0.318) | (0.192) | (0.168) |
| Age(25-35) |  |  |  |  |  |  |  |  | 1.534 | 1.121 | 1.119 | 1.015 |
|  |  |  |  |  |  |  |  |  | (0.452) | (0.117) | (0.109) | (0.077) |
| Employed During Earthquake |  |  |  |  |  |  |  |  | 1.421 | 0.94 | 1.021 | 0.908 |
|  |  |  |  |  |  |  |  |  | (0.374) | (0.148) | (0.0953) | (0.0655) |
| Constant | 74.07*** | 12.14*** | 5.341*** | 0.452*** | 113.2*** | 14.92*** | 3.914*** | 0.288*** | 105.0*** | 15.26*** | 3.605*** | 0.293*** |
|  | (12.56) | (0.751) | (0.254) | (0.0237) | (26.61) | (1.663) | (0.393) | (0.0226) | (30.72) | (1.915) | (0.416) | (0.029) |
| Control variables | N | N | N | N | Y | Y | Y | Y | Y | Y | Y | Y |
| **Observations** | **49,608** | **49,608** | **49,608** | **49,608** | **49,572** | **49,572** | **49,572** | **49,572** | **49,569** | **49,572** | **49,572** | **49,569** |
| *Note: Generalized ordered logistic (GOL) regression model compares all the categories greater than the current category to those less than or equal to the current category (i.e. > vs. ≤ categories comparison). Then we have each column from GOL compare from ( >1 vs <=1) to (>4 vs <=4).* | | | | | | | | | | | | |

**Table 37: gologit – Impact of Earthquake to General Health (Model 3)**

| **Variables** | **>1 vs <=1** | **>2 vs <=2** | **>3 vs <=3** | **>4 vs <=4** | **>1 vs <=1** | **>2 vs <=2** | **>3 vs <=3** | **>4 vs <=4** | **>1 vs <=1** | **>2 vs <=2** | **>3 vs <=3** | **>4 vs <=4** |
| --- | --- | --- | --- | --- | --- | --- | --- | --- | --- | --- | --- | --- |
|  | (1) | (2) | (3) | (4) | (5) | (6) | (7) | (8) | (9) | (10) | (11) | (12) |
| treatment | 0.951 | 0.995 | 0.961 | 0.958 | 0.929 | 0.965 | 0.922** | 0.926 | 1.395 | 1.062 | 0.828*** | 1.12 |
|  | (0.0779) | (0.0529) | (0.0373) | (0.052) | (0.0601) | (0.042) | (0.0327) | (0.0494) | (0.421) | (0.108) | (0.0581) | (0.104) |
| treatment#post_EQ | 0.995 | 1.011 | 1.024 | 1.109 | 0.941 | 0.978 | 1.003 | 1.094 | 0.805 | 0.961 | 1.079 | 0.752** |
|  | (0.0684) | (0.0608) | (0.062) | (0.0751) | (0.0513) | (0.0525) | (0.0545) | (0.0708) | (0.248) | (0.101) | (0.143) | (0.0872) |
| treatment#post_EQ# |  |  |  |  |  |  |  |  |  |  |  |  |
| Female |  |  |  |  |  |  |  |  | 1.22 | 0.879 | 1.007 | 1.365*** |
|  |  |  |  |  |  |  |  |  | (0.195) | (0.0763) | (0.0529) | (0.0777) |
| Maori |  |  |  |  |  |  |  |  | 1.164 | 1.224 | 0.668*** | 0.582*** |
|  |  |  |  |  |  |  |  |  | (0.275) | (0.264) | (0.101) | (0.0686) |
| Migrant |  |  |  |  |  |  |  |  | 1.383 | 1.173* | 0.757*** | 0.952 |
|  |  |  |  |  |  |  |  |  | (0.379) | (0.0958) | (0.0768) | (0.14) |
| Sole Dependent Children |  |  |  |  |  |  |  |  | 0.771 | 0.922 | 0.991 | 1.603** |
|  |  |  |  |  |  |  |  |  | (0.194) | (0.229) | (0.329) | (0.333) |
| Age(25-35) |  |  |  |  |  |  |  |  | 1.308 | 1.022 | 1.015 | 0.929 |
|  |  |  |  |  |  |  |  |  | (0.298) | (0.173) | (0.126) | (0.0809) |
| Employed During Earthquake |  |  |  |  |  |  |  |  | 0.983 | 1.056 | 1.011 | 1.390*** |
|  |  |  |  |  |  |  |  |  | (0.198) | (0.0996) | (0.086) | (0.0916) |
| Constant | 25.84*** | 5.299*** | 1.264*** | 0.266*** | 46.78*** | 9.057*** | 1.732*** | 0.393*** | 37.72*** | 8.086*** | 1.630*** | 0.341*** |
|  | (2.507) | (0.363) | (0.0777) | (0.0197) | (11.55) | (1.317) | (0.199) | (0.0566) | (10.42) | (1.262) | (0.199) | (0.053) |
| Control variables | N | N | N | N | Y | Y | Y | Y | Y | Y | Y | Y |
| **Observations** | **49,647** | **49,647** | **49,647** | **49,647** | **49,611** | **49,611** | **49,611** | **49,611** | **49,611** | **49,611** | **49,611** | **49,611** |
| *Note: Generalized ordered logistic (GOL) regression model compares all the categories greater than the current category to those less than or equal to the current category (i.e. > vs. ≤ categories comparison). Then we have each column from GOL compare from ( >1 vs <=1) to (>4 vs <=4).* | | | | | | | | | | | | |

**Table 38: gologit – Impact of Earthquake to Income Adequacy (Model 3)**

| **Variables** | **>0 vs <=0** | **>1 vs <=1** | **>2 vs <=2** | **>0 vs <=0** | **>1 vs <=1** | **>2 vs <=2** | **>0 vs <=0** | **>1 vs <=1** | **>2 vs <=2** |
| --- | --- | --- | --- | --- | --- | --- | --- | --- | --- |
|  | (1) | (2) | (3) | (4) | (5) | (6) | (7) | (8) | (9) |
| treatment | 1.141** | 1.127** | 1.09 | 0.991 | 1.011 | 1.001 | 1.102 | 1.188** | 1.254** |
|  | (0.0743) | (0.0671) | (0.101) | (0.0823) | (0.074) | (0.0947) | (0.0942) | (0.103) | (0.131) |
| treatment#post_EQ | 1.096 | 1.186** | 1.151** | 1.07 | 1.192** | 1.155*** | 1.061 | 1.114 | 0.898 |
|  | (0.108) | (0.0994) | (0.0689) | (0.0839) | (0.082) | (0.0607) | (0.125) | (0.0893) | (0.0831) |
| treatment#post_EQ# |  |  |  |  |  |  |  |  |  |
| Female |  |  |  |  |  |  | 0.93 | 0.971 | 1.025 |
|  |  |  |  |  |  |  | (0.0496) | (0.0496) | (0.0496) |
| Maori |  |  |  |  |  |  | 1.272 | 0.737*** | 0.783 |
|  |  |  |  |  |  |  | (0.216) | (0.0684) | (0.241) |
| Migrant |  |  |  |  |  |  | 1.09 | 0.932 | 0.961 |
|  |  |  |  |  |  |  | (0.106) | (0.102) | (0.167) |
| Sole Dependent Children |  |  |  |  |  |  | 1.592*** | 1.702*** | 1.378 |
|  |  |  |  |  |  |  | (0.167) | (0.196) | (0.777) |
| Age(25-35) |  |  |  |  |  |  | 0.378*** | 0.875* | 0.973 |
|  |  |  |  |  |  |  | (0.0701) | (0.0683) | (0.139) |
| Employed During Earthquake |  |  |  |  |  |  | 1.116 | 1.161*** | 1.392*** |
|  |  |  |  |  |  |  | (0.12) | (0.0468) | (0.16) |
| Constant | 4.970*** | 1 | 0.151*** | 4.470*** | 1 | 0.137*** | 4.148*** | 0.847* | 0.121*** |
|  | (0.339) | (0.0603) | (0.0165) | (0.396) | (0.0664) | (0.0186) | (0.407) | (0.0762) | (0.0181) |
| Control variables | N | N | N | Y | Y | Y | Y | Y | Y |
| **Observations** | **48,606** | **48,606** | **48,606** | **48,582** | **48,582** | **48,579** | **48,579** | **48,579** | **48,579** |
| *Note: Generalized ordered logistic (GOL) regression model compares all the categories greater than the current category to those less than or equal to the current category (i.e. > vs. ≤ categories comparison). Then we have each column from GOL compare from ( >1 vs <=1) to (>4 vs <=4).* | | | | | | | | | |

**Table 39: gologit – Impact of Earthquake to Safety (Model 3)**

| **Variables** | **>1 vs <=1** | **>2 vs <=2** | **>3 vs <=3** | **>4 vs <=4** | **>1 vs <=1** | **>2 vs <=2** | **>3 vs <=3** | **>4 vs <=4** | **>1 vs <=1** | **>2 vs <=2** | **>3 vs <=3** | **>4 vs <=4** |
| --- | --- | --- | --- | --- | --- | --- | --- | --- | --- | --- | --- | --- |
|  | (1) | (2) | (3) | (4) | (5) | (6) | (7) | (8) | (9) | (10) | (11) | (12) |
| treatment | 1.198 | 1.015 | 0.948 | 0.874 | 1.178 | 0.976 | 0.901 | 0.831 | 1.744* | 1.229* | 0.99 | 0.742 |
|  | (0.192) | (0.112) | (0.0993) | (0.112) | (0.195) | (0.127) | (0.116) | (0.113) | (0.501) | (0.144) | (0.156) | (0.143) |
| treatment#post_EQ | 0.982 | 1.003 | 1.007 | 1.112 | 1.008 | 1.046 | 1.064 | 1.145 | 0.765 | 0.82 | 1.06 | 1.098 |
|  | (0.104) | (0.0674) | (0.068) | (0.103) | (0.0874) | (0.0732) | (0.0852) | (0.108) | (0.185) | (0.117) | (0.123) | (0.224) |
| treatment#post_EQ# |  |  |  |  |  |  |  |  |  |  |  |  |
| Female |  |  |  |  |  |  |  |  | 1.319** | 1.14 | 1.077 | 1.147 |
|  |  |  |  |  |  |  |  |  | (0.164) | (0.144) | (0.077) | (0.2) |
| Maori |  |  |  |  |  |  |  |  | 1.685** | 0.684*** | 0.678*** | 0.653 |
|  |  |  |  |  |  |  |  |  | (0.397) | (0.0687) | (0.0898) | (0.187) |
| Migrant |  |  |  |  |  |  |  |  | 1.111 | 0.978 | 0.810** | 1.407*** |
|  |  |  |  |  |  |  |  |  | (0.0964) | (0.154) | (0.0763) | (0.115) |
| Sole Dependent Children |  |  |  |  |  |  |  |  | 2.356*** | 2.199*** | 1.468*** | 1.262 |
|  |  |  |  |  |  |  |  |  | (0.233) | (0.213) | (0.191) | (0.3) |
| Age(25-35) |  |  |  |  |  |  |  |  | 1.472** | 1.11 | 1.652*** | 1.657* |
|  |  |  |  |  |  |  |  |  | (0.26) | (0.192) | (0.177) | (0.429) |
| Employed During Earthquake |  |  |  |  |  |  |  |  | 0.9 | 1.167 | 0.914 | 0.852 |
|  |  |  |  |  |  |  |  |  | (0.152) | (0.137) | (0.0552) | (0.131) |
| Constant | 7.916*** | 3.929*** | 1.156* | 0.157*** | 22.91*** | 11.76*** | 2.979*** | 0.350*** | 21.41*** | 10.44*** | 3.077*** | 0.385*** |
|  | (0.723) | (0.279) | (0.093) | (0.0163) | (3.917) | (1.366) | (0.321) | (0.0413) | (3.72) | (1.402) | (0.361) | (0.0497) |
| Control variables | N | N | N | N | Y | Y | Y | Y | Y | Y | Y | Y |
| **Observations** | **46,086** | **46,083** | **46,083** | **46,083** | **46,065** | **46,065** | **46,065** | **46,065** | **46,065** | **46,065** | **46,065** | **46,065** |
| *Note: Generalized ordered logistic (GOL) regression model compares all the categories greater than the current category to those less than or equal to the current category (i.e. > vs. ≤ categories comparison). Then we have each column from GOL compare from ( >1 vs <=1) to (>4 vs <=4).* | | | | | | | | | | | | |
